# Importance of the Proximity and Orientation of Ligand-Linkage to the Design of Cinnamate-GW9662 Hybrid Compounds as Covalent PPARγ Agonists

**DOI:** 10.3390/molecules24102019

**Published:** 2019-05-27

**Authors:** Yuki Utsugi, Hirona Kobuchi, Yukio Kawamura, Ahmed Salahelden Aboelhamd Atito, Masaya Nagao, Hiroko Isoda, Yusaku Miyamae

**Affiliations:** 1 College of Agro-Biological Resources Sciences, University of Tsukuba, Ibaraki 305-8572, Japan; utsugi.yuki.sp@alumni.tsukuba.ac.jp; 2 Master’s/Doctoral Program in Life Science Innovation, School of Integrative and Global Majors, University of Tsukuba, Ibaraki 305-8572, Japan; fopcu91@gmail.com (A.S.A.A.); isoda.hiroko.ga@u.tsukuba.ac.jp (H.I.); 3 Department of Food and Nutrition, Faculty of Home Economics, Kyoto Women’s University, Kyoto 605-8501, Japan; kobuchi34@gmail.com (H.K.); kawamury@kyoto-wu.ac.jp (Y.K.); 4 Graduate School of Biostudies, Kyoto University, Kyoto 606-8502, Japan; mnagao@kais.kyoto-u.ac.jp; 5 Alliance for Research on the Mediterranean and North Africa, University of Tsukuba, Ibaraki 305-8572, Japan; 6 Faculty of Life and Environmental Sciences, University of Tsukuba, Ibaraki 305-8572, Japan

**Keywords:** PPARγ, covalent agonist, ligand-linkage, structure-activity relationship

## Abstract

Covalent agonists of PPARγ cause unique receptor conformational changes and behave as selective PPARγ modulators, whereas there are few covalent agonists other than endogenous unsaturated fatty acids metabolites. Previously, we established a cell-based strategy to identify new PPARγ ligands and synthesized a new-type of covalent agonist that possesses the hybrid structure of a plant-derived cinnamic acid derivative and GW9662, a covalent antagonist. Herein, we report six analogues that differ in how the two fragments are linked together. Compounds with a simplified linker showed potent agonistic activity with improved EC_50_ values (less than 5 nM), indicating that close proximity between the two fragments improves binding affinity. When the position of cinnamic acid moiety was placed at 4′ carbon of aniline ring, PPARγ agonist activity was completely abolished. Docking studies suggested that the activation profile likely depends on interaction with the cavity around helix 3, β-sheet, and Ω-loop region in the ligand-binding domain. Furthermore, a cell-based assay revealed that agonist-type compounds activate PPARγ transcription in a manner dependent on covalent linkage with the Cys285 residue leading to prolonged transactivation. This activation feature reflects pharmacological benefits of covalent drugs, suggesting that these hybrid compounds may serve as potential leads for a new-class of covalent PPARγ ligands.

## 1. Introduction

Peroxisome proliferator-activated receptor (PPAR) γ is a member of the nuclear receptor superfamily and is found in adipose tissue and macrophages [1]. PPARγ forms obligate heterodimers with retinoid X receptors (RXRs), which triggers the transcriptional switch of target genes in a ligand-dependent manner [2]. Ligand binding to PPARγ stabilizes their conformation and subsequently modulates cofactor recruitment, resulting in transcriptional activation of downstream genes [3,4] related to glucose metabolism, adipogenesis, and macrophage function [5,6,7]. These functions make PPARγ a therapeutic target for type 2 diabetes and other metabolic diseases such as obesity and atherosclerosis. Thiazolidinedione (TZD), a known full PPARγ agonist, has been used for the treatment of type 2 diabetes, however its use has been limited because of adverse effects such as body weight gain and edema. These side effects are closely related with the undesirable expression of a subset of genes as a result of the complete activation of PPARγ transcription by full agonists [8,9]. Partial PPARγ agonists can selectively modulate the transcription of the downstream gene set, resulting in fewer side effects while retaining beneficial pharmacological effects [10,11]. This is related to the distinct conformational changes caused by the partial agonists, leading to different coactivator recruitment and activation of discrete sets of the downstream genes [12,13].

Recently, our group reported a synthetic covalent PPARγ agonist by using a two-step strategy [14]. The first step involved the identification of a combination of compounds that cooperatively activated the PPARγ, followed by the design and synthesis of a hybrid structure. This strategy is based on the unique character of the PPARγ ligand binding pocket (LBP) that can be simultaneously occupied by multiple ligands because of its large cavity coupled with the synergistic activation of the transcriptional switch of PPARγ [15]. Using this approach, we successfully identified the combination of a synthetic irreversible antagonist, called GW9662 (**1**) [16], and a cinnamic acid derivative (**2**). We synthesized a single molecule containing a hybrid structure of the two (**3**) and determined its subtype-selective partial agonistic activity (EC_50_ value = 14 nM) that was dependent on its covalent linkage with the Cys285 residue. A recent study demonstrated that some types of oxidized fatty acids and their metabolites form covalent bonds with Cys285, effectively stabilizing the conformation of the ligand-binding domain (LBD) and activating the transcriptional switch [17]. Another group demonstrated that a metabolite of oleic acids, which has a nitro moiety, forms a covalent bond with Cys285 and behaves as a selective PPARγ modulator, causing the reduction of the blood glucose level without body weight gain in diabetic model mice [18]. These studies indicate that covalent PPARγ ligands have biological significance and pharmacological potential. However, there are few covalent agonists known other than endogenous ligands like fatty acids metabolites. Our synthesized ligand is a rare covalent PPARγ agonist, although we have not yet clarified its structure-activity relationship.

**Figure 1:**
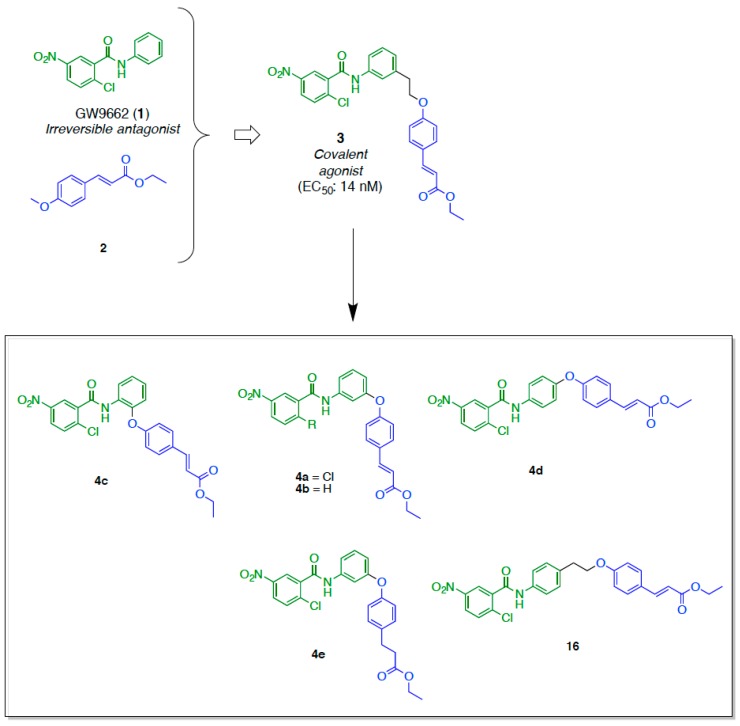
Structures of ligands synthesized in previous and current studies.

The key features of the structural designing of the identified compound were the site and the linker moiety connecting the two fragments. Previously, we selected the hydroxyethyl linker to connect the two units based on the computational analysis. A docking study predicted that the length of the linker should be less than two carbon atoms because linkers with more than three carbons occupy most of the space in the binding subpocket, causing the cinnamate unit to protrude from the space. This prompted us to hypothesize that the proximity between the two fragments is a key factor in determining that the fused hybrid molecule could fit into the available space. As reported herein, we attempted to create a combination where the two fragments were connected with a diphenyl ether moiety instead of an alkyl chain linker (Figure 1). The simplified compounds were synthesized using an improved synthetic route, allowing access to the desired compounds with fewer reaction steps. The improvement of the synthetic protocol also provided us several types of analogues with different orientations of the cinnamate moiety. Using these chemical derivatives, the results indicated that the position of the cinnamate moiety is critical for activation of PPARγ transcription because of the possible interactions with the β-sheet and Ω-loop region. These results provide us with the principles for structural designing by ligand-linking using two identified fragments. Finally, the pharmacological characterization of the newly synthesized covalent agonist was assessed.

## 2. Results and Discussion

### 2.1. Retrosynthetic Analyses

**Scheme 1:**
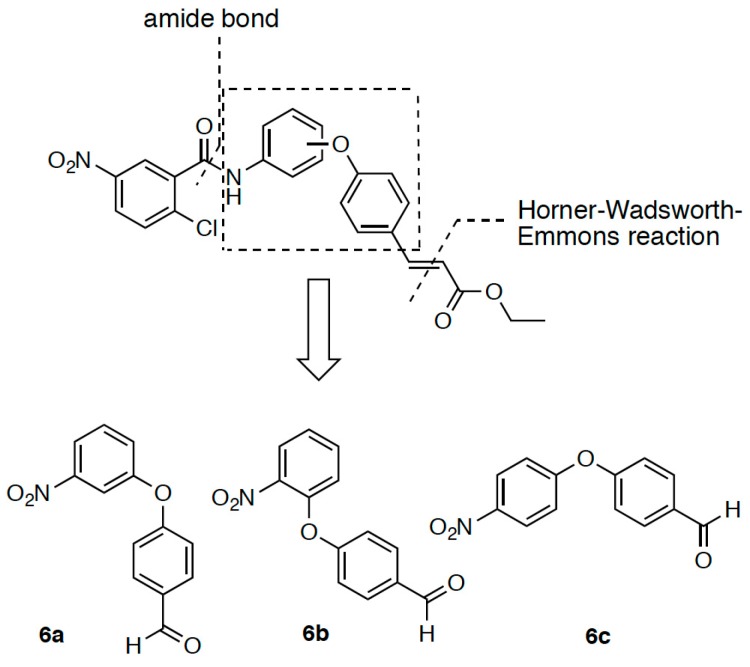
Retrosynthetic analysis.

The simplified analogue **4a** was designed by replacing the hydroxyethyl linker of the previous compound **3** to diphenyl ether. The principle of the synthetic route to **4a**, **4c**, and **4d** is depicted in Scheme 1. The 2-chloro-5-nitrobenzoyl group was planed to be coupled in the last step because of its high reactivity. The amine compound can be obtained by the reduction of nitrobenzene anchored with cinnamic acid ethyl ester. The α,β-unsaturated carbonyl of the cinnamate moiety can be produced by the Horner-Wadsworth-Emmons reaction. The central diphenyl ether was a key intermediate of this study. This can be easily synthesized through one step by using a standard etherification reaction, enabling the number of steps to be reduced. Several types of aromatic ring compounds with different positions of the reactive groups are commercially available, and were used as starting materials to synthesize three types of intermediates, **6a**, **6b**, and **6c**. It allowed us to obtain several analogues with different orientations of the cinnamate moiety.

### 2.2. Synthesis

**Scheme 2:**
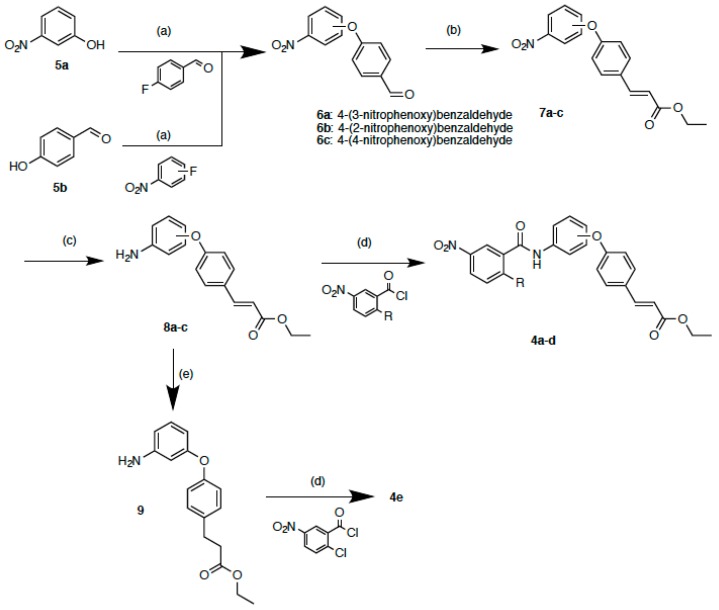
Reagents and conditions: (a) K_2_CO_3_, DMF, 80 °C, 18 h. (b) (EtO)_2_P(O)CH_2_COOEt, LiCl, DBU, CH_3_CN, rt, 18 h. (c) Zn, NH_4_Cl, MeOH, reflux, 1 h. (d) Et_3_N, CH_2_Cl_2_, rt, 18 h. (e) Pd/C, H_2_, EtOAc, rt 6 h.

**Scheme 3:**
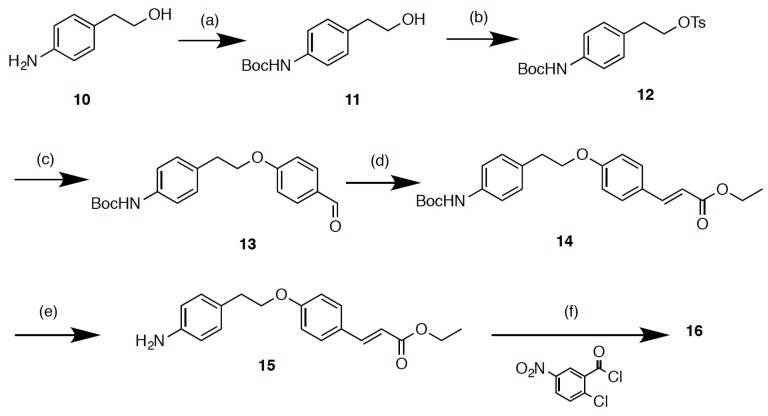
Reagents and condition: (a) ref. [19]. (b) *p*-TsCl, CH_2_Cl_2_, DMAP, Et_3_N, rt, 24 h. (c) K_2_CO_3_, CH_3_CN, *p*-hydroxybenzaldehyde, reflux, 18 h. (d) (EtO)_2_P(O)CH_2_COOEt, LiCl, DBU, CH_3_CN, rt, 18 h. (e) HCl, EtOAc, rt, 18 h. (f) Et_3_N, CH_2_Cl_2_, rt, 18 h.

The syntheses of compounds **4a**–**e** are outlined in Scheme 2. Nitro-benzene and benzaldehyde were linked via an ether bond at one of three positions in the nitro-benzene. The aldehyde groups of the resultant diphenyl ethers were converted to the α,β-unsaturated carbonyl compounds **7a**–**c** via the Horner-Wadsworth-Emmons reaction. After reduction of the nitro groups of **7a**–**c**, amines **8a**–**c** were bound to 2-chloro-5-nitrobenzoyl chloride or 3-nitrobenzoyl chloride to produce **4a**–**d**. **4e** was obtained by the same amide bond formation using **9**, which was produced by catalytic hydrogenation of **8a**. Compound **16** was synthesized using **10** as a starting material, according to the procedure previously reported by our group [14] (Scheme 3). The amino group of **10** was protected with a Boc group using a reported protocol [19]. The alcohol moiety of **11** was tosylated with *p*-toluensulfonyl chloride. The sulfonate compound was connected with *p*-hydroxybenzaldehyde through an ether bond. The aldehyde group of diphenyl ether was converted to the α,β-unsaturated carbonyl compound **14** via the Horner-Wadsworth-Emmons reaction. After removal of the Boc group, amine **15** was bound to 2-chloro-5-nitrobenzoyl chloride to produce **16**.

### 2.3. PPARγ Agonist Activity

**Figure 2:**
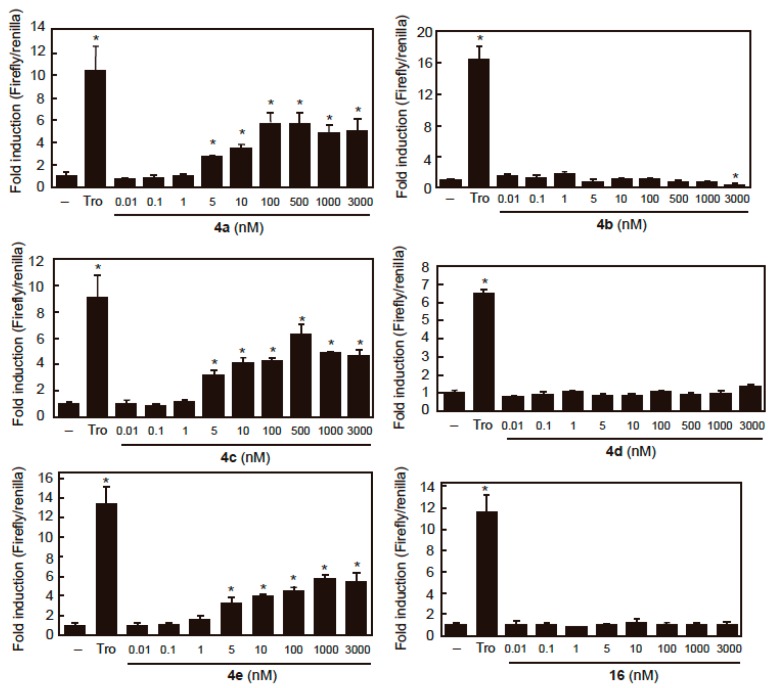
Transactivation potentials of PPARγ by **4a**, **b**, **c**, **d**, **e**, and **16**. The luciferase reporter assay was performed in HepG2 cells transiently cotransfected with pGal4-humanPPARγLBD plasmid, pUAS-tk-Luc reporter, and pGL4.74[hRluc/TK] (Promega). Firefly luciferase activities were normalized to Renilla luciferase activities. Vehicle control data are denoted as 1. HepG2 cells were treated with the vehicle (0.1% DMSO, shown as –), troglitazone (10 μM, shown as Tro), **4a**, **b**, **c**, **d**, **e**, or **16** for 6 h. All the results are presented as the mean ± SD (n = 3) of three independent experiments. * *p* < 0.001, compared with vehicle control.

PPARγ agonist activity was assessed by expression of a GAL4 DNA-binding domain/PPARγ LBD chimera protein using the plasmid pGAL4- PPARγ LBD and the luciferase reporter plasmid pUAS-tk-Luc containing the target sequence of GAL4 [20]. The test results of all the synthesized compounds are shown in Figure 2 and Table 1. Compound **4a** (EC_50_ = 5.13 ± 0.45 nM) and **4c** (EC_50_ = 4.11 ± 0.13 nM) exhibited potent PPARγ agonist activity, which was about 3-fold more potent than a previously synthesized compound **3**. This supports our hypothesis that close proximity between the two fragments contributes to the improvement of binding affinity. Compound **4e** activated PPARγ at a low concentration (EC_50_ = 2.54 ± 1.30 nM) and to a similar extent as **4a** and **4c**, suggesting that the double bond of the α,β-unsaturated carbonyl moiety is not necessary for the agonist activity. The fold changes of the agonist induction activity of these compounds were almost half that of troglitazone, indicating that these compounds act as partial PPARγ agonists. Compound **4b**, in which the chlorine atom of **4a** was substituted with hydrogen, had no agonist activity, indicating that the agonist activity of **4a** requires the covalent bond formation with the Cys285 residue via nucleophilic substitution. Interestingly, when the position of the cinnamic acid moiety was placed at the 4′ carbon of the aniline ring (**4d**), the PPARγ agonist activity was completely abolished. The disappearance of activity was also observed for compound **16**, in which the cinnamic acid moiety was connected with an ethyl linker at the 4′ position of the aniline ring. These results indicate that the connecting position of cinnamic acid is critical for agonist activity.

### 2.4. Validation of Covalent Bond Formation of the Synthesized Compounds with the Cys285 Residue

**Figure 3:**
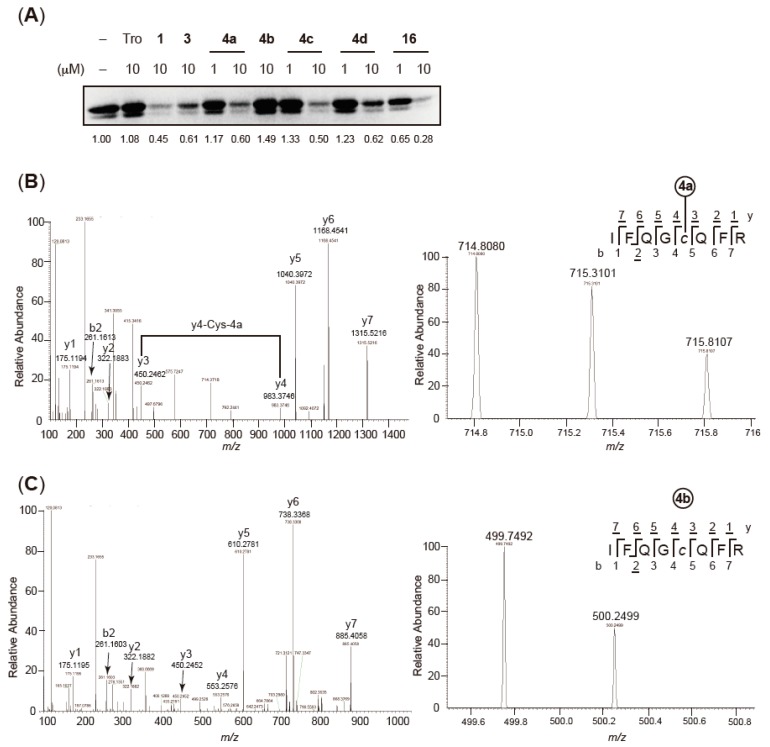
Assessment of the abilities of the synthesized compounds to form a covalent bond with the Cys285 residue in the PPARγ LBD. (**A**) PPARγ LBD recombinant protein was incubated with the vehicle (0.1% DMSO, shown as –), troglitazone (shown as Tro), **1**, **3**, **4a**, **4b**, **4c**, **4d**, or **16**, and the free cysteine residue was detected by the rhodamine-maleimide assay. (**B**), (**C**) Detection of covalent binding of **4a** to Cys285 in the PPARγ LBD by ESI-MS/MS analysis. Fragmentation pattern of peptide IFQGCQFR was obtained from the trypsin digest. The calculated y- and b-ions of this peptide with observed ions underlined and the native or modified peptides detected in chromatogram are shown. The spectrum in (**B**) shows the adduct of **4a** with Cys285 residue in the PPARγ LBD; in contrast, in panel (**C**), no adduct of **4b** is observed.

To assess the interactions between the receptor and the synthesized compounds, we initially validated the formation of a covalent bond with Cys285 by performing a rhodamine-maleimide assay [21]. This assay can detect the presence of a covalent modification of Cys285 in the PPARγ LBD because it has only one cysteine residue in its sequences. As shown in Figure 3A, the fluorescence of the rhodamine-labeled PPARγ LBD decreased after treatment with GW9662 alone. The decrease of labeled PPARγ LBD was also found when it was incubated with agonist-type compounds **4a** and **4c**, indicating that these ligands can form a covalent bond with the Cys285 residue in the PPARγ LBD. Interestingly, non-agonist type ligands **4d** and **16** also showed the decrease of fluorescence, indicating that these two compounds are able to bind to PPARγ LBD through the formation of covalent bond with Cys285 but do not exhibit the binding manners which cause the receptor activation. In contrast, decrease of fluorescence was not observed by the treatment of compound **4b**, which lacks the ability of nucleophilic addition to the Cys285 residue because of the replacement of chlorine to hydrogen. To further confirm the covalent bonding of the synthesized compounds, we conducted proteolytic mapping studies via mass spectroscopy using **4a** as a representative compound and **4b** as a negative control. The complexes between recombinant PPARγ LBD and the test compounds were trypsinized and subjected to MALDI-TOF MS. Covalent binding of **4a** (mass of 466.1 Da) to the PPARγ LBD is assumed to result in a mass change of 431.1 Da for the modified tryptic peptide. By comparison of the tryptic digests of PPARγ, with and without **4a**, a candidate peptide containing the attachment site for **4a** (IFQGCQFR, *m/z* 499.7492 Da) was detected by its mass shift to *m/z* 714.8080 Da (detected as double charged; Figure 3B). The binding site of **4a** was identified as the Cys285 residue using MS/MS analysis to detect the mass difference between the y3- and y4-ions (*m/z* 533.1 Da), as shown in Figure 3B. In contrast, these mass shifts were not exhibited by the non-covalent analogue **4b**. Taken together, these experiments indicated that compounds that possess a 2-chloro-5-nitrobenzoyl moiety form a covalent bond with the Cys285 residue in the PPARγ LBD.

### 2.5. Docking Study

**Figure 4:**
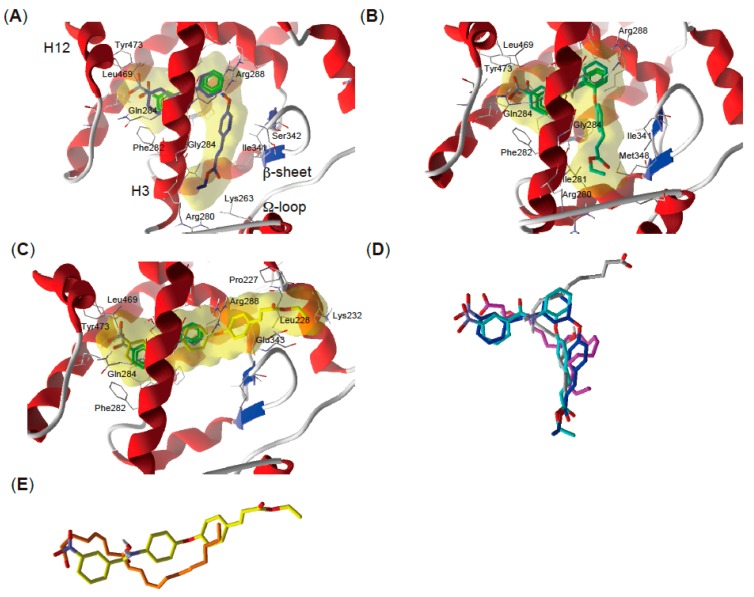
Characterization of the putative binding modes of the synthesized ligands. (**A**–**C**) Co-crystal structure of the complex between the PPARγ LBD and GW9662 (green) (PDB code 3B0R), and docking poses of **4a** (blue, **A**), **4c** (cyan, **B**), and **4d** (yellow, **C**). Yellow spheres indicate hydrophobic contacts. (**D**) Superposition of 15-oxo-ETE (gray, PDB code 2ZK4), 4-oxo-DHA (magenta, PDB code 2VV3), and the docking poses of **4a** and **4c**. (**E**) Superposition of 8-oxo-ETE (orange, PDB code 2ZK3) and the docking pose of **4d**.

To gain further insight into the interaction between the synthesized covalent ligands and the PPARγ LBD, a molecular docking simulation was carried out. The PPARγ LBD comprises 13 helices and a small four-stranded β-sheet. Full agonists such as TZD occupy the binding site around helix 3 and 12, and cause a drastic conformational change of the AF-2 region because of the hydrogen bond with the Tyr473 residue [3]. Partial agonists are shown to bind to the cavity around helix 3, the β-sheets, and Ω-loop region (termed the alternate binding site) [22,23,24] rather than the AF-2 sites. Compound **1** (GW9662) covalently reacted with the Cys285 residue through nucleophilic substitution [16] irreversibly blocking other agonists such as TZDs from entering the AF-2 sites, whereas there was an open space around the alternate binding site. We, and others, revealed that another small ligand, such as **2**, could access the alternate binding site in the GW9662-bound PPARγ LBP [14,24]. To determine the possible binding manner of the newly synthesized ligands, we docked the compounds into the crystal structure of the human PPARγ LBD bound to **1** (PDB code 3B0R). Taking into account the covalent bond formation, the fifth carbon of the nitrobenzene ring in the ligand was constrained to the sulfur atom of the thiol group in Cys285 and then the constrained ligand was docked. Because agonist-type ligands synthesized in this study act as partial agonists, we defined the potential binding site for docking as the cavity near helix3, the β-sheet, and Ω-loop region in the PPARγ LBD rather than the AF-2 pocket near H12. To validate the docking protocol, we first performed cross-docking simulations by using several crystal structures (5DV3, 5DV6, and 5DV8) of the complexes between PPARγ LBD and different types of covalent ligands. We observed that the reported binding manner could be reproduced when the ligands were docked into the original protein structure (Supplementary Figure S1A). Similar binding poses were obtained when the ligands were docked in the crystal structure other than originally reported structural data (Supplementary Figure S1B). The root mean square deviation (RMSD) values of small-size compounds like GW9662, SB1404, and SB1405 were less than 1.2 while that of SB1451, which has huge lipophilic moiety in the anilin ring of GW9662 unit, was less than 1.9. Encouraged by these data, the binding poses of newly synthesized compounds were analyzed by the validated protocol. The binding manner of **1** in the original crystal structure is displayed in green (Figure 4A–C). Compound **1** was located behind helix3 and formed a covalent bond to Cys285. The docking simulation predicted that the GW9662 units of docked compounds **4a**, **4c**, and **4d** interact with Phe282, Gln284, and Arg288 in the helix3 as well as Leu469 and Tyr473 in the helix12. The binding poses of GW9662 units of docked compounds aligned with the binding position of **1** in the original crystal structure, while the binding sites of the cinnamic acid moiety are different among the agonist- and non-agonist-type ligands. In the binding mode of the agonist-type ligands **4a** and **4c**, the cinnamate moiety is able to interact with Arg280, Ile281, and Gly284 in the helix3, and Ile341 and Ser342 in the β-sheet, and Lys263 in the Ω-loop (Figure 4A,B), implying that the region near helix 3 and the Ω-loop may be robustly stabilized by binding with these ligands. In contrast, non-agonist type ligand **4d** exhibited a different binding pose in which the whole structure was positioned orthogonal to the helix 3 axis and the cinnamic acid moiety interact with Pro227, Leu228, and Lys232 in the helix1 as well as Glu343 in the β-sheet, located outside the cavity (Figure 4C). The Mol Dock Scores of these ligands were −175.697 (**4a**), −184.234 (**4c**), and −157.507 (**4d**).

These binding poses of both the agonist and non-agonist types of compounds were similar to those of endogenous unsaturated fatty acids. Previous studies reported that oxidized fatty acids occupy the PPARγ LBP by covalently modifying the Cys285 residue. 15-Oxo-eicosatetraenoic acid (15-oxo-ETE) and 4-oxo-docoxahexanoiec acid (4-oxo-DHA) effectively stabilize the Ω-loop region through the formation of a covalent bond with Cys285, leading to the transcriptional activation [17,25]. The configuration of 15-oxo-ETE is parallel with the helix 3 axis and interacts with the β-sheet, helix 5 and helix 3, while 4-oxo-DHA binds to the LBP in a U-shaped conformation, wrapping around near the Cys285 residue with the interactions with the helix3 and the β-sheet. 8-Oxo-ETE does not activate the PPARγ, although it forms a covalent bond with Cys285 [25]. The configuration of 8-oxo-ETE is orthogonal to the helix 3 axis similar to the binding pose of **4d**. Indeed, the binding poses of the synthesized compounds showed good alignment with those of endogenous ligands (Figure 4D,E). These observations suggested that our synthesized ligands may mimic the binding mode of endogenous fatty acids that covalently bind to the PPARγ LBD.

### 2.6. Characterization of Agonist-Type Covalent Ligands

**Figure 5:**
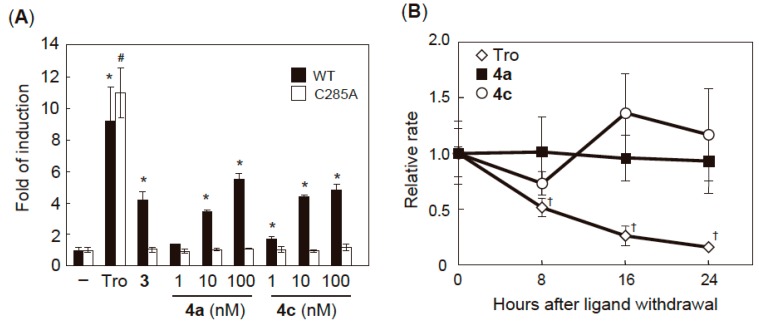
Characterization of the synthesized agonists **4a** and **4c**. (**A**) Mutation of the cysteine residue abolishes the PPARγ agonist activities of **4a** and **4c**. The luciferase reporter assay was performed using wild-type and the C285A mutant of the pGal4-humanPPARγLBD plasmid. The experimental procedure was described in the caption for Figure 2. HepG2 cells were treated with the vehicle (0.1% DMSO, shown as –), troglitazone (10 μM, shown as Tro), **3** (100 nM), **4a** (indicated concentrations), or **4c** (indicated concentrations) for 6 h. * *p* < 0.005, compared with vehicle control (WT). ^#^ *p* < 0.005, compared with vehicle control (C285A). (**B**) Monitoring the agonistic activities after ligand withdrawal. The luciferase reporter assay was performed using the pGal4-humanPPARγLBD plasmid. The experimental procedure was described in the caption for Figure 2. The transfected HepG2 cells were treated with troglitazone (10 μM, shown as Tro), **4a** (100 nM), or **4c** (100 nM) for 6 h and then washed with media to remove the treated compounds. Cells were further cultured for the indicated times in the absence of ligands, after which the luciferase activities were measured. All the results are presented as the mean ± SD (n = 3) of three independent experiments. ^†^ *p* < 0.01, compared with 0 h after ligand withdrawal.

Finally, we assessed the pharmacological properties of the agonist-type covalent ligands. We first examined the relationship between covalent binding to the Cys285 residue and the agonist activity by a luciferase reporter gene assay using PPARγ mutants where the cysteine was substituted to an alanine (C285A). The mutation did not influence the transcriptional response to troglitazone treatment, indicating that the mutated LBD retained its agonist-inducible properties. In the C285A mutant, **4a** and **4c** did not show the transactivation (Figure 5A), indicating that the covalent modification of Cys285 is indispensable for the agonist activity of these compounds. Next, we investigated the potential advantages of covalent binding for pharmacological applications. One of the features of covalent ligands is that the compounds can be anchored to the receptor, mimicking a situation where the drug captures the protein [26]. Based on this consideration, we assumed that the transcription level is related to transient exposure to the ligands and is sustained after ligand elimination. To examine this idea, we monitored the levels of luciferase activity upon withdrawal of the synthesized agonists. In cells treated with troglitazone, a non-covalent agonist, luciferase transcriptional levels decreased upon withdrawal of troglitazone, with luciferase activity approaching background levels after 24 h. In contrast, the treatment of **4a** and **4c** resulted in sustained transcription activity 24 h after withdrawal of the ligands, as expected.

## 3. Materials and Methods

### 3.1. Chemistry

#### 3.1.1. General Remarks

All reagents and solvents were purchased and used without further purification. Flash chromatography was performed using Wako gel C-200 (Fujifilm Wako Pure Chemical Corporation, Osaka, Japan) and Parallel FR-360 (Yamazen Corporation, Osaka, Japan). The following spectroscopic and analytical instruments were used: ^1^H and ^13^C NMR, Avance III 400 (reference TMS, Bruker, Germany), Avance 500 (reference TMS, Bruker, Germany), JNM-ECS 400 (reference TMS, JOEL, Tokyo, Japan), HR-ESI-TOF-MS, Waters Xevo G2-S QTof (Waters, Tokyo, Japan).

#### 3.1.2. 4-(3-Nitrophenoxy)benzaldehyde (**6a**)

K_2_CO_3_ (621 mg, 4.5 mmol) was added to a solution of 3-nitrophenol **5a** (417 mg, 3 mmol) in DMF (12 mL). The reaction mixture was stirred at room temperature for 30 min. Then, *p*-fluorobenzaldehyde (501 mg, 3.6 mmol) was added to the reaction mixture, and stirred at 80 °C for 18 h. The solution was extracted with Hexane/EtOAc (4:1). The organic layer was washed with brine and dried over MgSO_4_, filtered, and the solvents were evaporated in vacuo. The residue was purified by silica gel chromatography (*φ*20 × 150 mm; Hexane/EtOAc, 80:20) to afford **6a** (691 mg, 2.8 mmol, 95%). ^1^H NMR (CDCl_3_, 400 MHz): δ 7.15 (2H, d, *J* = 8.7 Hz), 7.42 (1H, ddd, *J* = 0.9, 2.4, 8.2 Hz), 7.59 (1H, t, *J* = 8.2 Hz), 7.91 (1H, m), 7.93 (2H, d, *J* = 8.8 Hz), 8.07 (1H, ddd, *J* = 0.9, 2.1, 8.2 Hz), 9.98 (1H, s) ppm. ^13^C NMR (CDCl_3_, 100 MHz): δ 114.8, 118.7, 118.7, 119.3, 125.8, 130.8, 132.2, 132.2, 132.6, 149.4, 156.4, 161.3, 190.6 ppm. HR ESI-MS (negative ion) *m/z*: 242.0434 (M − H)^−^ (Calcd for C_13_H_8_NO_4_: 242.0453).

#### 3.1.3. General Procedure of Preparation for Compounds **6b** and **c**

K_2_CO_3_ (850 or 1270 mg, 1.5 eq) was added to a solution of 4-hydroxybenzaldehyde **5b** (500 or 750 mg, 1 eq) in DMF (15 mL). The reaction mixture was stirred at room temperature for 30 min. Then, 4-fluoro-nitrobenzen (690 mg, 1.2 eq) or 1-fluoro-2-nitrobenzen (1040 mg, 1.2 eq) was added to the reaction mixture, and stirred at 80 °C for 18 h. The solution was extracted with Hexane/EtOAc (2:1). The organic layer was washed with brine and dried over MgSO_4_, filtered, and the solvents were evaporated in vacuo. The residue was purified by silica gel chromatography (*φ*20 × 150 mm; Hexane/EtOAc, 80:20) to yield **6b** or **c**.

#### 3.1.4. 4-(2-Nitrophenoxy)benzaldehyde (**6b**)

Colorless liquid, yield 93%. ^1^H NMR (CDCl_3_, 400 MHz): δ 7.10 (2H, d, *J* = 8.6 Hz), 7.21 (1H, dd, *J* = 1.2, 8.2 Hz), 7.38 (1H, ddd, *J* = 1.2, 7.6, 8.1 Hz), 7.65 (1H, ddd, *J* = 1.6, 7.5, 8.2 Hz), 7.90 (2H, d, *J* = 8.6 Hz), 8.04 (1H, dd, *J* = 1.6, 8.2 Hz), 9.96 (1H, s) ppm. ^13^C NMR (CDCl_3_, 100 MHz): δ 117.9, 117.9, 123.3, 125.6, 126.3, 132.2, 132.2, 132.4, 134.9, 142.5, 148.5, 161.9, 190.8 ppm. HR ESI-MS (negative ion) *m/z*: 242.0434 (M − H)^−^ (Calcd for C_13_H_8_NO_4_: 242.0453).

#### 3.1.5. 4-(4-Nitrophenoxy)benzaldehyde (**6c**)

Colorless liquid, yield 93%. ^1^H NMR (CDCl_3_, 400 MHz): δ 7.14 (2H, d, *J* = 9.3 Hz), 7.20 (2H, d, *J* = 8.6 Hz), 7.96 (2H, d, *J* = 8.7 Hz), 8.28 (2H, d, *J* = 9.2 Hz), 10.0 (1H, s) ppm. ^13^C NMR (CDCl_3_, 100 MHz): δ 118.8, 118.8, 119.7, 119.7, 126.1, 126.1, 132.1, 132.1, 133, 143.8, 160.3, 161.3, 190.5 ppm. HR ESI-MS (negative ion) *m/z*: 242.0434 (M − H)^−^ (Calcd for C_13_H_8_NO_4_: 242.0453).

#### 3.1.6. General Procedure of Preparation for Compounds **7a**–**c**

(EtO)_2_P(O)CH_2_COOEt (615 µl, 1.1 eq) was added to LiCl (130 mg, 1.1 eq) and DBU (830 μL) in dry CH_3_CN (10 mL), and stirred at room temperature for 1 h. Then corresponding aldehyde (500 mg, 1 eq) in dry CH_3_CN (1 mL) was added to the solution and stirred at room temperature for 18 h. After addition of water, the solution was extracted with EtOAc. The organic layer was washed with brine and dried over MgSO_4_, filtered, and the solvents were evaporated in vacuo. The residue was purified by silica gel chromatography (*φ*20 × 150 mm; Hexane/EtOAc, 90:10) to yield **7a**–**c**.

#### 3.1.7. (*E*)-Ethyl 3-[4-(3-nitrophenoxy)phenyl]acrylate (**7a**)

Colorless liquid, yield 77%. ^1^H NMR (CDCl_3_, 400 MHz): δ 1.35 (3H, t, *J* = 7.1 Hz), 4.28 (2H, q, *J* = 7.1 Hz), 6.40 (1H, d, *J* = 16 Hz), 7.06 (2H, d, *J* = 8.7 Hz), 7.37 (1H, ddd, *J* = 0.9, 2.3, 8.2 Hz), 7.53 (1H, t, *J* = 8.2 Hz), 7.57 (2H, d, *J* = 8.7 Hz), 7.68 (1H, d, *J* = 16 Hz), 7.85 (1H, t, *J* = 2.3 Hz), 8.00 (1H, ddd, *J* = 0.9, 2.2, 8.2 Hz) ppm. ^13^C NMR (CDCl_3_, 100 MHz): δ 14.3, 60.5, 113.6, 118, 118.4, 119.5, 119.5, 124.8, 130, 130, 130.5, 130.9, 143.3, 149.3, 157.4, 157.5, 166.9 ppm. HR ESI-MS (negative ion) *m/z*: 312.0869 (M − H)^−^ (Calcd for C_17_H_14_NO_5_: 312.0872).

#### 3.1.8. (*E*)-Ethyl 3-[4-(2-nitrophenoxy)phenyl]acrylate (**7b**)

Colorless liquid, yield 94%. ^1^H NMR (CDCl_3_, 400 MHz): δ 1.34 (3H, t, *J* = 7.1 Hz), 4.27 (2H, q, *J* = 7.1 Hz), 6.37 (1H, d, *J* = 16 Hz), 7.02 (2H, d, *J* = 8.7 Hz), 7.11 (1H, dd, *J* = 0.9, 8.1 Hz), 7.28 (1H, td, *J* = 0.8, 8.2 Hz), 7.53 (2H, d, *J* = 8.8 Hz), 7.56 (1H, td, *J* = 1.4, 8.1 Hz), 7.66 (1H, d, *J* = 16 Hz), 7.99 (1H, dd, *J* = 1.5, 8.2 Hz) ppm. ^13^C NMR (CDCl_3_, 100 MHz): δ 14.5, 60.7, 118, 118.9, 118.9, 121.9, 124.4, 126, 130, 130, 130.7, 134.5, 142, 143.5, 149.7, 158, 167.1 ppm. HR ESI-MS (negative ion) *m/z*: 312.0869 (M − H)^−^ (Calcd for C_17_H_14_NO_5_: 312.0872).

#### 3.1.9. (*E*)-Ethyl 3-[4-(4-nitrophenoxy)phenyl]acrylate (**7c**)

Colorless liquid, yield 90%. ^1^H NMR (CDCl_3_, 400 MHz): δ 1.35 (3H, t, *J* = 7.1 Hz), 4.28 (2H, q, *J* = 7.1 Hz), 6.41 (1H, d, *J* = 16 Hz), 7.07 (2H, d, *J* = 9.3 Hz), 7.10 (2H, d, *J* = 8.6 Hz), 7.58 (2H, d, *J* = 8.6 Hz), 7.68 (1H, d, *J* = 16 Hz), 8.23 (2H, d, *J* = 9.3 Hz) ppm. ^13^C NMR (CDCl_3_, 100 MHz): δ 14.5, 60.8, 118, 118, 118.6, 120.7, 120.7, 126.2, 126.2, 130.2, 130.2, 131.8, 132.4, 143.4, 156.7, 162.6, 167 ppm. HR ESI-MS (negative ion) *m/z*: 312.0869 (M − H)^−^ (Calcd for C_17_H_14_NO_5_: 312.0872).

#### 3.1.10. General Procedure of Preparation for Compounds **8a**–**c**

The corresponding nitrobenzene cinnamic acid derivatives (**7a**: 300 mg, **7b**: 500 mg, **7c**: 250 mg, 1 eq) were dissolved in hot MeOH (15 mL). Zinc (522–1044 mg, 10 eq) and NH_4_Cl (641–1282 mg, 20 eq) were added to the solution. The reaction mixture was refluxed for 1 h. Then, the solution was filtered and evaporated. After addition of water, the solution was extracted with EtOAc. The organic layer was washed with brine and dried over MgSO_4_, filtered, and the solvents were evaporated in vacuo. The residue was purified by silica gel chromatography (*φ*20 × 150 mm; Hexane/EtOAc, 75:25) to yield **8a**–**c**.

#### 3.1.11. (*E*)-Ethyl 3-[4-(3-aminophenoxy)phenyl]acrylate (**8a**)

Colorless liquid, yield 92%. ^1^H NMR (CDCl_3_, 400 MHz): δ 1.33 (3H, t, *J* = 7.3 Hz), 3.73 (2H, s), 4.26 (2H, q, *J* = 7.3 Hz), 6.34 (1H, d, *J* = 16.0 Hz), 6.36 (1H, t, *J* = 2.3 Hz), 6.42 (1H, ddd, *J* = 1.0, 2.3, 8.2 Hz), 6.47 (1H, ddd, *J* = 1.0, 2.3, 8.2 Hz), 6.99 (2H, d, *J* = 8.7 Hz), 7.12 (1H, t, *J* = 8.2 Hz), 7.47 (2H, d, *J* = 8.6 Hz), 7.65 (1H, d, *J* = 16 Hz) ppm. ^13^C NMR (CDCl_3_, 125 MHz): δ 14.5, 60.5, 106.4, 109.7, 111, 117.1, 118.7, 118.7, 129.4, 129.8, 129.8, 130.7, 144, 148.4, 157.5, 159.6, 167.3 ppm. HR ESI-MS (positive ion) *m/z*: 284.1282 (M + H)^+^ (Calcd for C_17_H_18_NO_3_: 284.1287).

#### 3.1.12. (*E*)-Ethyl 3-[4-(2-aminophenoxy)phenyl]acrylate (**8b**)

Colorless liquid, yield 97%. ^1^H NMR (CDCl_3_, 400 MHz): δ 1.33 (3H, t, *J* = 7.1 Hz), 3.77 (2H, s), 4.26 (2H, q, *J* = 7.1 Hz), 6.33 (1H, d, *J* = 16.0 Hz), 6.75 (1H, td, *J* = 1.5, 8.0 Hz), 6.84 (1H, dd, *J* = 1.5, 7.9 Hz), 6.92 (1H, dd, *J* = 1.4, 8.0 Hz), 6.95 (2H, d, *J* = 8.7 Hz), 7.03 (1H, td, *J* = 1.5, 8.0 Hz), 7.47 (2H, d, *J* = 8.6 Hz), 7.64 (1H, d, *J* = 16.0 Hz) ppm. ^13^C NMR (CDCl_3_, 100 MHz): δ 14.5, 60.6, 116.9, 116.9, 117.1, 117.1, 119.1, 121.2, 125.9, 129.1, 130, 130, 139.1, 142.3, 144.1, 159.7, 167.4 ppm. HR ESI-MS (positive ion) *m/z*: 284.1282 (M + H)^+^ (Calcd for C_17_H_18_NO_3_: 284.1287).

#### 3.1.13. (*E*)-Ethyl 3-[4-(4-aminophenoxy)phenyl]acrylate (**8c**)

Colorless liquid, yield 82%. ^1^H NMR (CDCl_3_, 400 MHz): δ 1.33 (3H, t, *J* = 7.1 Hz), 3.64 (2H, s), 4.25 (2H, q, *J* = 7.1 Hz), 6.31 (1H, d, *J* = 16 Hz), 6.69 (2H, d, *J* = 8.8 Hz), 6.88 (2H, d, *J* = 8.8 Hz), 6.90 (2H, d, *J* = 8.7 Hz), 7.44 (2H, d, *J* = 8.7 Hz), 7.63 (1H, d, *J* = 16 Hz) ppm. ^13^C NMR (CDCl_3_, 100 MHz): δ 14.5, 60.6, 116.4, 116.5, 116.5, 117.2, 117.2, 121.8, 121.8, 128.5, 129.8, 129.8, 143.5, 144.2, 147.7, 161.2, 167.4 ppm. HR ESI-MS (positive ion) *m/z*: 284.1282 (M + H)^+^ (Calcd for C_17_H_18_NO_3_: 284.1287).

#### 3.1.14. Ethyl 3-[4-(3-aminophenoxy)phenyl]propanoate (**9**)

**8a** (150 mg, 0.48 mmol) was diolved in EtOAc (5 mL). Following to addition of Pd/C (50 mg), the reaction mixture was stirred at room temperature under a H_2_ atmosphere. After 6 h, Pd/C was removed with celite and the filtrate was evaporated in vacuo. The residue was purified by silica gel chromatography (*φ*10 × 300 mm; Hexane/EtOAc, 80:20) to afford **9** (127 mg, 0.45 mmol, 93%). ^1^H NMR (CDCl_3_, 400 MHz): δ 1.24 (3H, t, *J* = 7.1 Hz), 2.61 (2H, t, *J* = 7.5 Hz), 2.92 (2H, t, *J* = 7.5 Hz), 3.65 (2H, s), 4.13 (2H, q, *J* = 7.1 Hz), 6.30 (1H, t, *J* = 2.2 Hz), 6.37 (1H, ddd, *J* = 0.9, 2.3, 8.1 Hz), 6.40 (1H, ddd, *J* = 0.8, 2.2, 8.0 Hz), 6.93 (2H, d, *J* = 8.6 Hz), 7.07 (1H, t, *J* = 8.0 Hz), 7.15 (2H, d, *J* = 8.6 Hz) ppm. ^13^C NMR (CDCl_3_, 100 MHz): δ 14.4, 30.4, 36.3, 60.6, 105.5, 108.8, 110.1, 119.4, 119.4, 129.6, 129.6, 130.5, 135.6, 148.2, 155.6, 158.8, 173.1 ppm. HR ESI-MS (positive ion) *m/z*: 286.1450 (M + H)^+^ (Calcd for C_17_H_20_NO_3_: 286.1443).

#### 3.1.15. General Procedure of Preparation for Compounds **4a**–**e**

Et_3_N (19–25 μL) in dry CH_2_Cl_2_ (5–8 mL) was added to the solution of corresponding amine (**8a**: 100 or 40 mg, **8b**: 100 mg, **8c**: 75 mg, **9**: 50 mg, 1 eq), and stirred at room temperature until dissolved. Then, 2-chloro-5-nitrobenzoyl chloride (42–85 mg, 1.1 eq) or 3-nitrobenzoyl chloride (24 mg, 1.1 eq) was added to the solution and stirred at room temperature for 18 h. After addition of water, the solution was extracted with CHCl_3_. The organic layer was washed with brine and dried over MgSO_4_, filtered, and the solvents were evaporated in vacuo. The residue was purified by silica gel chromatography (*φ*10 × 300 mm; Hexane/EtOAc, 75:25) to yield **4a**–**e**.

#### 3.1.16. (*E*)-Ethyl 3-{4-[3-(2-chloro-5-nitrobenzamido)phenoxy]phenyl}acrylate (**4a**)

Yellow powder, yield 88%. ^1^H NMR (CDCl_3_, 400 MHz): δ 1.32 (3H, t, *J* = 7.1 Hz), 4.23 (2H, q, *J* = 7.1 Hz), 6.32 (1H, d, *J* = 16.0 Hz), 6.88 (1H, ddd, *J* = 1.6, 2.2, 7.7 Hz), 7.02 (2H, d, *J* = 8.6 Hz), 7.37 (1H, t, *J* = 7.8 Hz), 7.41 (2H, m), 7.48 (2H, d, *J* = 8.7 Hz), 7.61 (1H, d, *J* = 16 Hz), 7.62 (1H, d, *J* = 8.9 Hz), 8.20 (1H, m), 8.22 (1H, dd, *J* = 2.7, 8.8 Hz), 8.53 (1H, d, *J* = 2.7 Hz) ppm. ^13^C NMR (CDCl_3_, 100 MHz): δ 14.3, 60.5, 111.5, 115.6, 116.2, 117.1, 118.8, 118.8, 125.1, 126, 129.6, 129.8, 129.8, 130.4, 131.6, 136.3, 137.6, 138.5, 143.7, 146.5, 157, 158.8, 162.3, 167.2 ppm. HR ESI-MS (negative ion) *m/z*: 465.0863 (M − H)^−^ (Calcd for C_24_H_18_N_2_O_6_Cl: 465.0853).

#### 3.1.17. (*E*)-Ethyl 3-{4-[3-(3-nitrobenzamido)phenoxy]phenyl}acrylate (**4b**)

Colorless powder, yield 55%. ^1^H NMR (CDCl_3_, 400 MHz): δ 1.32 (3H, t, *J* = 7.1 Hz), 4.23 (2H, q, *J* = 7.1 Hz), 6.29 (1H, d, *J* = 16 Hz), 6.85 (1H, ddd, *J* = 0.7, 2.2, 8.2 Hz), 6.98 (2H, d, *J* = 8.7 Hz), 7.32 (1H, t, *J* = 8.1 Hz), 7.43 (2H, d, *J* = 8.7 Hz), 7.45 (2H, m), 7.58 (1H, d, *J* = 16 Hz), 7.64 (1H, t, *J* = 8.1 Hz), 8.24 (1H, dd, *J* = 1.1, 7.9 Hz), 8.34 (1H, ddd, *J* = 1.0, 2.2, 8.2 Hz), 8.68 (2H, m) ppm. ^13^C NMR (CDCl_3_, 100 MHz): δ 14.5, 60.8, 111.9, 116.2, 116.2, 117.2, 119, 119, 122.2, 126.6, 129.7, 130, 130, 130.2, 130.6, 133.8, 136.5, 139.2, 144.1, 148.3, 157.1, 159.1, 163.8, 167.5 ppm. HR ESI-MS (negative ion) *m/z*: 431.1250 (M − H)^−^ (Calcd for C_24_H_19_N_2_O_6_: 431.1243).

#### 3.1.18. (*E*)-Ethyl 3-{4-[2-(2-chloro-5-nitrobenzamido)phenoxy]phenyl}acrylate (**4c**)

Colorless powder, yield 99%. ^1^H NMR (CDCl_3_, 400 MHz): δ 1.33 (3H, t, *J* = 7.1 Hz), 4.25 (2H, q, *J* = 7.1 Hz), 6.35 (1H, d, *J* = 15.9 Hz), 6.99 (1H, m), 7.02 (2H, d, *J* = 8.6 Hz), 7.17 (1H, td, *J* = 1.6, 8.1 Hz), 7.27 (1H, m), 7.51 (2H, d, *J* = 8.6 Hz), 7.59 (1H, d, *J* = 8.8 Hz), 7.64 (1H, d, *J* = 16 Hz), 8.23 (1H, dd, *J* = 2.7, 8.8 Hz), 8.57 (1H, dd, *J* = 1.4, 8.1 Hz), 8.58 (1H, s), 8.61 (1H, d, *J* = 2.7 Hz) ppm. ^13^C NMR (CDCl_3_, 100 MHz): δ 14.3, 60.5, 117.7, 118.3, 118.3, 118.9, 121.7, 125, 125.4, 125.9, 126.1, 129.3, 129.9, 129.9, 130.3, 131.7, 136, 137.3, 143.3, 145.2, 146.7, 158.2, 161.8, 166.9 ppm. HR ESI-MS (negative ion) *m/z*: 465.0863 (M − H)^−^ (Calcd for C_24_H_18_N_2_O_6_Cl: 465.0853).

#### 3.1.19. (*E*)-Ethyl 3-{4-[4-(2-chloro-5-nitrobenzamido)phenoxy]phenyl}acrylate (**4d**)

Colorless powder, yield 79%. ^1^H NMR (CDCl_3_, 400 MHz): δ 1.33 (3H, t, *J* = 7.1 Hz), 4.24 (2H, q, *J* = 7.1 Hz), 6.32 (1H, d, *J* = 16.0 Hz), 6.99 (2H, d, *J* = 8.6 Hz), 7.08 (2H, d, *J* = 8.9 Hz), 7.48 (2H, d, *J* = 8.8 Hz), 7.62 (1H, d, *J* = 16.0 Hz), 7.64 (1H, d, *J* = 8.8 Hz), 7.65 (2H, d, *J* = 8.9 Hz), 8.19 (1H, s), 8.24 (1H, dd, *J* = 2.7, 8.8 Hz), 8.55 (1H, *J* = 2.7 Hz) ppm. ^13^C NMR (CDCl_3_, 100 MHz): δ 14.5, 60.7, 117.2, 118.5, 118.5, 120.6, 120.6, 122.4, 122.4, 125.4, 126.1, 129.6, 130, 130, 131.8, 133.3, 136.7, 137.8, 144.0, 146.8, 153.6, 159.6, 162.5, 167.4 ppm. HR ESI-MS (negative ion) *m/z*: 465.0863 (M − H)^−^ (Calcd for C_24_H_18_N_2_O_6_Cl: 465.0853).

#### 3.1.20. Ethyl 3-{4-[3-(2-chloro-5-nitrobenzamido)phenoxy]phenyl}propanoate (**4e**)

Colorless powder, yield 86%. ^1^H NMR (CDCl_3_, 400 MHz): δ 1.23 (3H, t, *J* = 7.1 Hz), 2.59 (2H, t, *J* = 7.8 Hz), 2.89 (2H, t, *J* = 7.7 Hz), 4.09 (2H, q, *J* = 7.1 Hz), 6.78 (1H, ddd, *J* = 1.0, 2.2, 8.0 Hz), 6.94 (2H, d, *J* = 8.6 Hz), 7.14 (2H, d, *J* = 8.6 Hz), 7.24 (1H, t, *J* = 2.1 Hz), 7.28 (1H, t, *J* = 8.0 Hz), 7.33 (1H, m), 7.57 (1H, d, *J* = 8.8 Hz), 8.16 (1H, dd, *J* = 2.7, 8.8 Hz), 8.39 (1H, s), 8.40 (1H, d, *J* = 2.7 Hz) ppm. ^13^C NMR (CDCl_3_, 100 MHz): δ 14.4, 30.4, 36.1, 60.7, 110.7, 114.9, 115.3, 119.5, 119.5, 125, 126, 129.8, 129.8, 130.3, 131.6, 136.1, 136.7, 137.9, 138.5, 146.5, 155.1, 158.3, 162.7, 173.2 ppm. HR ESI-MS (negative ion) *m/z*: 467.1017 (M − H)^−^ (Calcd for C_24_H_20_N_2_O_6_Cl: 467.1010).

#### 3.1.21. *tert-*Butyl *N*-(4-{2-[(4-methylbenzenesulfonyl)oxy]ethyl}phenyl)carbamate (**12**)

The solution of **11** (209 mg, 1 mmol) in dry CH_2_Cl_2_ (5 mL) was added to *p*-toluensulfonyl chloride (210 mg, 1.1 mmol) with 4-dimethylaminopyridine (12 mg, 0.1 mmol) and Et_3_N (279 μL, 2 mmol) in dry CH_2_Cl_2_ (7 mL) at 0 °C. Then, the reaction mixture was stirred at room temperature for 24 h. After addition of water, the solution was extracted with CH_2_Cl_2_. The organic layer was washed with brine and dried over MgSO_4_, filtered, and the solvents were evaporated in vacuo. The residue was purified by silica gel chromatography (*φ*20 × 150 mm; Hexane/EtOAc, 90:10) to afford **12** (293 mg, 0.81 mmol, 81%). ^1^H NMR (CDCl_3_, 400 MHz): δ 1.51 (9H, s), 2.42 (3H, s), 2.87 (2H, t, *J* = 7.0 Hz), 4.16 (2H, t, *J* = 7.0 Hz), 6.64 (1H, s), 6.99 (2H, d, *J* = 8.5 Hz), 7.24 (2H, d, *J* = 8.5 Hz), 7.27 (2H, d, *J* = 8.5 Hz), 7.67 (2H, d, *J* = 8.5 Hz) ppm. ^13^C NMR (CDCl_3_, 100 MHz): δ 21.6, 28.3, 28.3, 28.3, 34.7, 70.7, 80.5, 118.7, 118.7, 127.8, 127.8, 129.4, 129.4, 129.8, 129.8, 130.7, 132.9, 137.2, 144.6, 152.7 ppm. HR ESI-MS (positive ion) *m/z*: 430.1073 (M + K)^+^ (Calcd for C_20_H_25_NO_5_SK: 430.1091).

#### 3.1.22. *tert-*Butyl *N*-{4-[2-(4-formylphenoxy)ethyl]phenyl}carbamate (**13**)

K_2_CO_3_ (142 mg, 1 mmol) was added to a solution of **12** (250 mg, 0.69 mmol) in dry CH_3_CN (7 mL) at room temperature. The reaction mixture was stirred at room temperature for 20 min. *p*-hydroxybenzaldehyde (101 mg, 0.83 mmol) was added to the reaction mixture at room temperature. The reaction mixture was refluxed for 18 h and cooled to room temperature. The solution was added H_2_O, and extracted with CHCl_3_. The organic layer was washed with brine and dried over MgSO_4_, filtered, and the solvents were evaporated in vacuo. The residue was purified by silica gel chromatography (*φ*20 × 150 mm; Hexane/EtOAc, 90:10) to afford **13** (220 mg, 0.69 mmol, quant.). ^1^H NMR (CDCl_3_, 400 MHz): δ 1.51 (9H, s), 3.07 (2H, t, *J* = 7.0 Hz), 4.21 (2H, t, *J* = 7.0 Hz), 6.65 (1H, s), 6.98 (2H, d, *J* = 8.7 Hz), 7.20 (2H, d, *J* = 8.5 Hz), 7.32 (2H, d, *J* = 8.4 Hz), 7.81 (2H, d, *J* = 8.8 Hz), 9.87 (1H, s) ppm. ^13^C NMR (CDCl_3_, 100 MHz): δ 28.5, 28.5, 28.5, 35.1, 69.5, 81, 115.5, 115.5, 119.6, 119.6, 130.3, 130.3, 130.7, 132.8, 132.8, 133.1, 137.9, 153.8, 164.9, 192 ppm. HR ESI-MS (negative ion) *m/z*: 340.1522 (M − H)^−^ (Calcd for C_20_H_22_NO_4_: 340.1549).

#### 3.1.23. (*E*)-Ethyl 3-{4-[2-(4-{[(*tert*-butoxy)carbonyl]amino}phenyl)ethoxy]phenyl}acrylate (**14**)

(EtO)_2_P(O)CH_2_COOEt (144 μL, 0.72 mmol) was added to LiCl (31 mg, 0.72 mmol) and 1,8-diazabicyclo [5.4.0]undec-7-ene (200 μL) in dry CH_3_CN (4 mL), and stirred at room temperature for 1 h. Then **13** (150 mg, 0.48 mmol) in dry CH_3_CN (1 mL) was added to the solution and stirred at room temperature for 18 h. After addition of water, the solution was extracted with EtOAc. The organic layer was washed with brine and dried over MgSO_4_, filtered, and the solvents were evaporated in vacuo. The residue was purified by silica gel chromatography (*φ*20 × 150 mm; Hexane/EtOAc, 90:10) to afford **14** (179 mg, 0.44 mmol, 91%). ^1^H NMR (CDCl_3_, 400 MHz): δ 1.32 (3H, t, *J* = 7.1 Hz), 1.51 (9H, s), 3.04 (2H, t, *J* = 7.0 Hz), 4.14 (2H, t, *J* = 7.0 Hz), 4.25 (2H, q, *J* = 7.1 Hz), 6.30 (1H, d, *J* = 15.9 Hz), 6.54 (1H, s), 6.87 (2H, d, *J* = 8.8 Hz), 7.19 (2H, d, *J* = 8.5 Hz), 7.31 (2H, d, *J* = 8.4 Hz), 7.44 (2H, d, *J* = 8.7 Hz), 7.63 (1H, d, *J* = 15.9 Hz) ppm. ^13^C NMR (CDCl_3_, 100 MHz): δ 14.3, 28.7, 28.7, 28.7, 34.9, 60.2, 68.8, 80.4, 114.8, 114.8, 115.7, 118.8, 118.8, 127.2, 129.4, 129.4, 129.6, 129.6, 132.5, 136.9, 144.2, 152.8, 160.5, 167.3 ppm. HR ESI-MS (positive ion) *m/z*: 450.1684 (M + K)^+^ (Calcd for C_24_H_29_NO_5_K: 450.1683).

#### 3.1.24. (*E*)-Ethyl 3-{4-[2-(4-aminophenyl)ethoxy]phenyl}acrylate (**15**)

4N HCl/EtOAc (2 mL) was added to a solution of **14** (107 mg, 0.27 mmol) in EtOAc (1 mL) at room temperature. The reaction mixture was stirred at room temperature for 18 h. Then, the solvents were evaporated in vacuo to afford **15** (83 mg, 0.27 mmol, quant.). ^1^H NMR (DMSO-*d*_6_, 400 MHz): δ 1.25 (3H, t, *J* = 7.1 Hz), 3.07 (2H, t, *J* = 6.6 Hz), 4.17 (2H, q, *J* = 7.2 Hz), 4.25 (2H, t, *J* = 6.6 Hz), 6.47 (1H, d, *J* = 16 Hz), 6.97 (2H, d, *J* = 8.8 Hz), 7.31 (2H, d, *J* = 8.4 Hz), 7.42 (2H, d, *J* = 8.3 Hz), 7.60 (1H, d, *J* = 16 Hz), 7.65 (2H, d, *J* = 8.8 Hz), 10.2 (2H, s) ppm. ^13^C NMR (DMSO-*d*_6_, 100 MHz): δ 14.2, 34.2, 59.8, 68, 114.9, 114.9, 114.9, 114.9, 115.5, 122.8, 126.7, 130.1, 130.1, 130.1, 130.1, 137.8, 144.1, 160.2, 166.4 ppm. HR ESI-MS (positive ion) *m/z*: 312.1585 (M + H)^+^ (Calcd for C_19_H_22_NO_3_: 312.1600).

#### 3.1.25. (*E*)-Ethyl 3-(4-{2-[4-(2-chloro-5-nitrobenzamido)phenyl]ethoxy}phenyl)acrylate (**16**)

Et_3_N (50 μL) in dry CH_2_Cl_2_ (2 mL) was added to the solution of **15** (40 mg, 0.12 mmol), and stirred at room temperature until dissolved. Then, 2-chloro-5-nitrobenzoyl chloride (28 mg, 0.13 mmol) was added to the solution and stirred at room temperature for 18 h. After addition of water, the solution was extracted with CHCl_3_. The organic layer was washed with brine and dried over MgSO_4_, filtered, and the solvents were evaporated in vacuo. The residue was purified by silica gel chromatography (*φ*10 × 300 mm; Hexane/EtOAc, 75:25) to afford **16** (38 mg, 77 µmol, 67%). ^1^H NMR (CDCl_3_, 400 MHz): δ 1.32 (3H, t, *J* = 7.1 Hz), 3.10 (2H, t, *J* = 6.8 Hz), 4.20 (2H, t, *J* = 6.8 Hz), 4.23 (2H, q, *J* = 7.1 Hz), 6.27 (1H, d, *J* = 16 Hz), 6.90 (2H, d, *J* = 8.7 Hz), 7.29 (2H, d, *J* = 8.4 Hz), 7.44 (2H, d, *J* = 8.7 Hz), 7.59 (1H, d, *J* = 8.8 Hz), 7.60 (1H, d, *J* = 15.9 Hz), 7.61 (2H, d, *J* = 8.4 Hz), 8.19 (1H, dd, *J* = 2.7, 8.8 Hz), 8.41 (1H, s), 8.52 (1H, d, *J* = 2.7 Hz) ppm. ^13^C NMR (CDCl_3_, 100 MHz): δ 14.3, 35.1, 60.3, 68.6, 114.8, 114.8, 115.7, 120.5, 120.5, 125, 125.7, 127.2, 129.7, 129.7, 129.7, 129.7, 131.4, 135.2, 135.6, 136.7, 137.7, 144.2, 146.4, 160.5, 162.3, 167.3 ppm. HR ESI-MS (negative ion) *m/z*: 493.1168 (M − H)^−^ (Calcd for C_26_H_22_N_2_O_6_Cl: 493.1166).

### 3.2. Cell Line and Cell Culture

The human hepatocyte carcinoma cell line, HepG2 (RCB1886), was provided by the RIKEN BRC through the National Bio-Resource project of the Ministry of Education, Culture, Sports, Science and Technology (MEXT), Japan. HepG2 cells were maintained in Dulbecco’s modified Eagle’s medium (Sigma, St. Louis, MO, USA) supplemented with 10% fetal bovine serum, 100 μg/mL penicilin, and 100 units/mL streptomycin (Sigma) at 37 °C in a humidified 5% CO_2_ atmosphere.

### 3.3. Plasmids and Recombinant Protein

pGal4-human PPARγLBD and pGal4-human PPARγLBD-Cys285Ala were constructed as previously described [14]. Recombinant PPARγLBD protein was kindly provided by Dr. Takuji Oyama (Yamanashi University).

### 3.4. PPARγ Reporter Assay

The PPARγ reporter assay was carried out as previously reported [20] with slight modifications. pGal4-human PPARγLBD, pUAS-tk-luc and Renilla luciferase transfection control vector (pGL4.74, Promega, Madison, WI, USA) were transfected into HepG2 cells using Hily-max (Dojindo, Kumamoto, Japan) and PPARγ agonist activity was determined from the luciferase activity using the Dual-Luciferase Reporter Assay System (Promega) according to the manufacturer’s protocol.

### 3.5. Rhodamine-Maleimide Assay.

Rhodamine-Maleimide assay was performed as previously described [14]. Briefly, recombinant PPARγ LBD (2 µM) was mixed with various ligands at the indicated concentrations in 20 mM Tris-HCl, pH8.0, 150 mM NaCl, and 1 mM tris(2-carboxylethyl)phosphine (TCEP). The mixture was incubated for 30 min at room temperature. SDS was added to the reaction mixture to a final concentration of 0.5%, followed by rhodamine red C_2_ maleimide (Life Technologies, Carlsbad, CA, USA) at a final concentration of 1 mM. After incubation at room temperature for 30 min, samples were treated with SDS-PAGE sample buffer containing β-mercaptoethanol for 30 min at 55 °C and were separated by SDS-PAGE. Fluorescent signals were visualized using the LAS1000 plus image analyzer (Fujifilm, Tokyo, Japan).

### 3.6. LC-MS/MS Detection of the Hybrid Ligand Modification of PPARγ

LC-MS/MS detection was performed as previously described [14]. Briefly, purified recombinant PPARγ LBD (20 µM) was incubated with ligands (40 µM) in 20 mM Tris-HCl, pH8.0, 150 mM NaCl, and 1 mM TCEP for 30 min. Reacted proteins were denatured in SDS sample buffer and subjected to SDS-PAGE followed by Coomassie brilliant blue (CBB) staining. After removing CBB, the excised gel slices were incubated with 25 mM NH_4_HCO_3_ with sequencing grade-modified trypsin at 37 °C for 12 h. After in-gel digestion, extracted peptides were subjected to analysis with a Q Exactive Plus (Thermo Fisher Scientific, Waltham, MA, USA).

### 3.7. Docking Studies

Molecular docking of the compounds against the human PPARγLBD was performed using Molegro Virtual Docker ver. 6.0.1 (CLC bio, Aarhus, Denmark). The ligand structures were drawn using Marvin Sketch 5.11.5. The protein models and compounds to be docked were imported into the docking program according to the software’s instructions. Potential ligand-binding sites of proteins were calculated using the Molegro cavity detection algorithm. For prediction of the binding site of the compounds, the crystal structures of the human PPARγ LBD and GW9662 (PDB code 3B0R) was used for docking of **4a**, **c**, and **d**. Because the chlorine atom in the GW9662 unit of the compounds is eliminated by nucleophilic substitution, we docked the ligand using a structure in which the chlorine atom of the compounds was substituted with a hydrogen atom. Taking into account this covalent bond formation, constraint-based docking was performed according to the software’s instructions. Briefly, the fifth carbon of the nitrobenzene ring in the ligand was constrained to the sulfur atom of the thiol group in Cys285 and then the constrained ligand was docked to the crystal structure of the human PPARγ LBD.

### 3.8. Statistics

All data are depicted as means ± SD. Statistical significance was determined by Student’s *t*-test and accepted at *p* < 0.05.

## 4. Conclusions

In conclusion, we synthesized new derivatives of the covalent agonist **3** using an improved synthetic protocol, allowing us to access a variety of compounds with a simplified linker moiety and a different connection site between GW9662 and the cinnnamate unit. A cell-based assay revealed that the synthesized ligands showed distinct profiles of transactivation of the PPARγ. Compounds **4a** and **4c**, in which cinnamate was linked with a diphenyl ether linker at the 2′ or 3′ position of the aniline ring in GW9662, showed about 3-fold more potent agonist activities than the previously synthesized compound **3**, indicating that the close proximity of GW9662 and cinnamate may improve the binding affinity. In contrast, compounds **4d** and **16**, in which the cinnamate unit was placed at the 4′ position of the aniline ring, exhibited no agonistic activity. By using docking simulation, we reasoned that the different activation profiles are likely to depend on the interaction between the cinnamate moiety and the Ω-loop region in the PPARγ LBD. Interestingly, docking studies predicted that the synthesized compounds may mimic the binding modes of endogenous covalent ligands like oxidized fatty acids with corresponding activity profiles. Because we have not yet fully investigated what cellular responses are caused by the formation of a covalent bond with Cys285, our synthesized ligands can serve as chemical probes to assess these biological questions. We further characterized the synthesized compounds and found that ligands **4a** and **4c** partially activated PPARγ transcription in a manner dependent on covalent modification with the Cys285 residue in the PPARγ LBD. The abilities of **4a** and **4c** to form a covalent bond could also result in two ligands being anchored in the LBP, causing prolonged transactivation. This feature reflects one of the advantages of covalent drugs, including prolonged duration of action and less-frequent drug dosing [27], although the safety of compounds that participate in covalent modification needs to be clarified using an *in vivo* model. Further biological and pharmacological studies will enhance the development of new-types of PPARγ ligands with the ability to form covalent bonds.

## Figures and Tables

**Table 1:** EC_50_ values of tested compounds.

Compounds	EC_50_ (nM)
**4a**	5.13 ± 0.45
**4b**	N.D.
**4c**	4.11 ± 0.13
**4d**	N.D.
**4e**	2.54 ± 1.30
**16**	N.D.

## Supplementary Materials

The Supplementary Materials are available online.
