# Supplementary material for: Importance of the Proximity and Orientation of Ligand-Linkage to the Design of Cinnamate-GW9662 Hybrid Compounds as Covalent PPARγ Agonists
Source: Molecules. 2019 May 27;24(10):2019. doi: 10.3390/molecules24102019 (PMC6571965; doi:10.3390/molecules24102019)

Supplementary Materials

Importance of the proximity and orientation of ligand-linkage to the design of cinnamate-GW9662 hybrid compounds as covalent PPARγ agonists

Yuki Utsugi[1,2], Hirona Kobuchi[3], Yukio Kawamura[3], Ahmed Salahelden Aboelhamd Atito[2], Masaya Nagao[4], Hiroko Isoda[2,5,6], and Yusaku Miyamae[2,4,6]*

[a]College of Agro-Biological Resources Sciences, University of Tsukuba, [b]Master's/Doctoral Program in Life Science Innovation, School of Integrative and Global Majors, University of Tsukuba, [c]Department of Food and Nutrition, Faculty of Home Economics, Kyoto Women's University, [d]Graduate School of Biostudies, Kyoto University, [e]Alliance for Research on the Mediterranean and North Africa, University of Tsukuba, [f]Faculty of Life and Environmental Sciences, University of Tsukuba. E-mail: miyamae.yusaku.fw@u.tsukuba.ac.jp

Table of Contents

1. Supplementary Figure 1..2
2. ^{1}H and ^{13}C NMR spectra...3

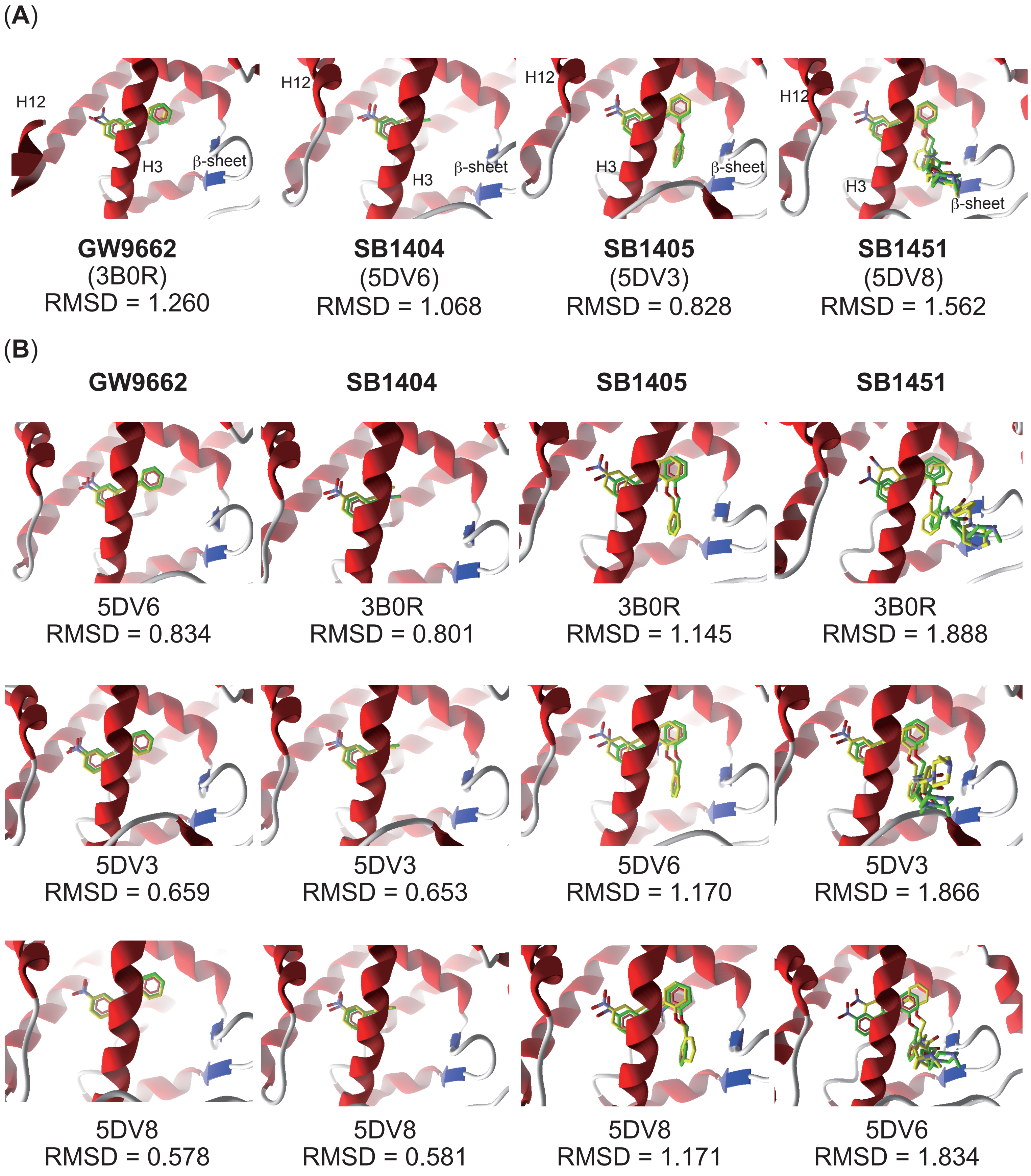

Supplemntary Figure 1. Validation of docking protocol. (A) The indicated ligands were docked into the original protein crystal structures. The reported binding manners and predicted docking poses were displayed in green and yellow, respectively. (B) The ligands were docked in the crystal structures other than originally reported structural data. The reported binding manners and predicted docking poses were displayed in green and yellow, respectively.

400 MHz ^{1}H NMR spectrum of compound **6a** in $CDCl_3$

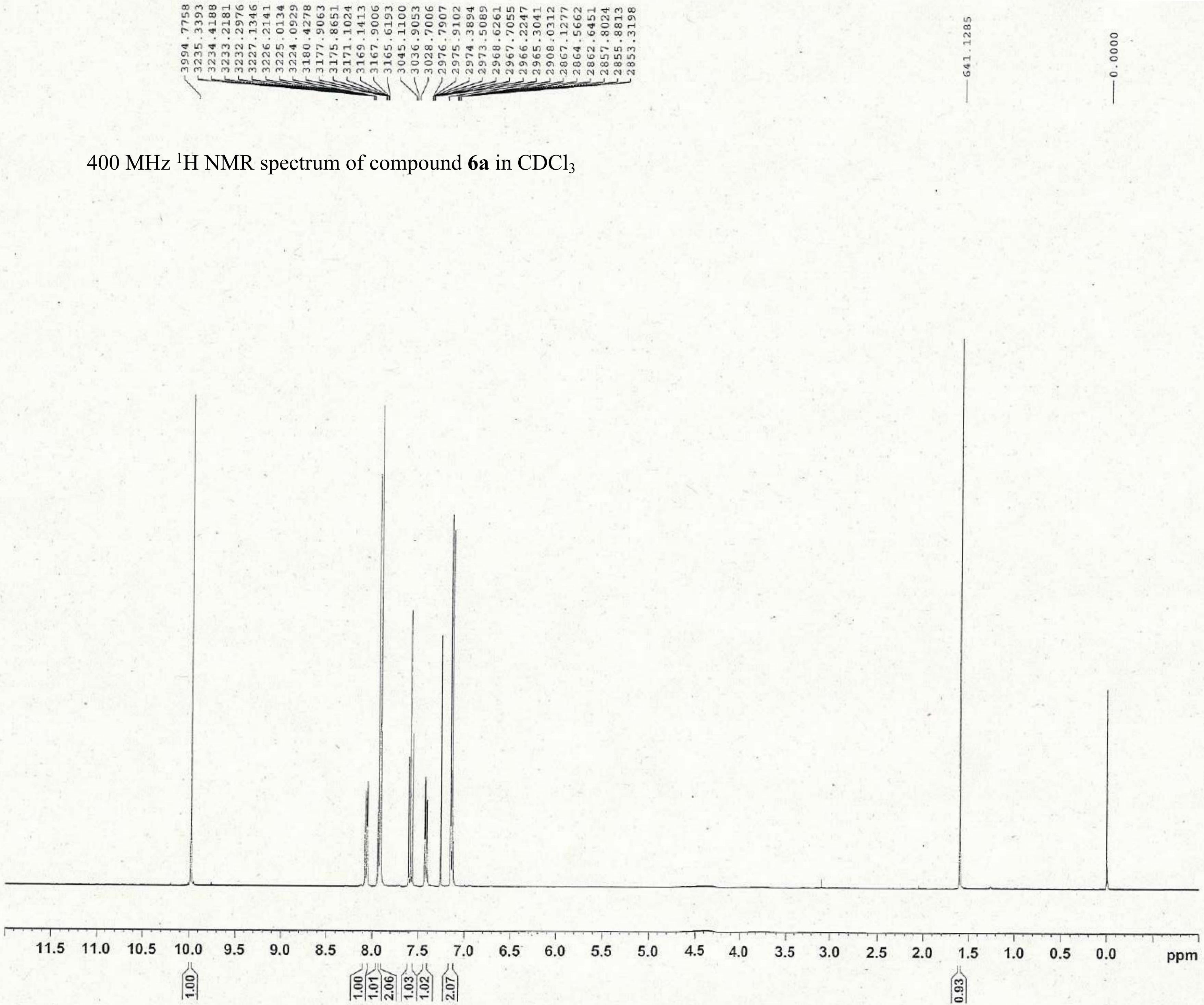

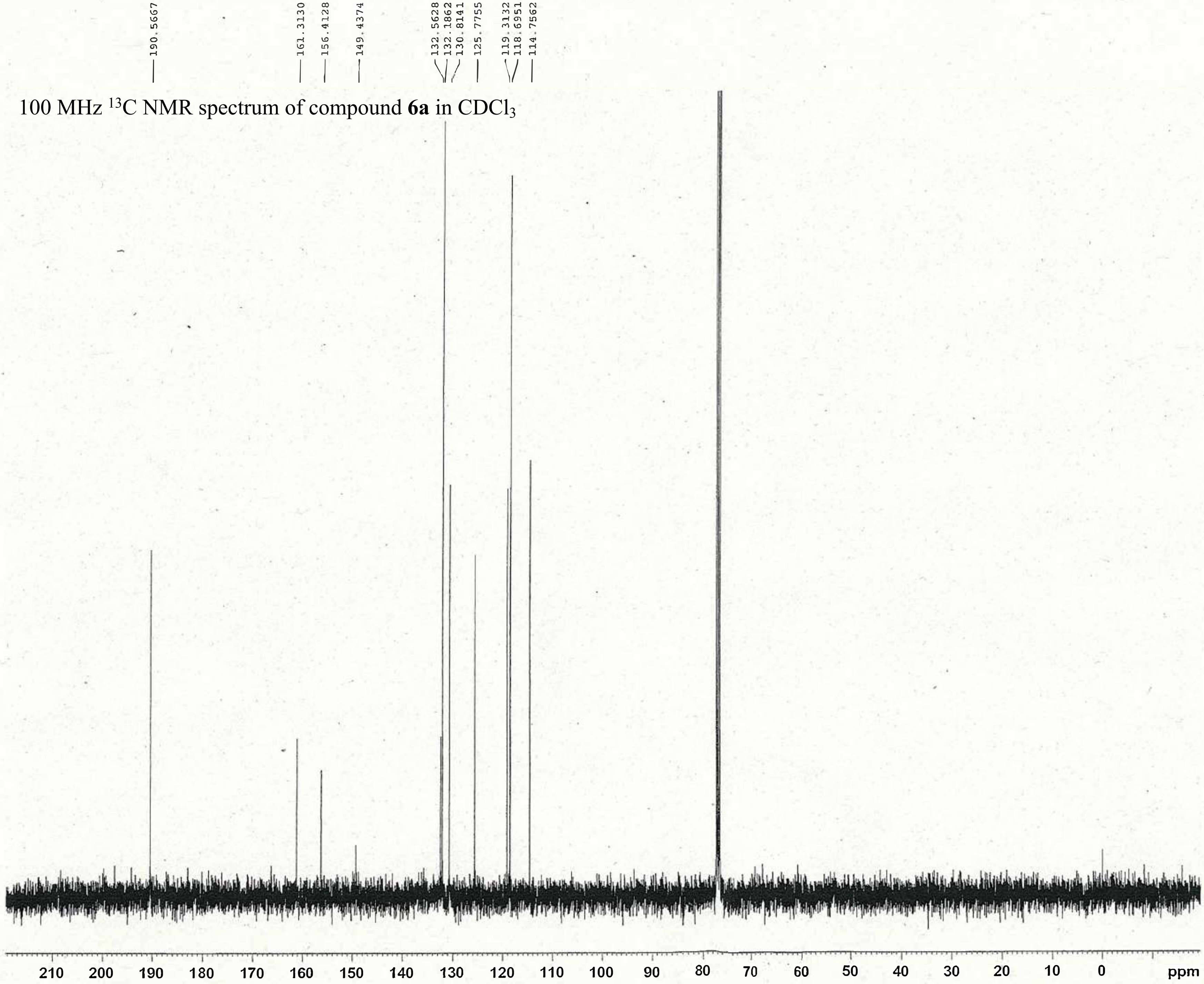

100 MHz ^{13}C NMR spectrum of compound **6a** in $CDCl_3$

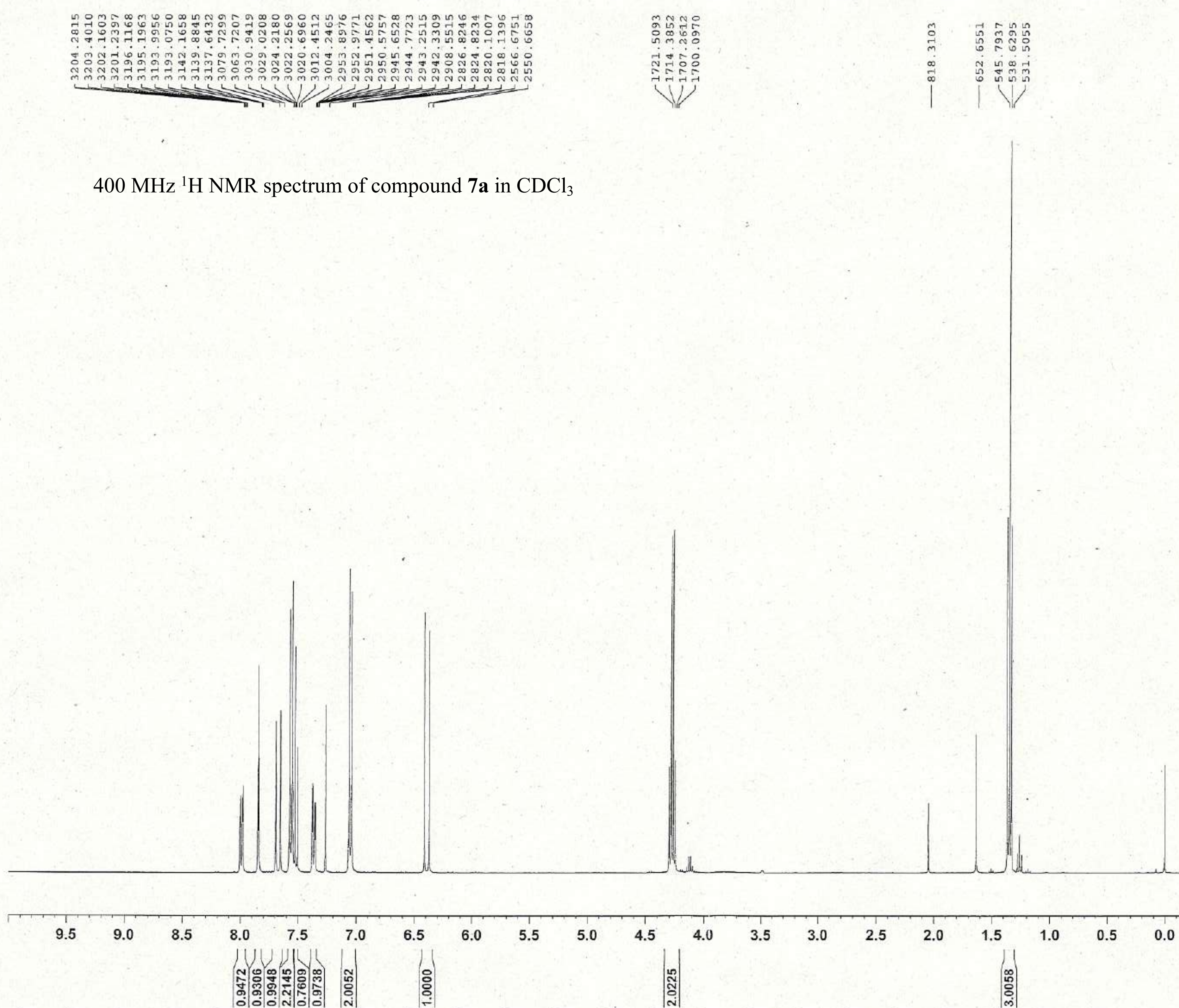

400 MHz ^{1}H NMR spectrum of compound **7a** in $CDCl_3$

100 MHz ^{13}C NMR spectrum of compound **7a** in $CDCl_3$

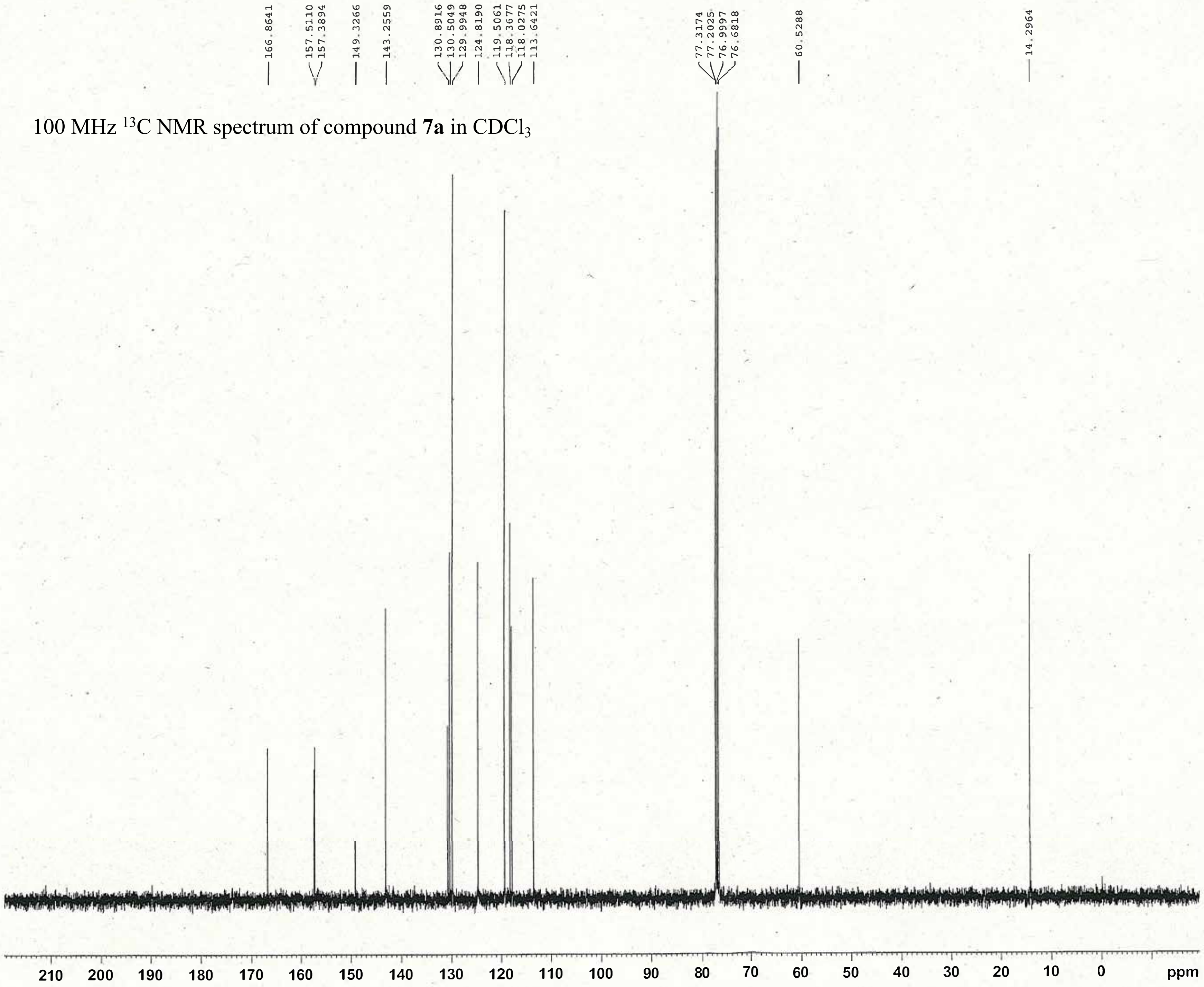

400 MHz ^{1}H NMR spectrum of compound **8a** in $CDCl_3$

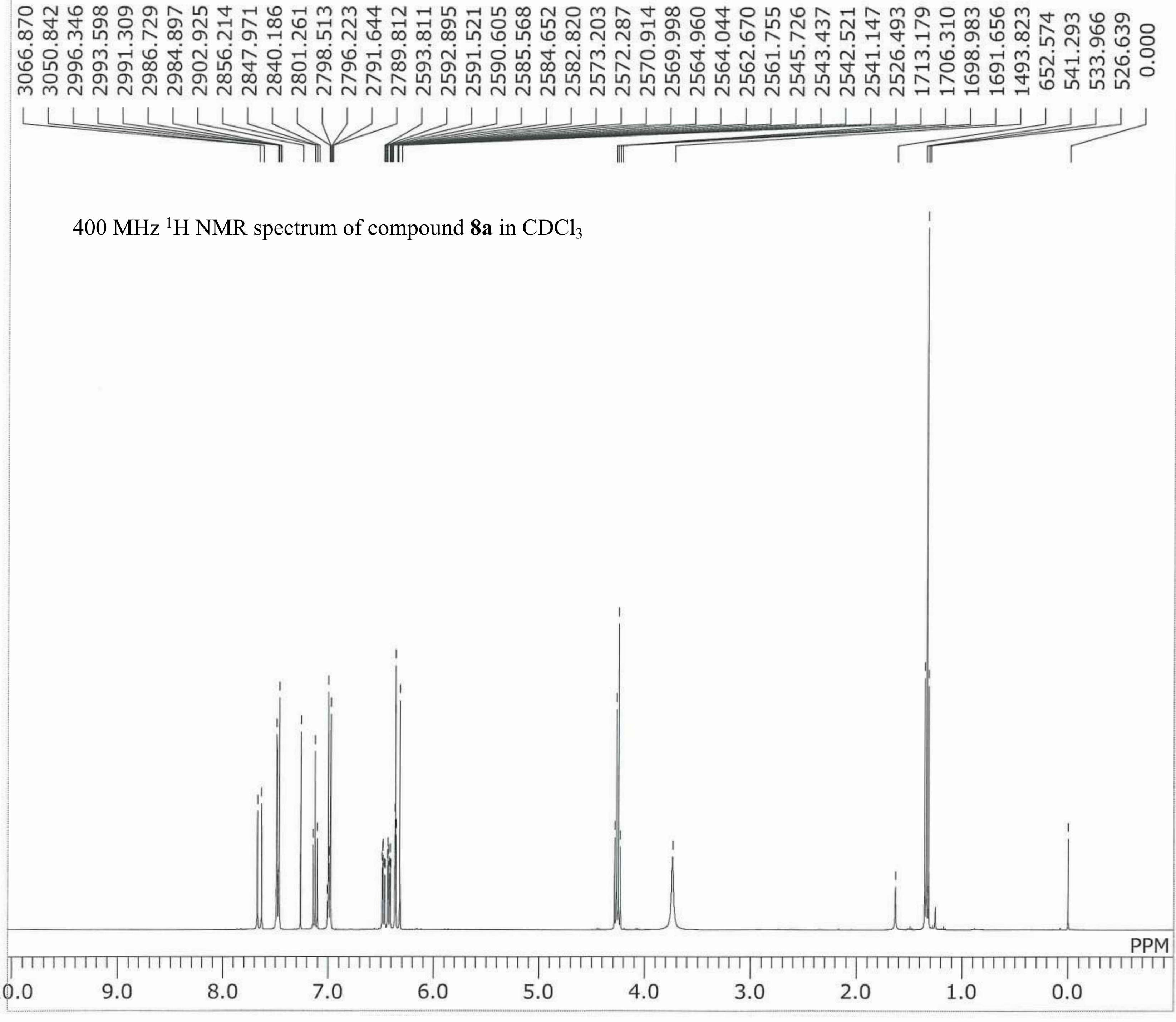

125 MHz ^{13}C NMR spectrum of compound **8a** in $CDCl_3$

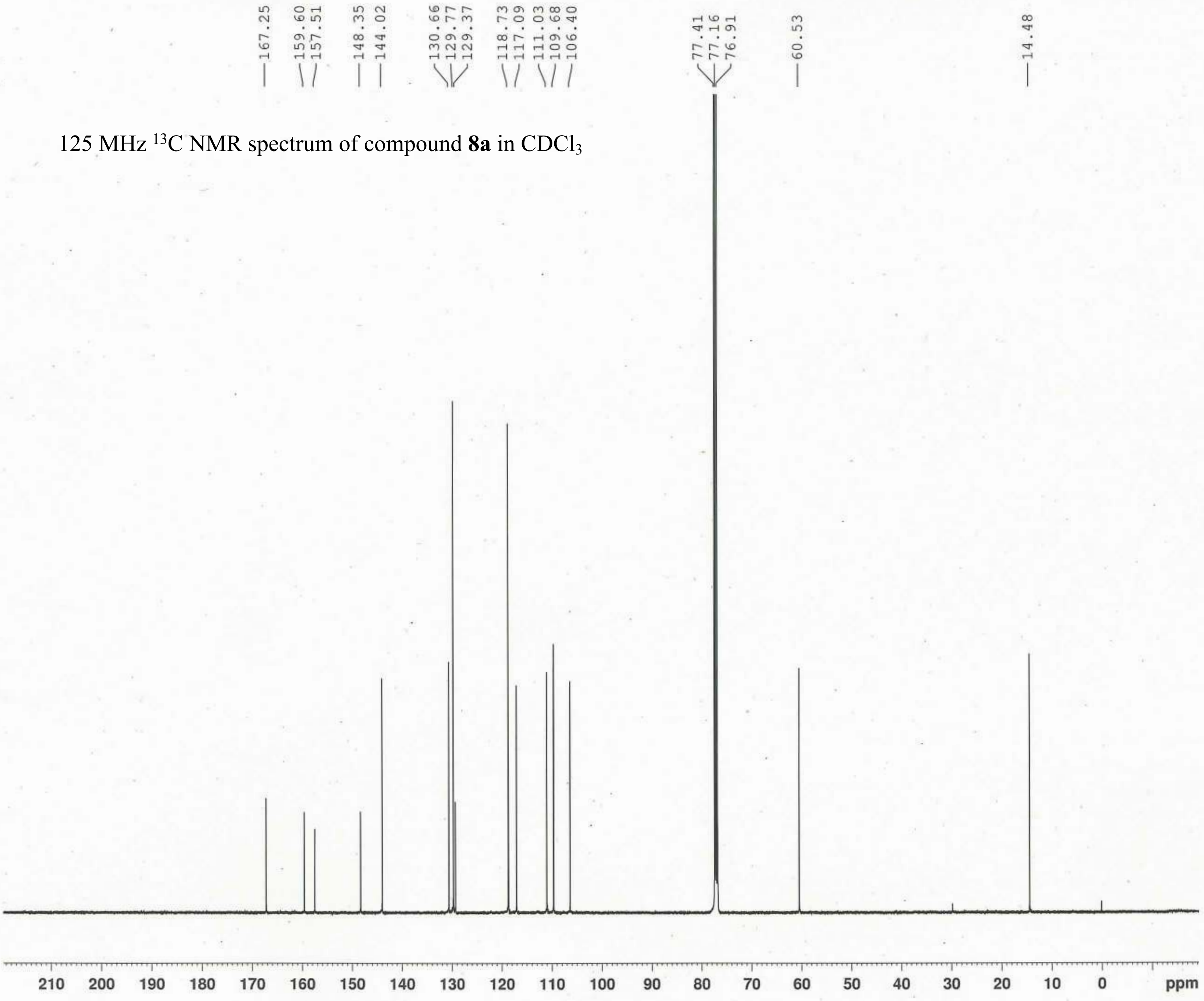

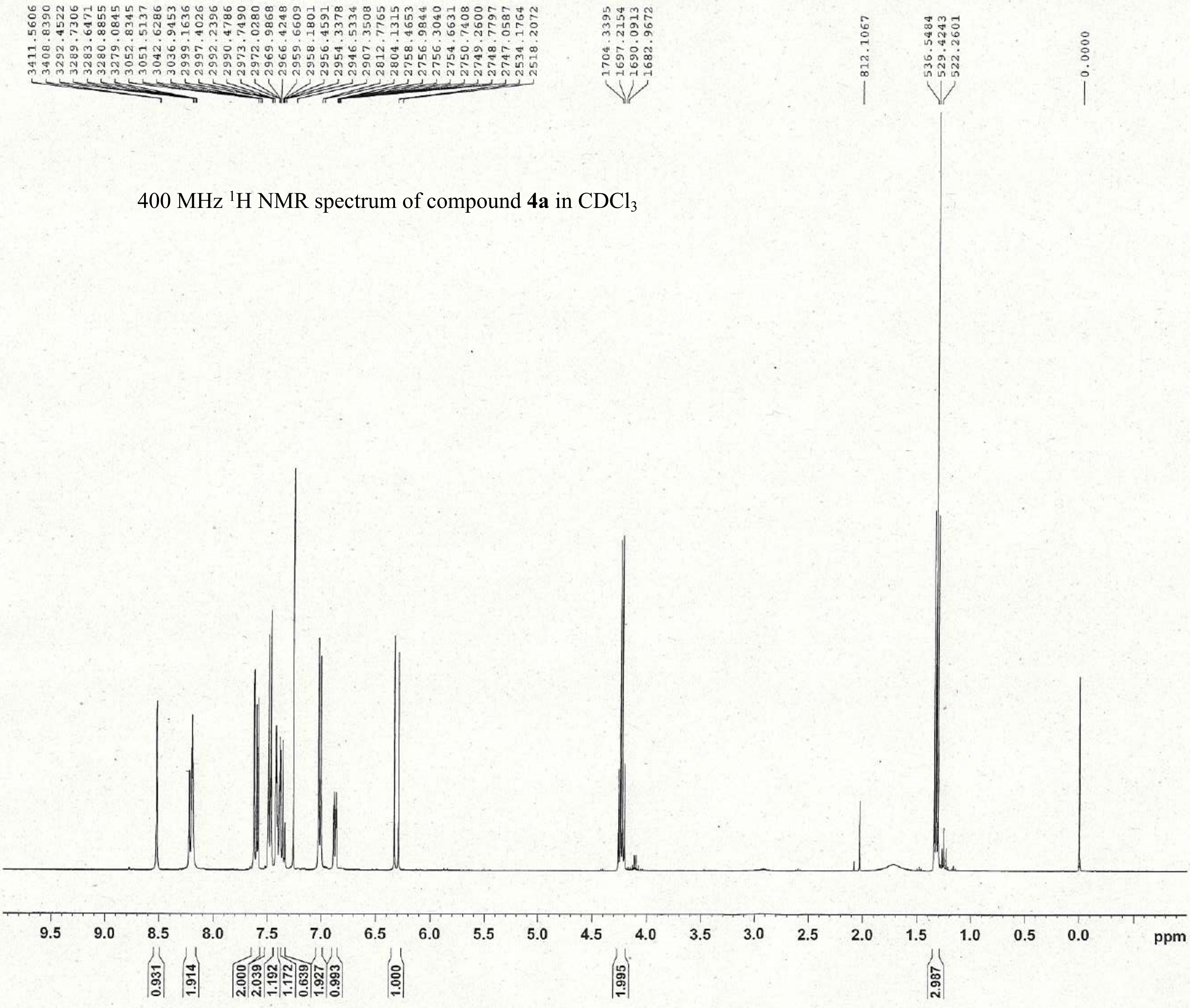

400 MHz ^{1}H NMR spectrum of compound **4a** in $CDCl_3$

100 MHz ^{13}C NMR spectrum of compound **4a** in $CDCl_3$

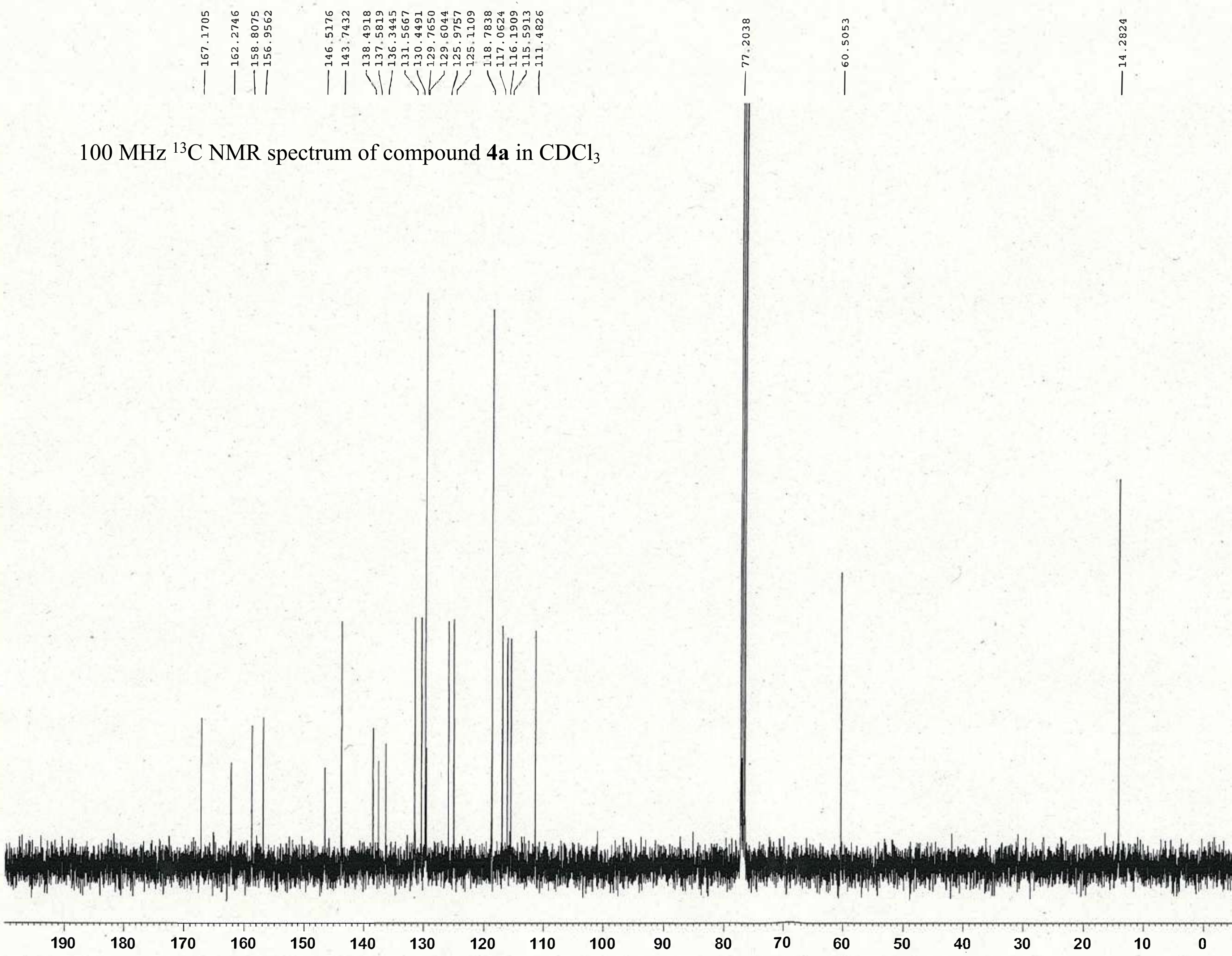

400 MHz ^{1}H NMR spectrum of compound **4b** in $CDCl_3$

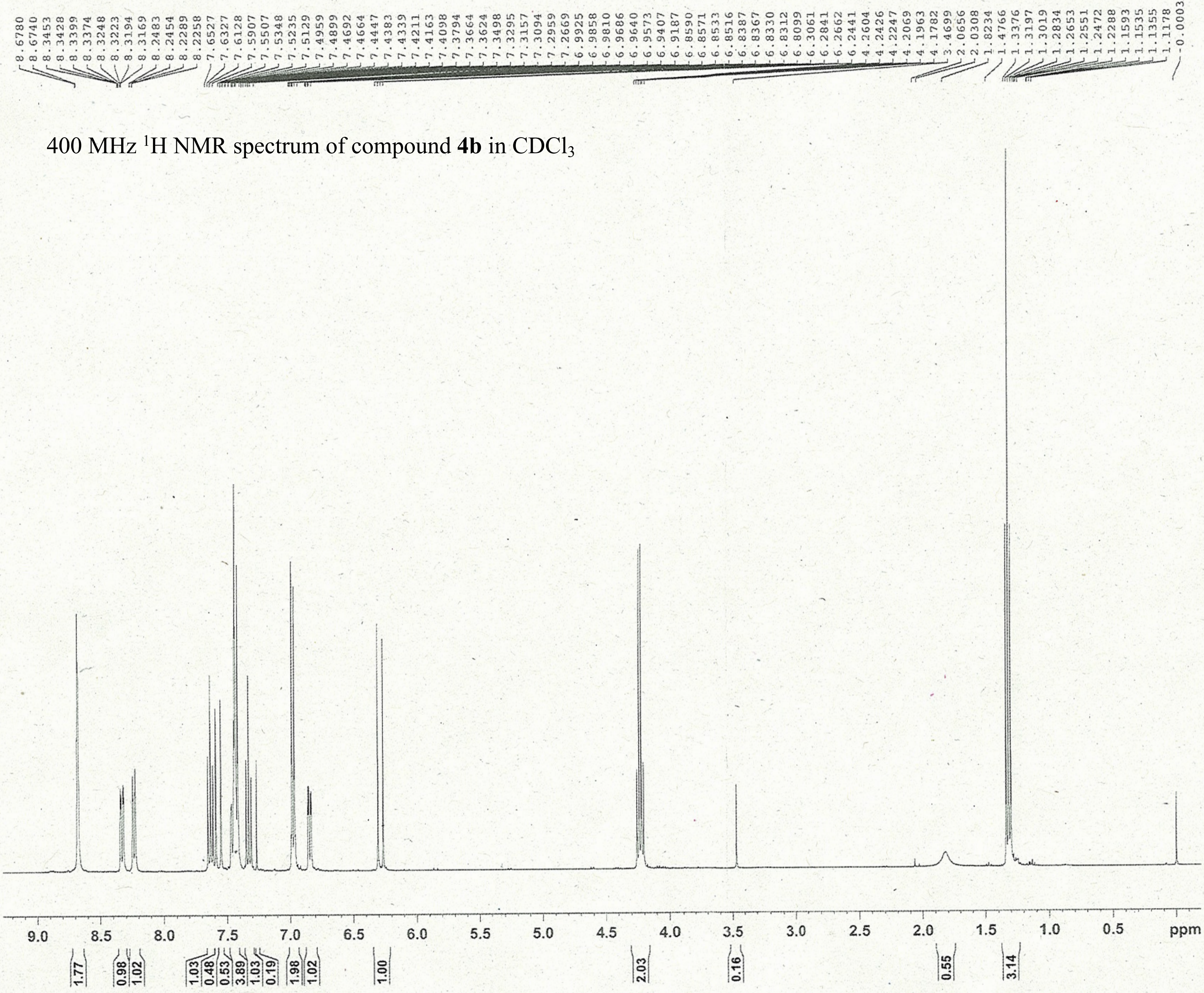

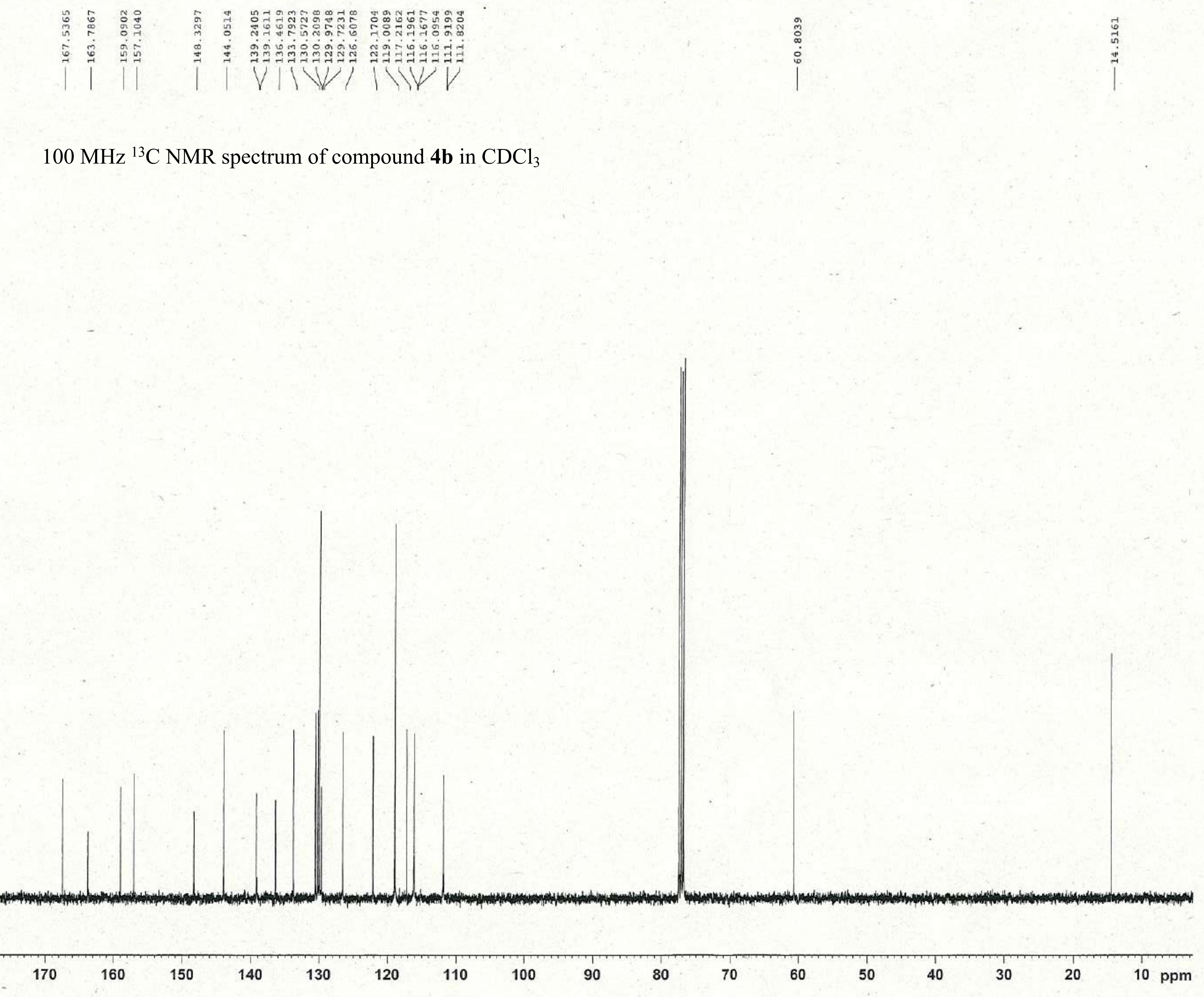

100 MHz ^{13}C NMR spectrum of compound **4b** in $CDCl_3$

400 MHz ^1H NMR Spectrum of compound **9** in $CDCl_3$

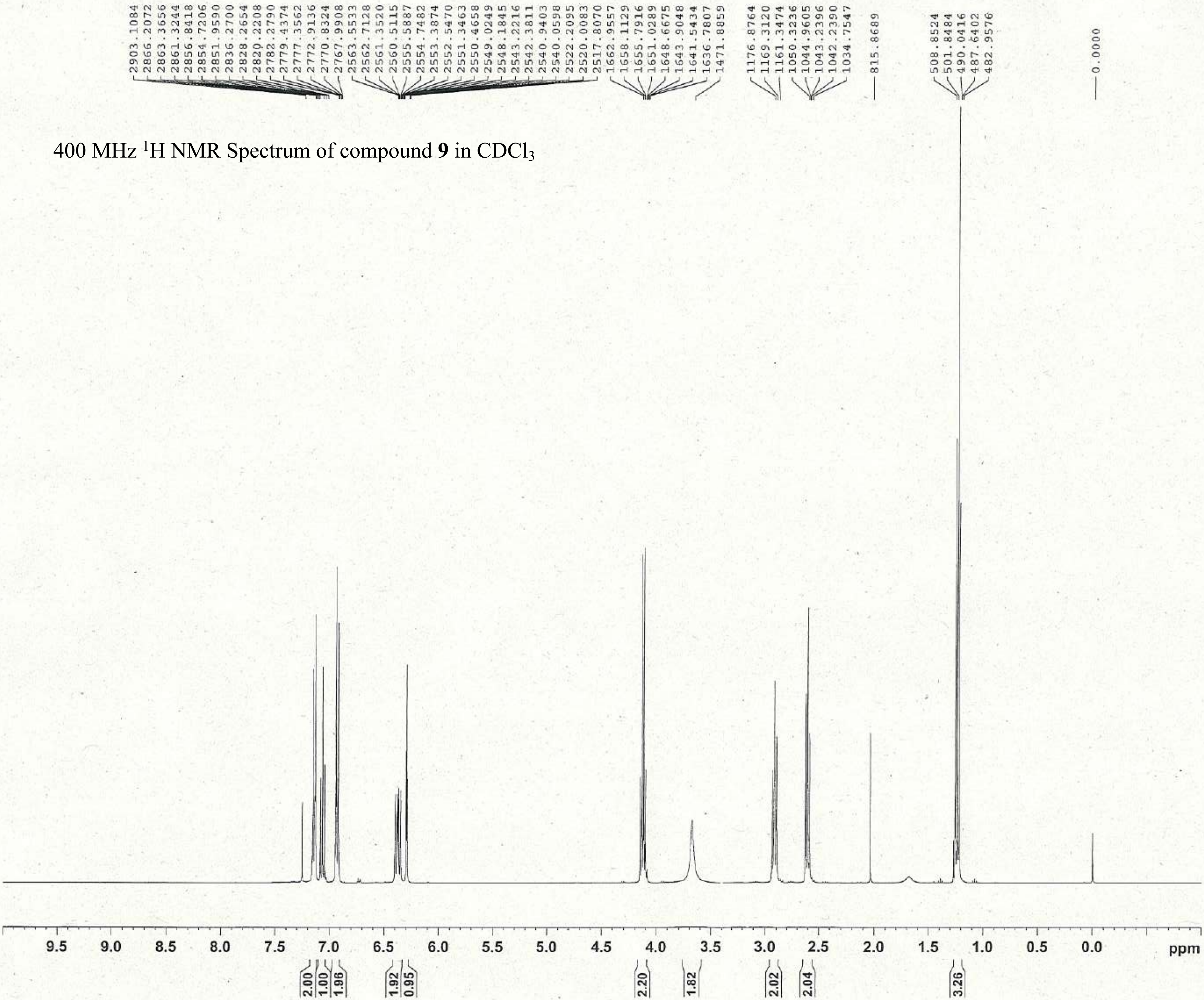

100 MHz ^{13}C NMR spectrum of compound **9** in $CDCl_3$

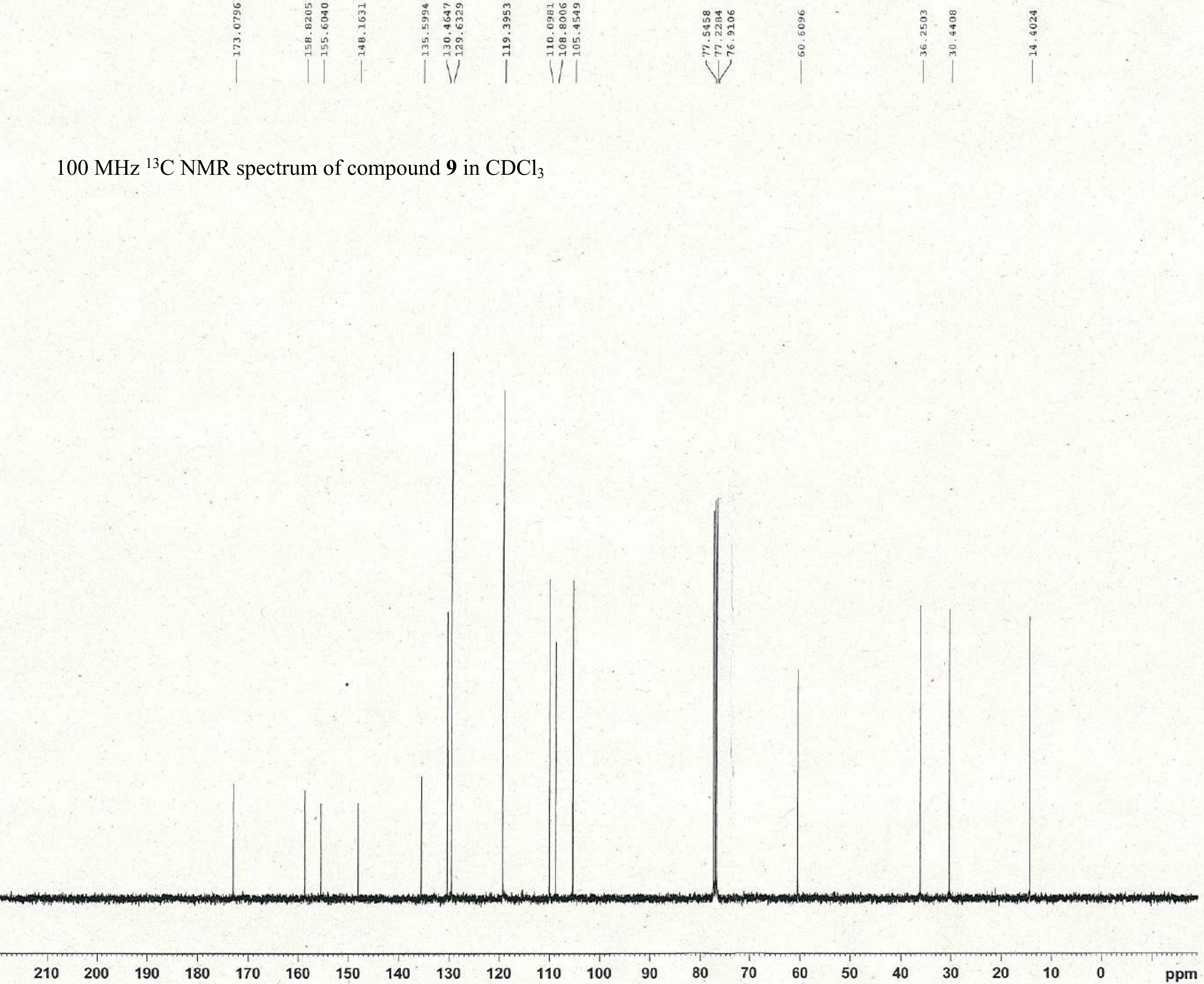

400 MHz ^{1}H NMR Spectrum of compound **4e** in $CDCl_3$

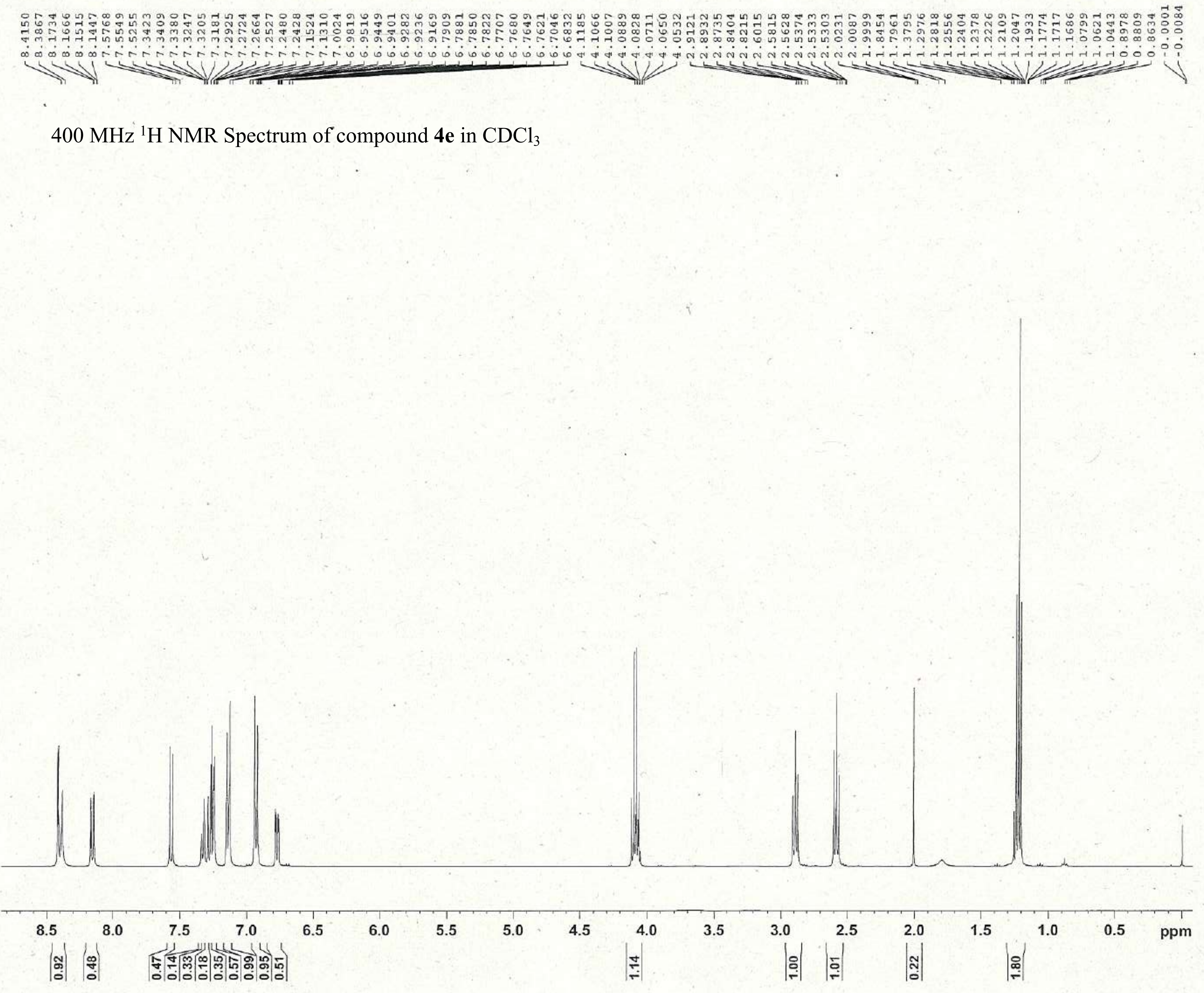

100 MHz ^{13}C NMR spectrum of compound **4e** in $CDCl_3$

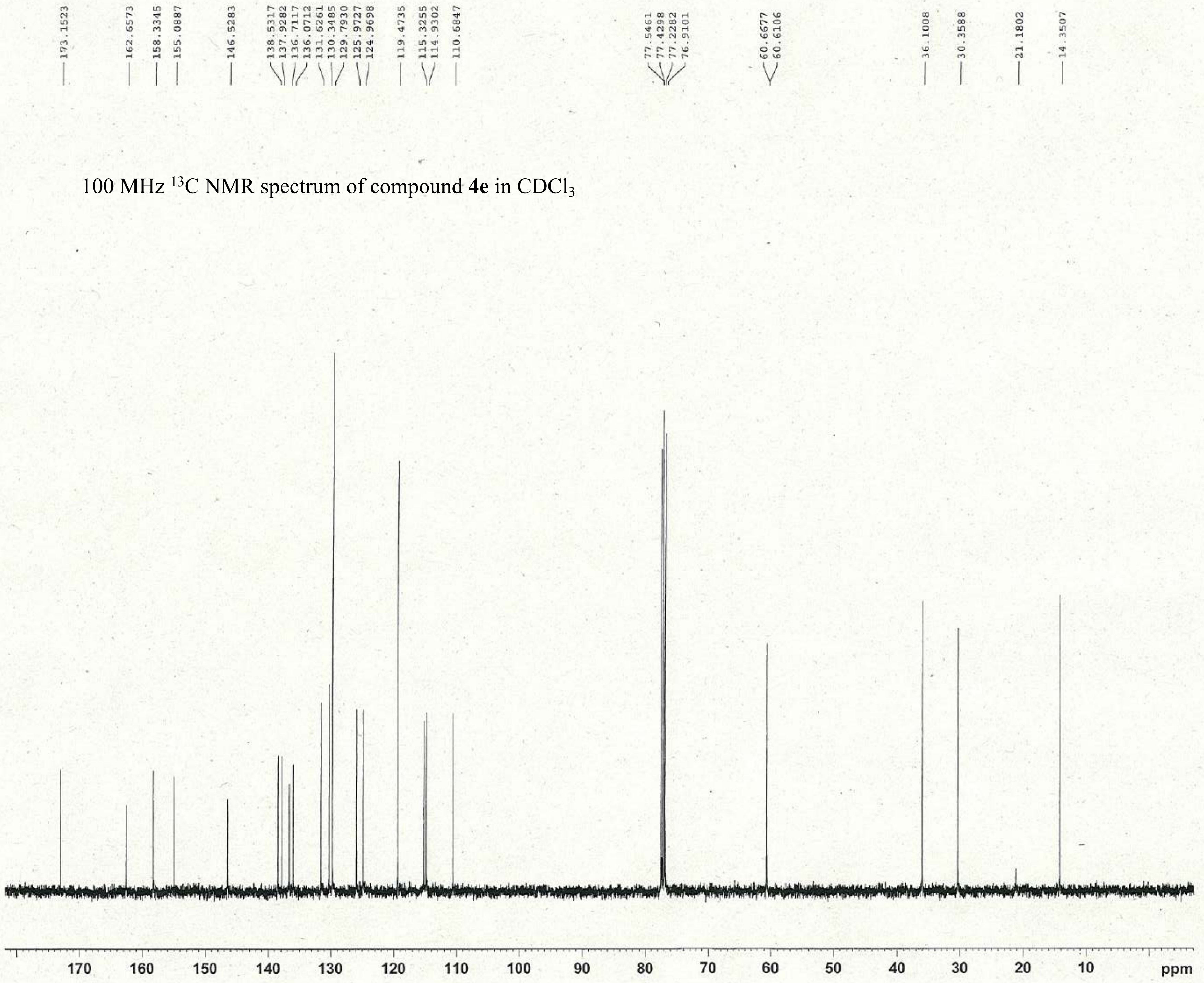

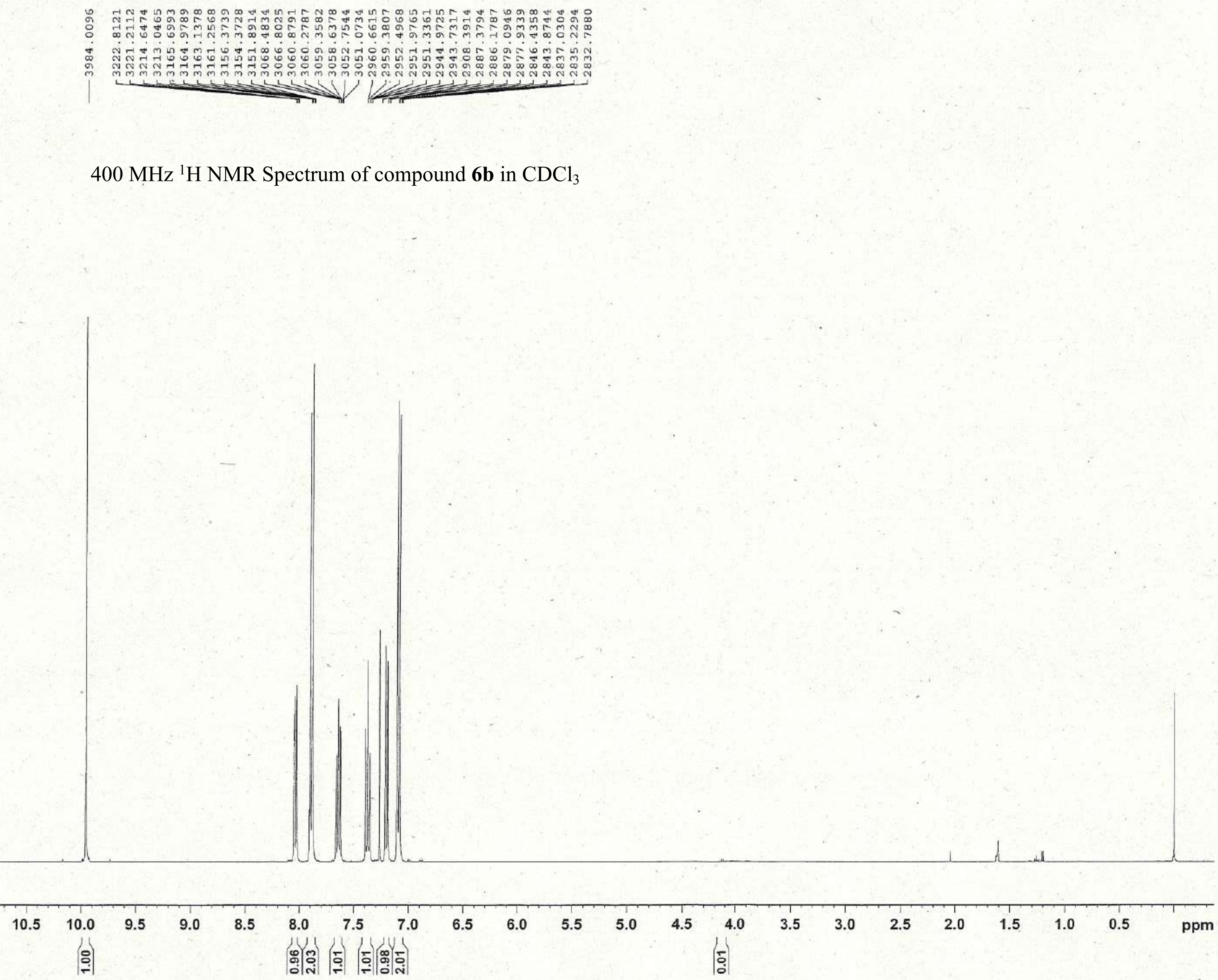

400 MHz ^{1}H NMR Spectrum of compound **6b** in $CDCl_3$

100 MHz ^{13}C NMR spectrum of compound **6b** in $CDCl_3$

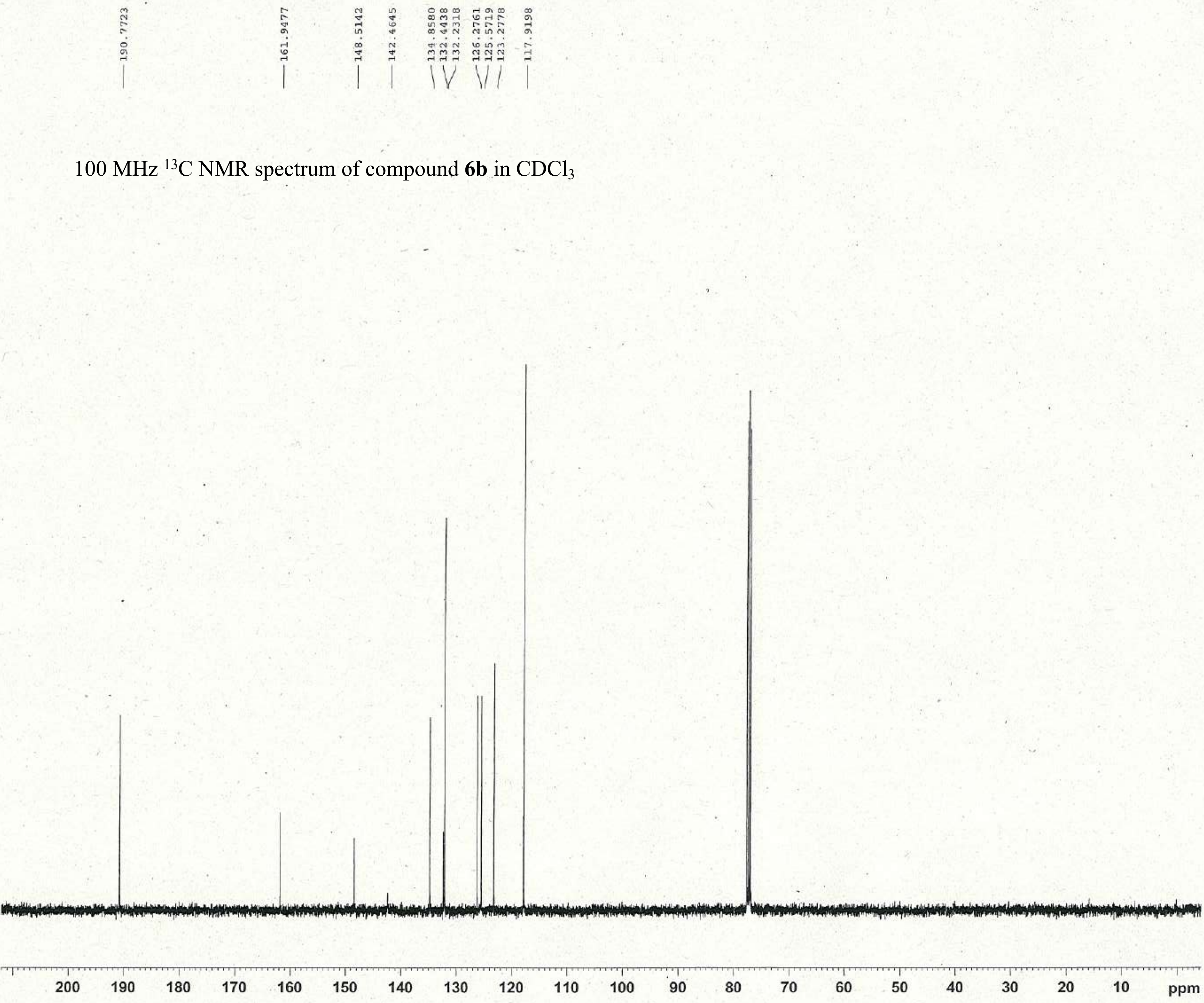

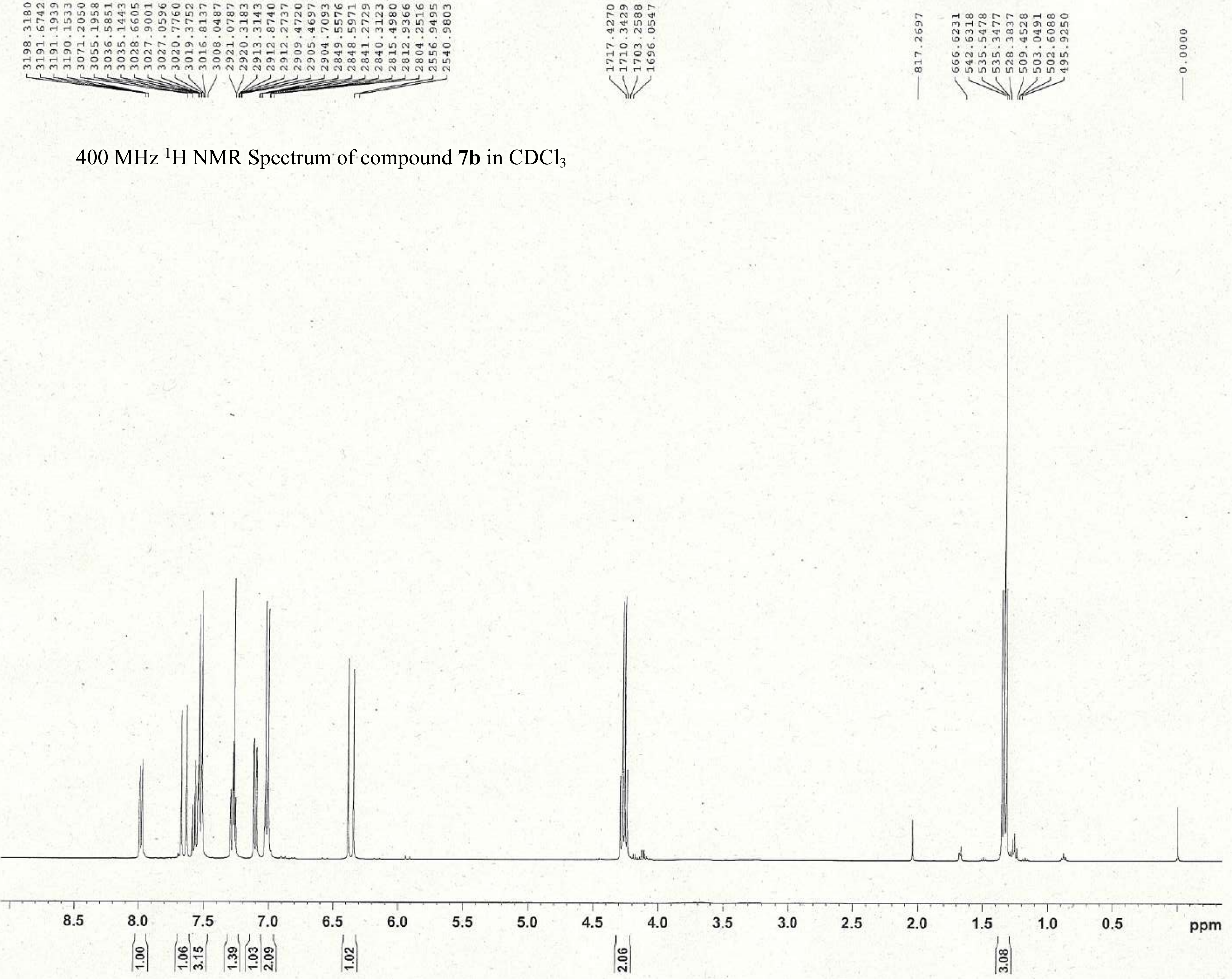

400 MHz ^{1}H NMR Spectrum of compound **7b** in $CDCl_3$

100 MHz ^{13}C NMR spectrum of compound **7b** in $CDCl_3$

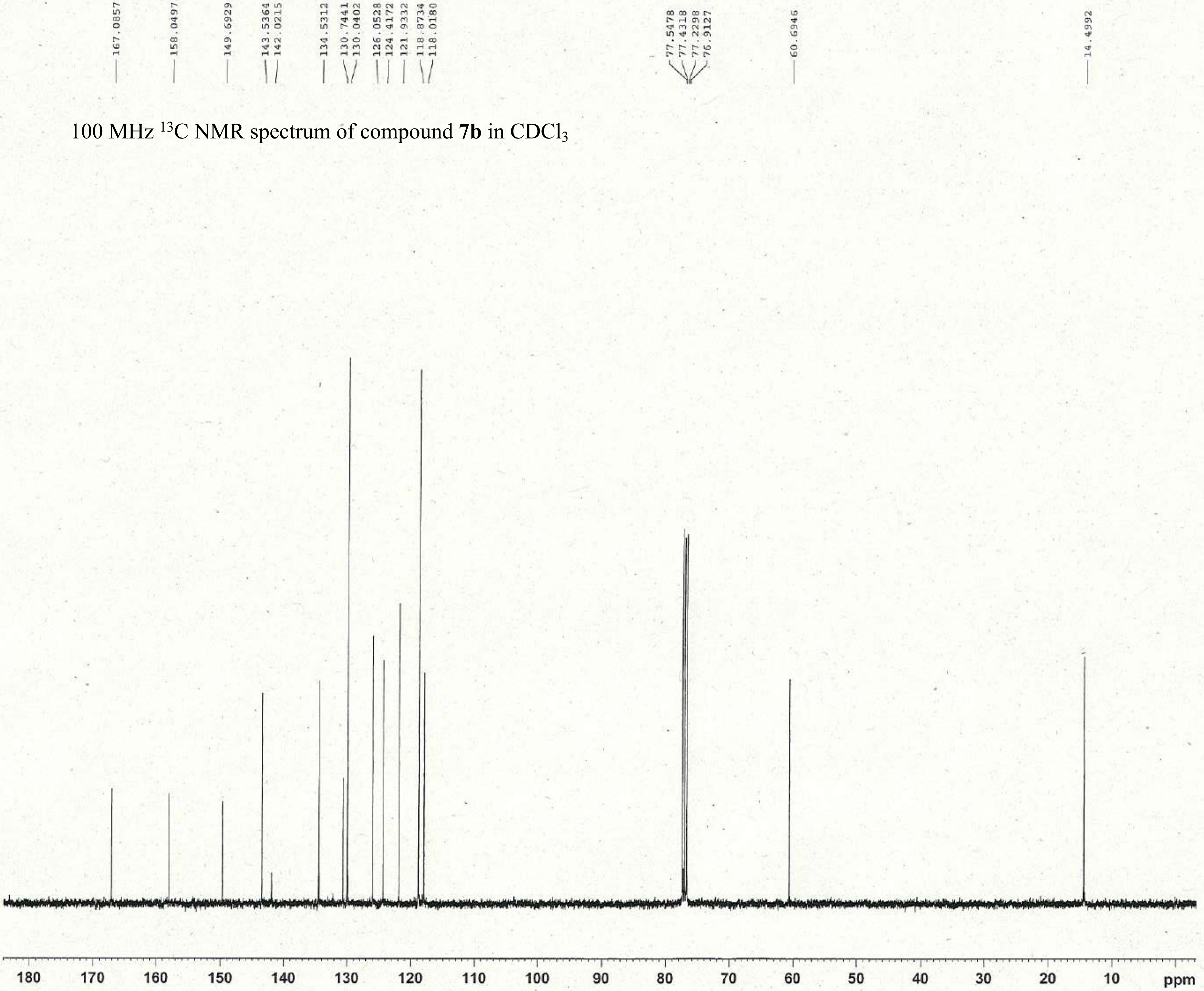

400 MHz ^{1}H NMR Spectrum of compound **8b** in $CDCl_3$

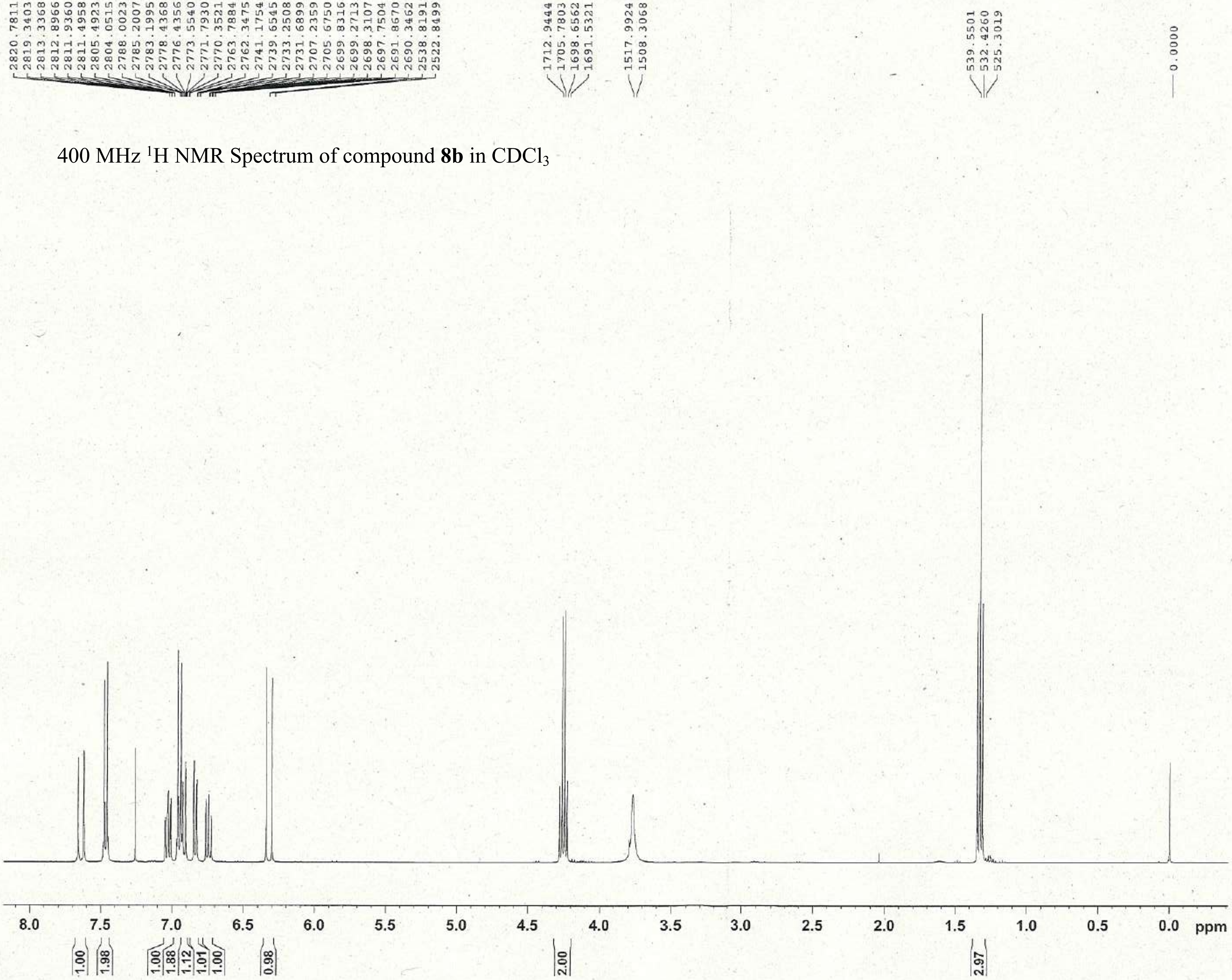

100 MHz ^{13}C NMR spectrum of compound **8b** in $CDCl_3$

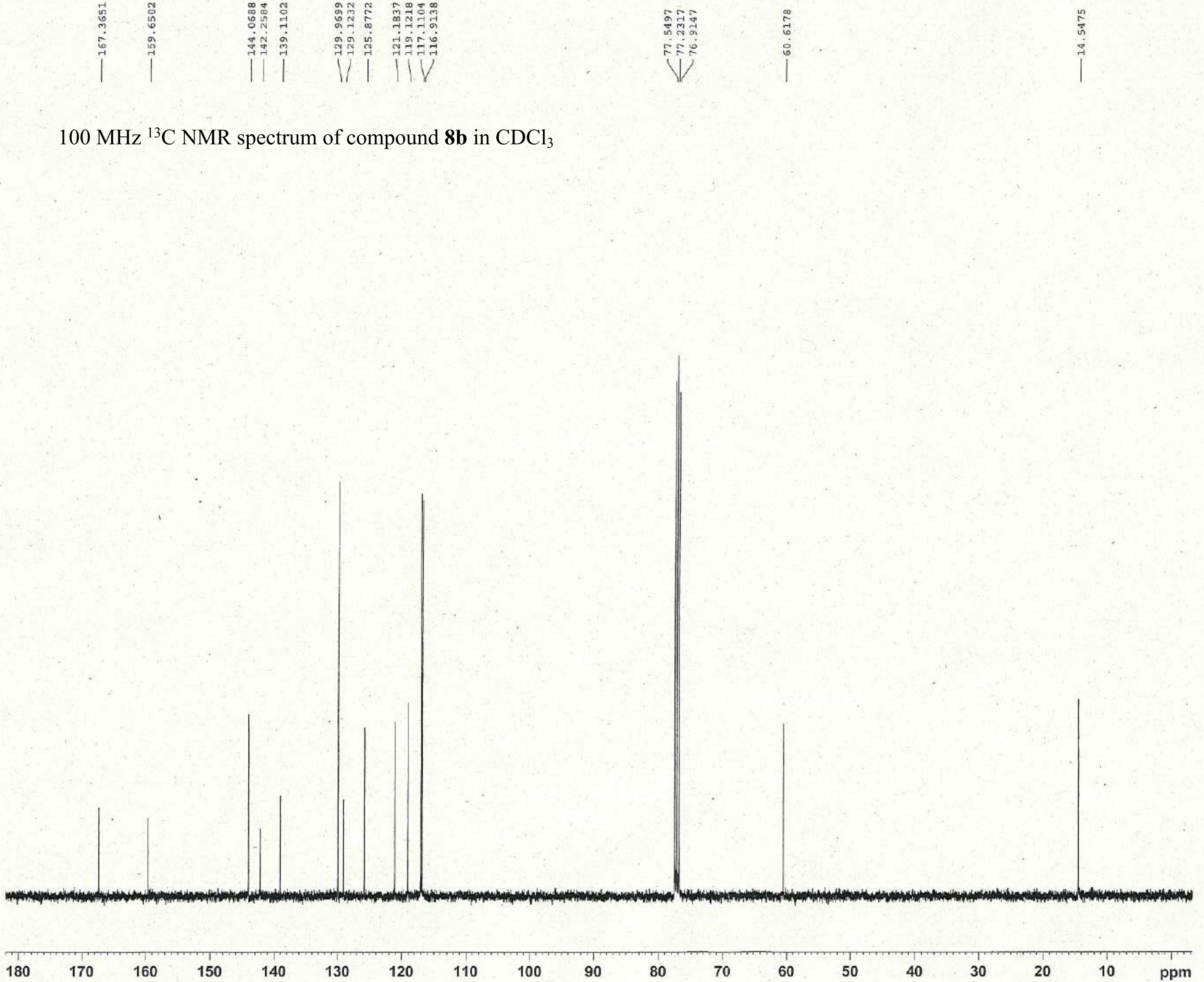

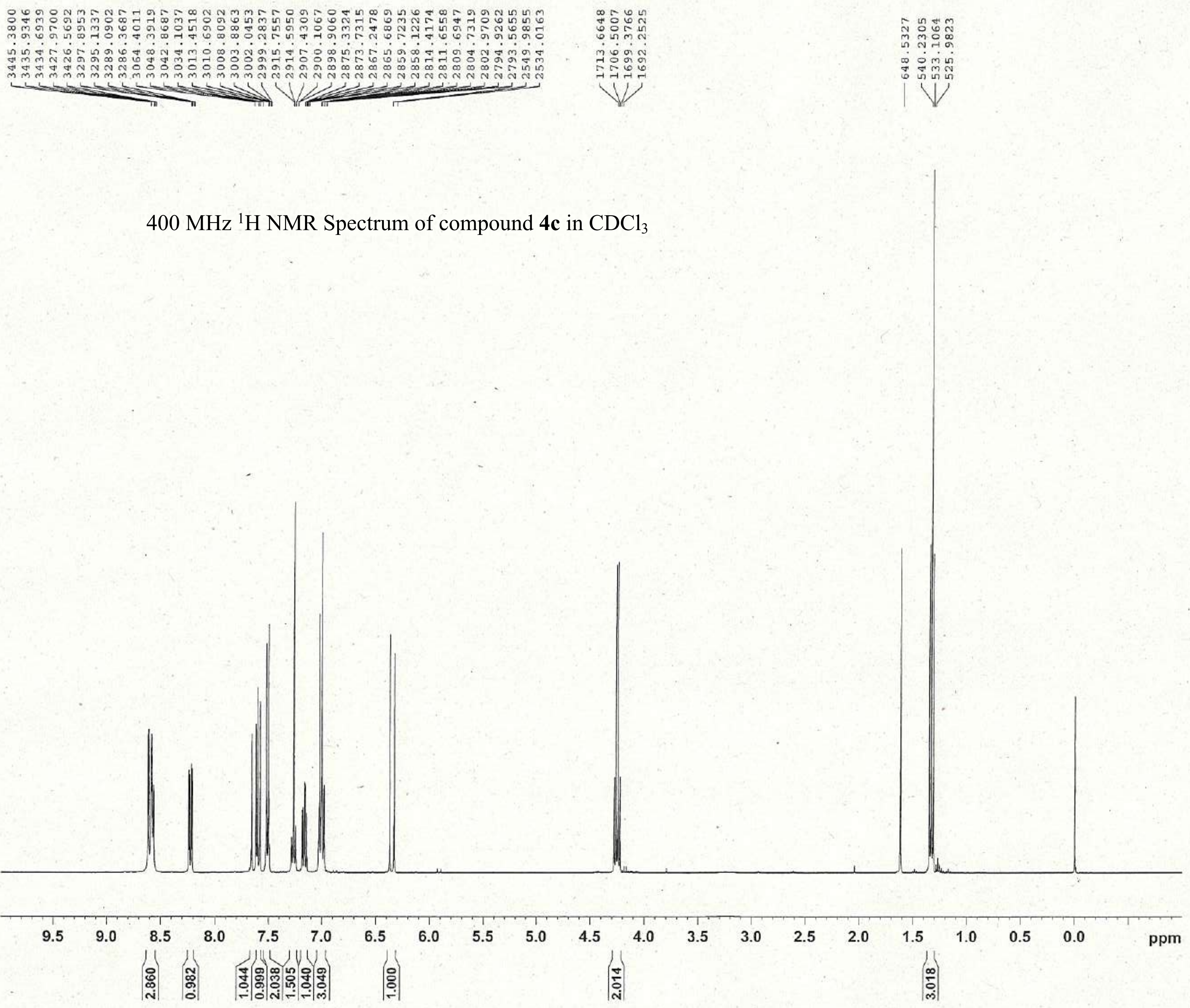

400 MHz ^{1}H NMR Spectrum of compound **4c** in $CDCl_3$

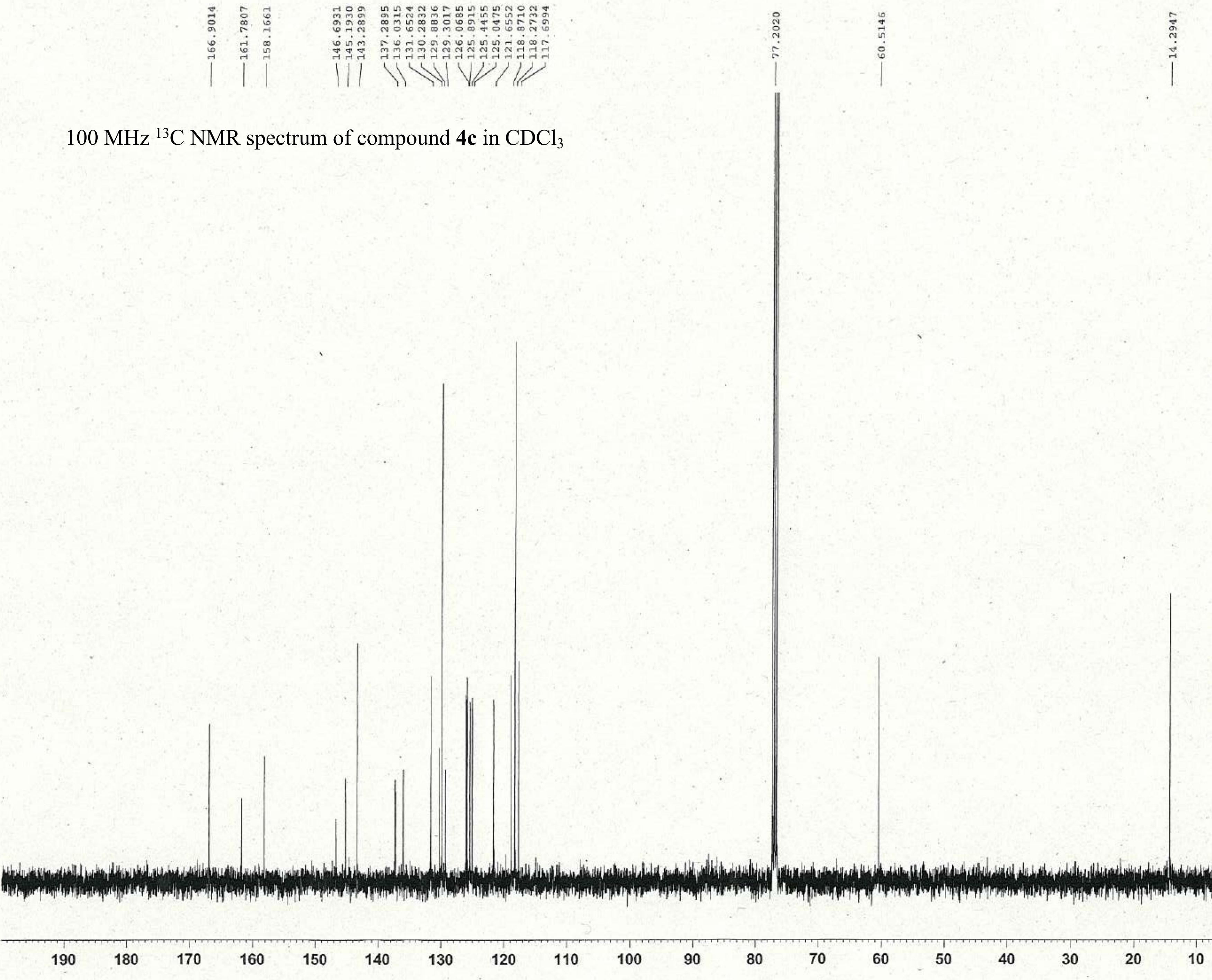

100 MHz ^{13}C NMR spectrum of compound **4c** in $CDCl_3$

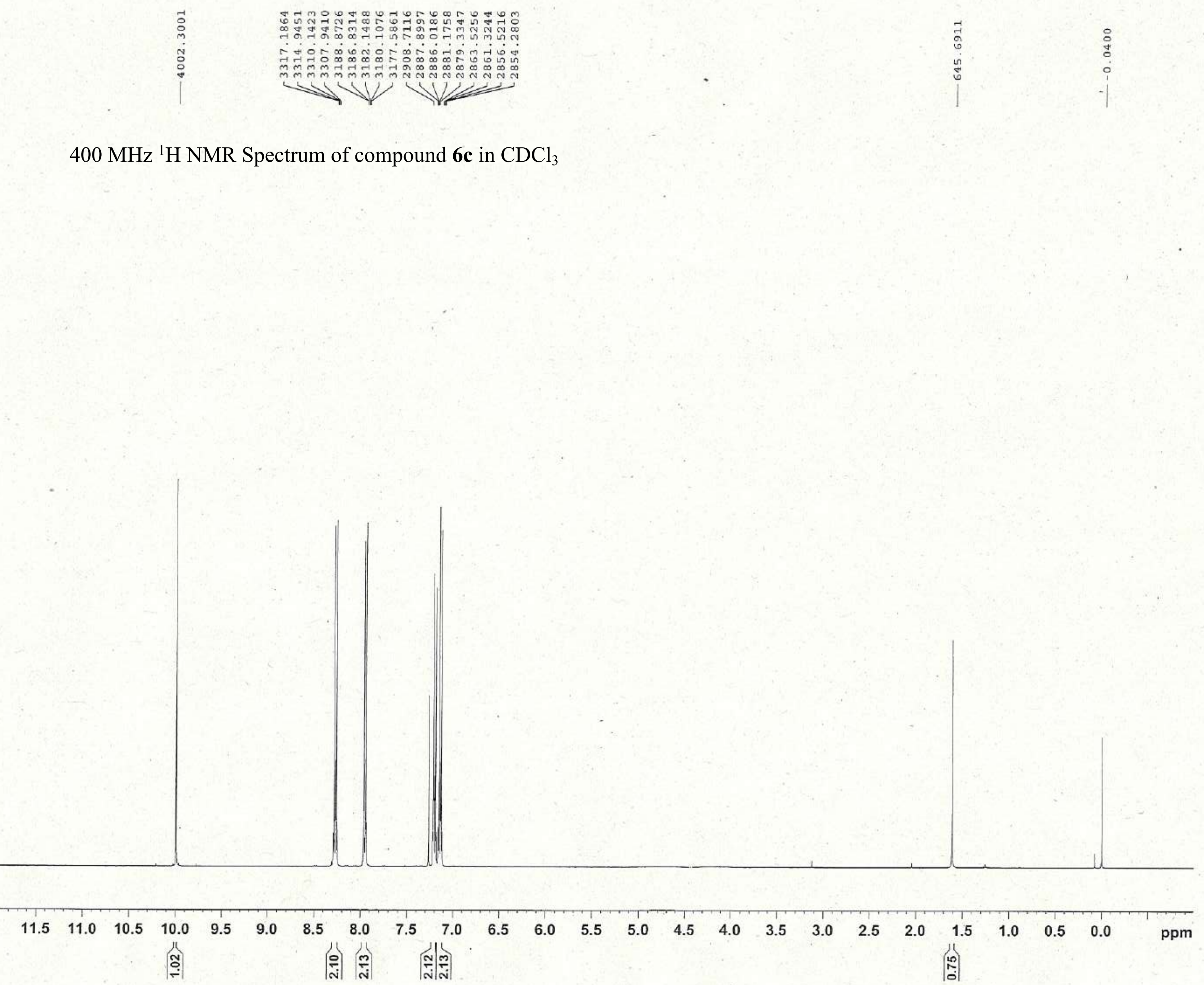

400 MHz ^{1}H NMR Spectrum of compound **6c** in $CDCl_3$

100 MHz ^{13}C NMR Spectrum of compound **6c** in $CDCl_3$

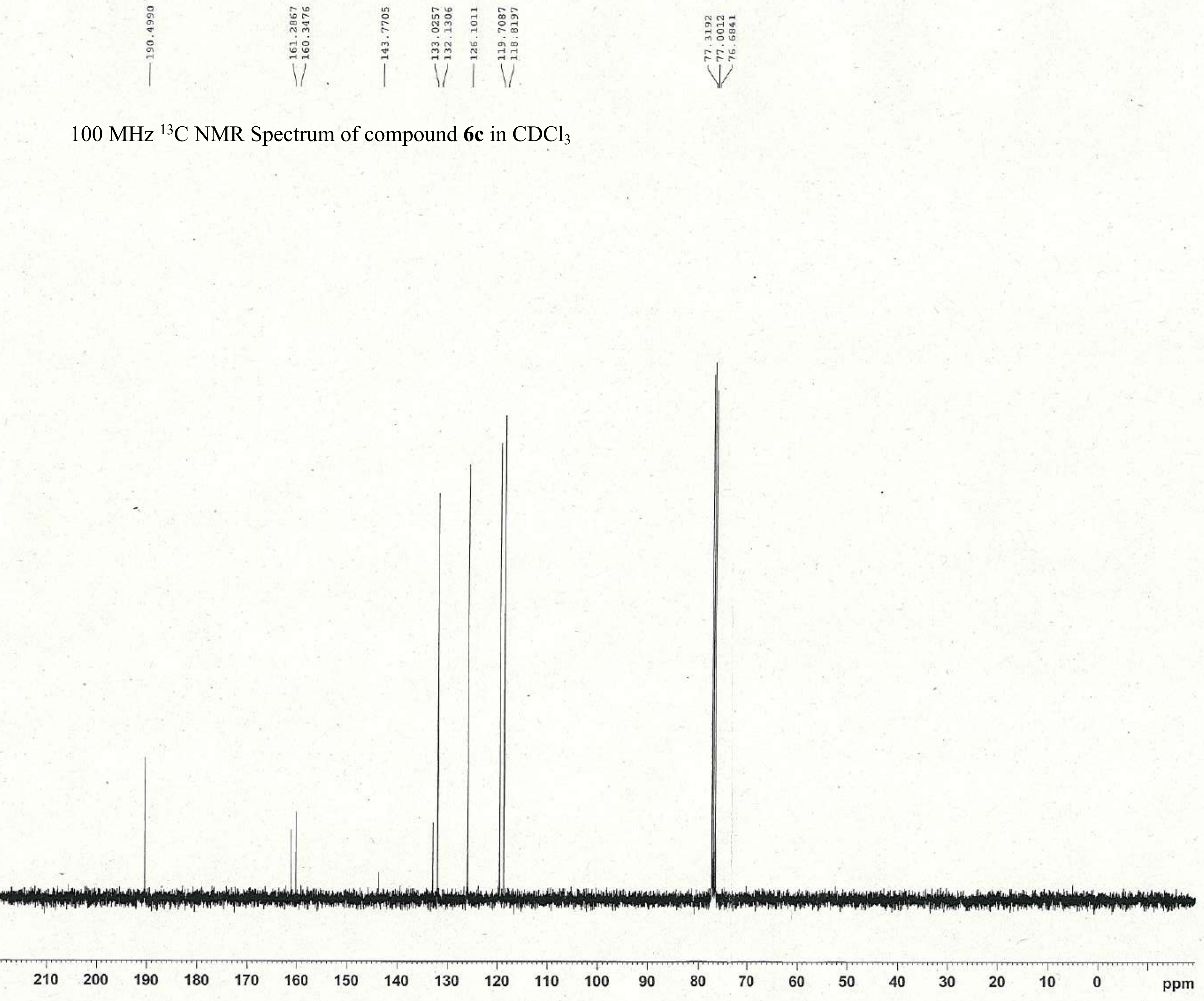

400 MHz ^{1}H NMR Spectrum of compound **7c** in $CDCl_3$

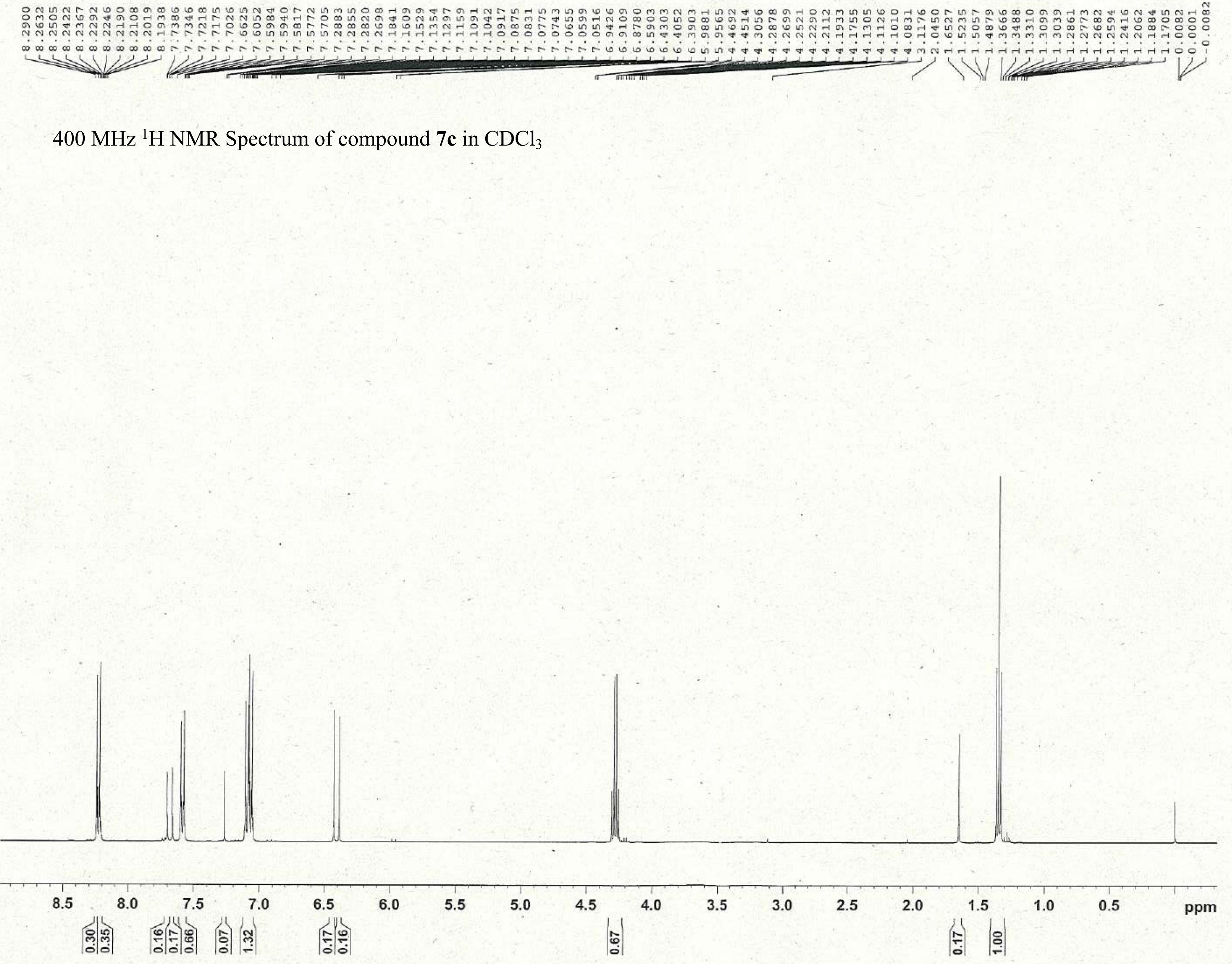

100 MHz ^{13}C NMR Spectrum of compound **7c** in $CDCl_3$

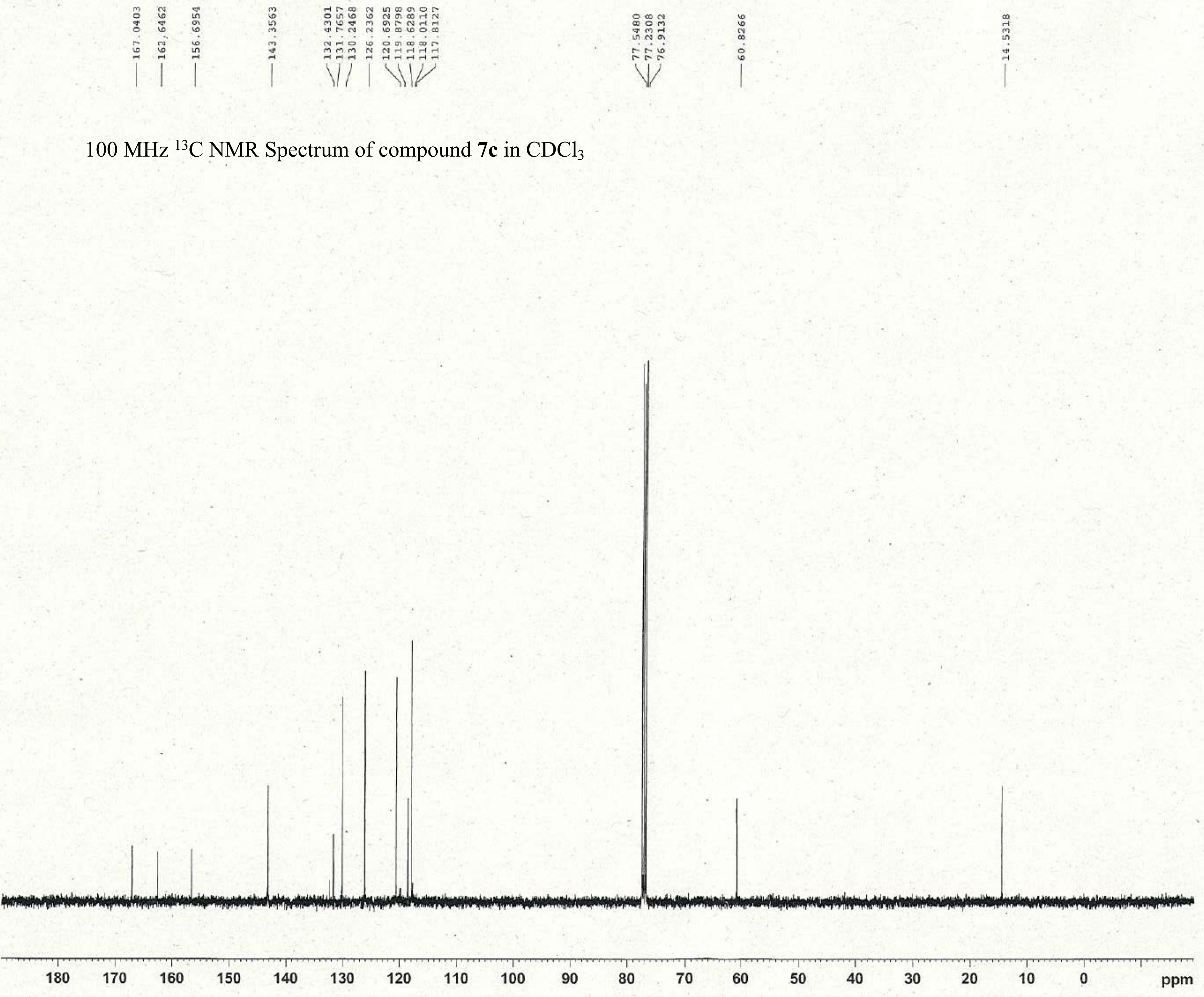

400 MHz ^1H NMR Spectrum of compound **8c** in $CDCl_3$

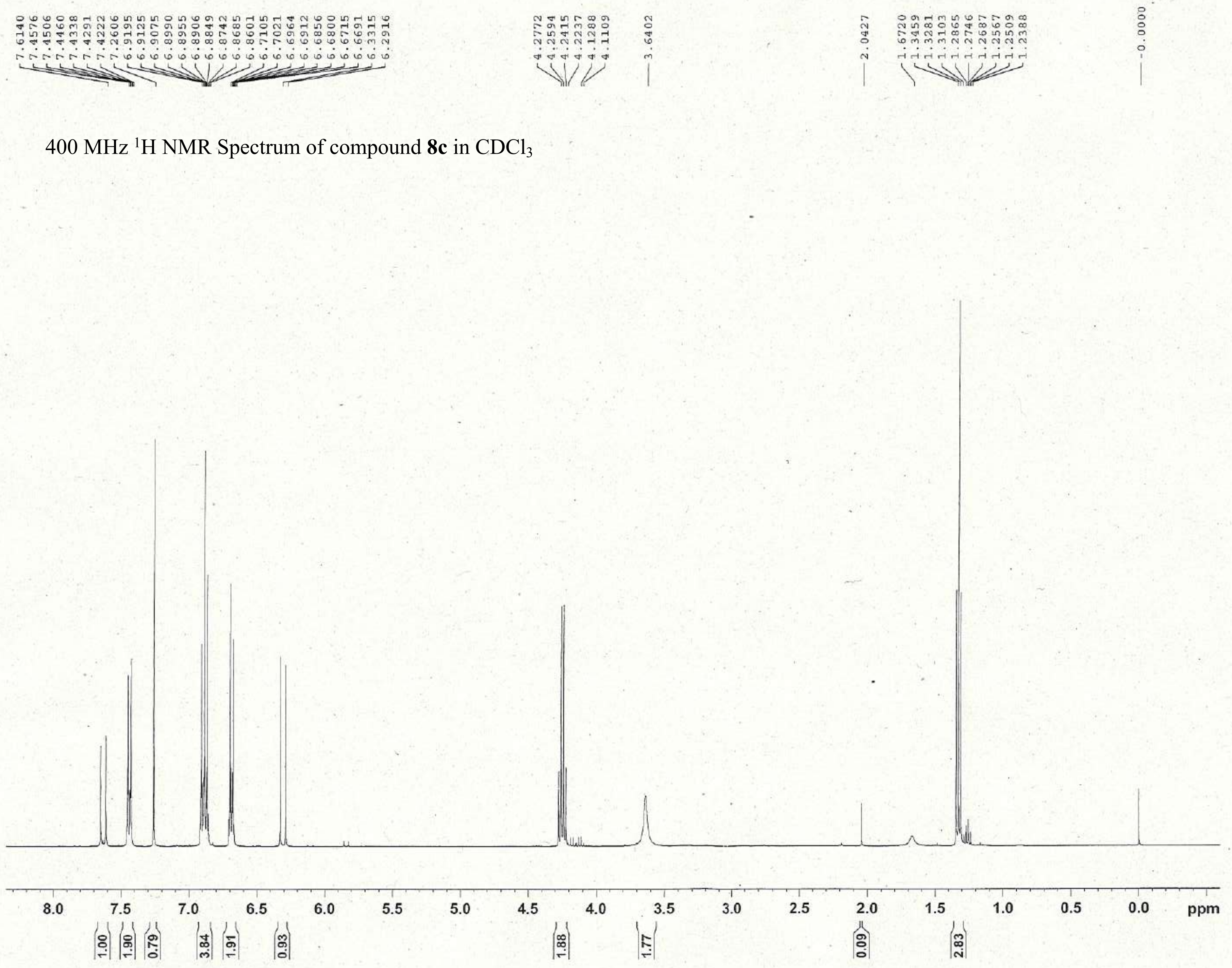

100 MHz ^{13}C NMR Spectrum of compound **8c** in $CDCl_3$

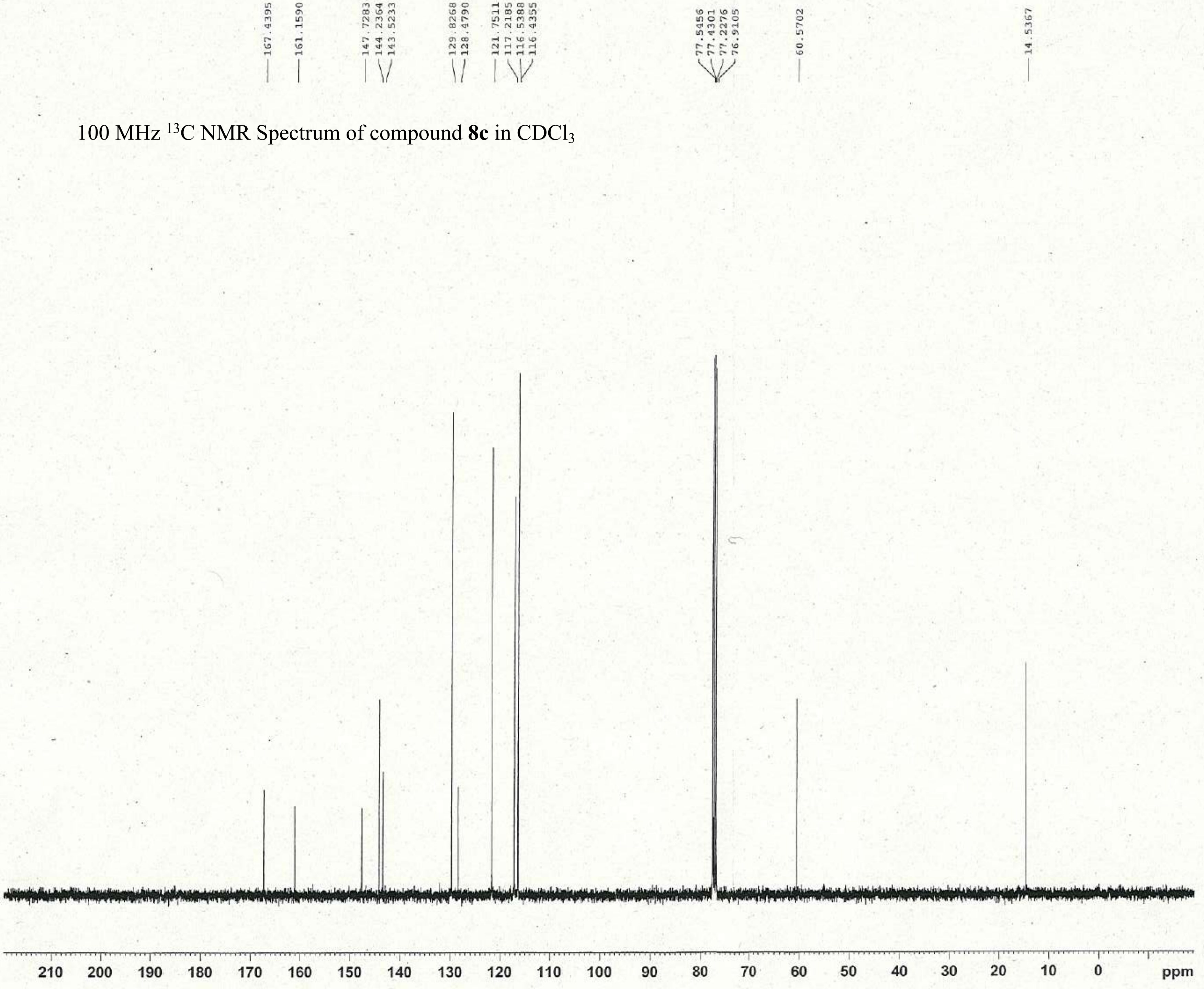

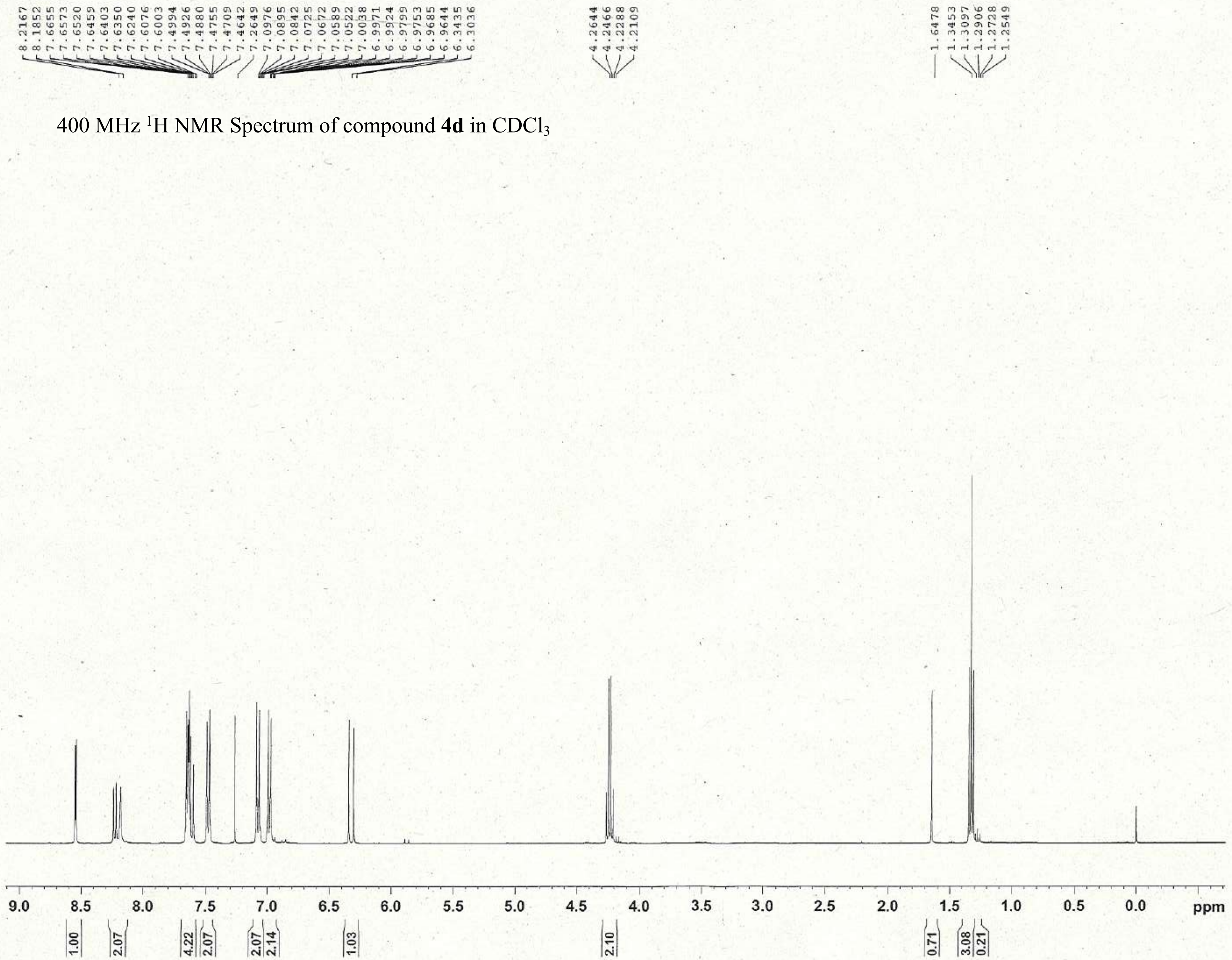

400 MHz ^{1}H NMR Spectrum of compound **4d** in $CDCl_3$

100 MHz ^{13}C NMR Spectrum of compound **4d** in $CDCl_3$

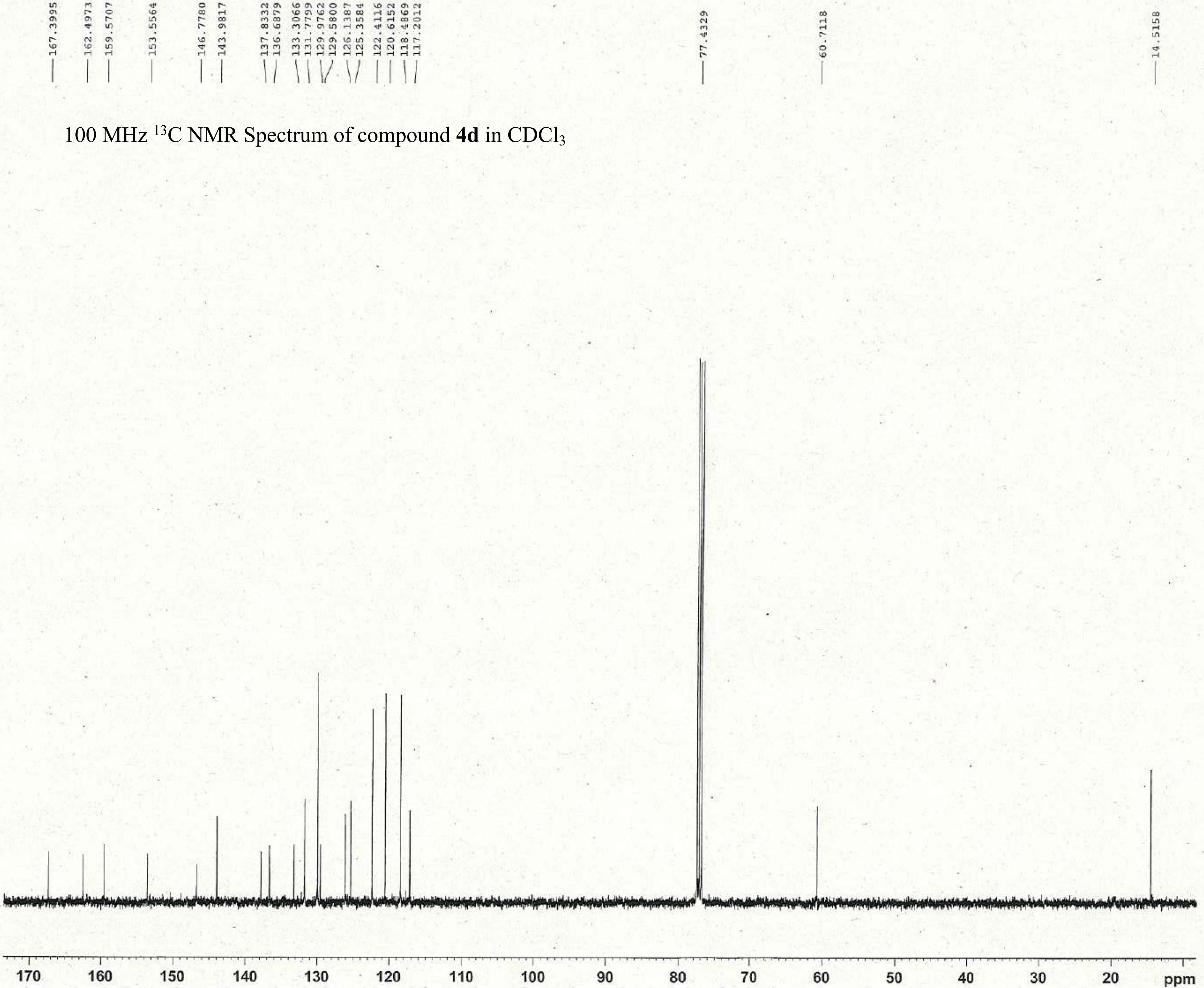

400 MHz ^{1}H NMR Spectrum of compound **12** in $CDCl_3$

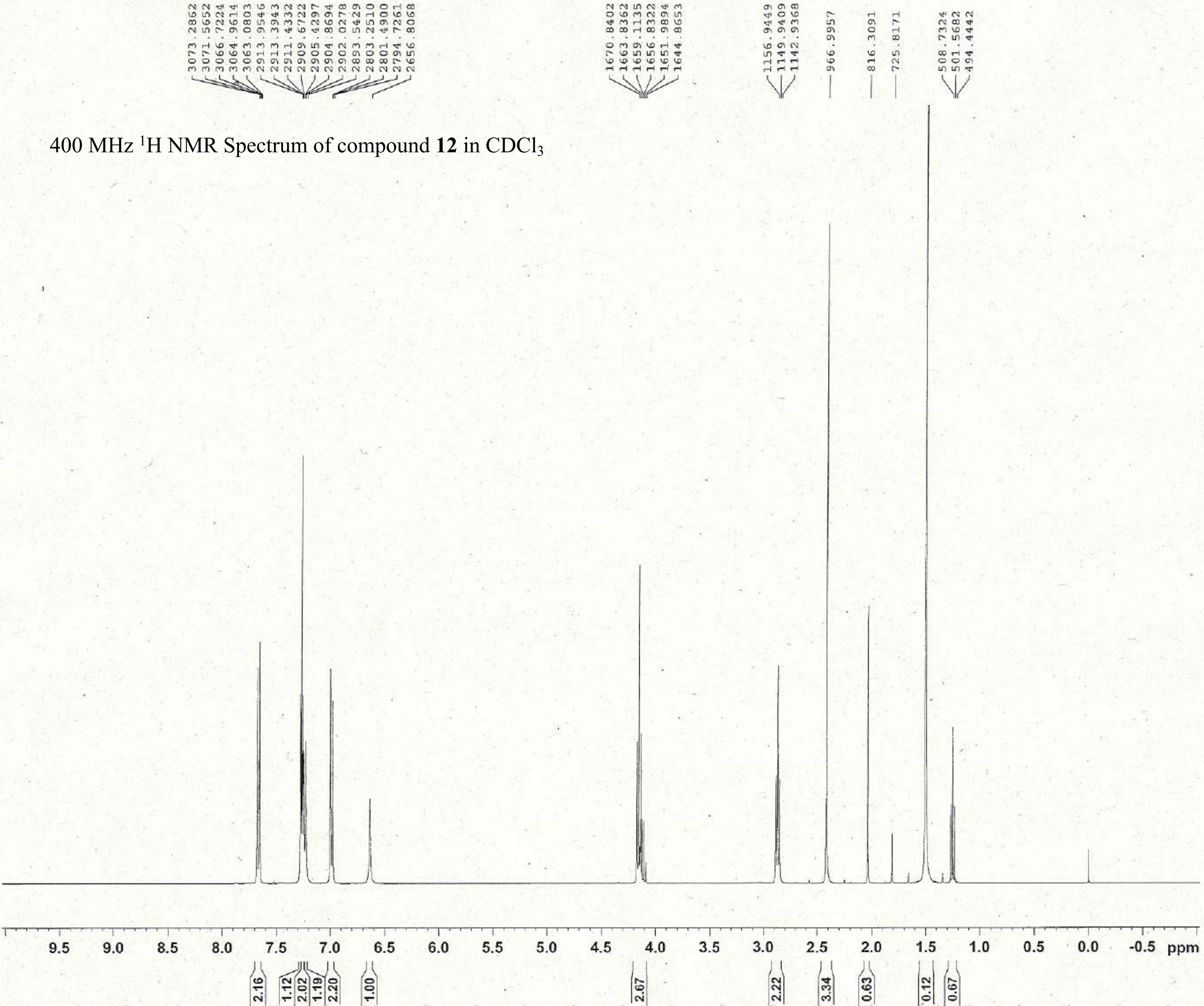

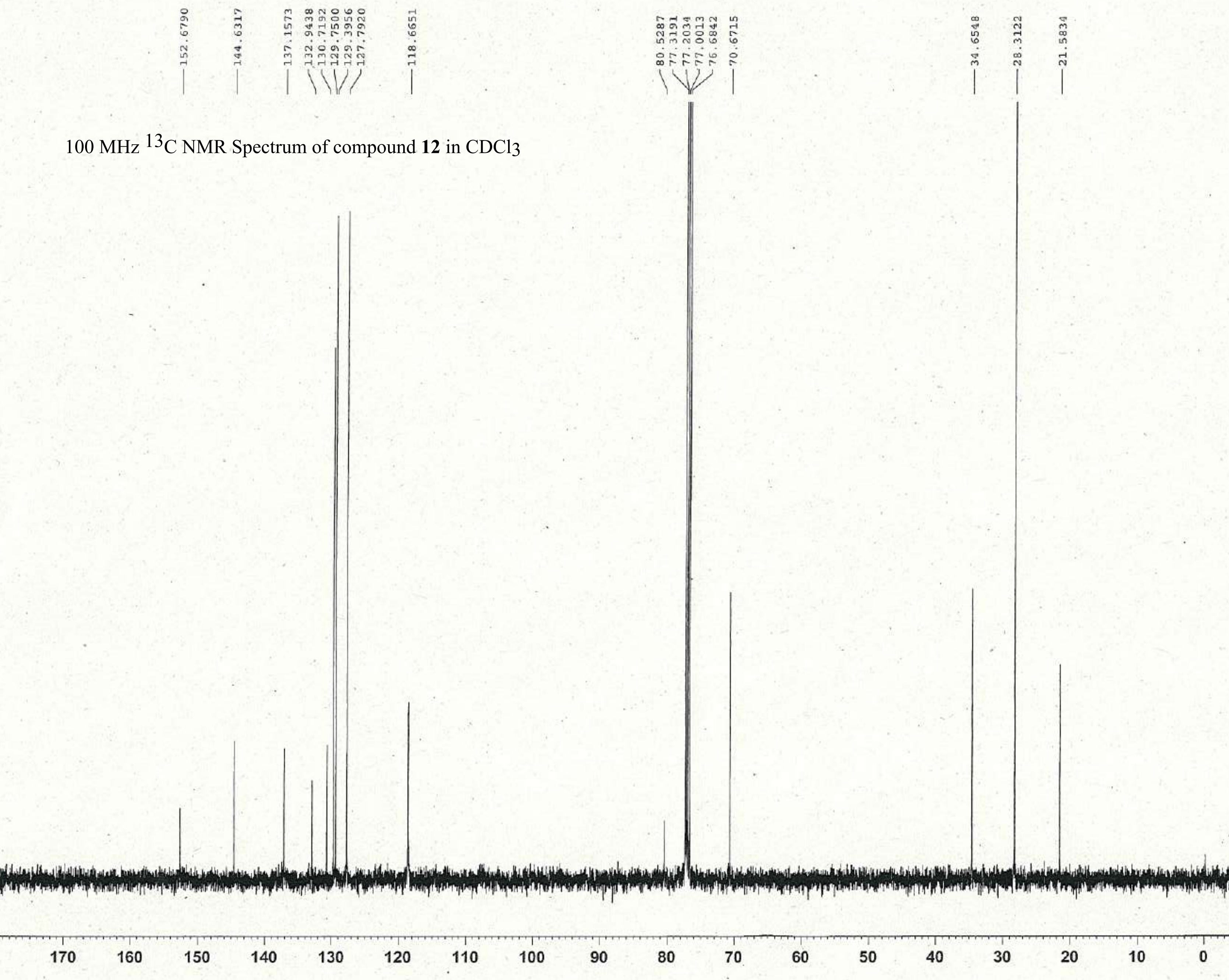

100 MHz ^{13}C NMR Spectrum of compound **12** in $CDCl_3$

400 MHz ^{1}H NMR Spectrum of compound **13** in $CDCl_3$

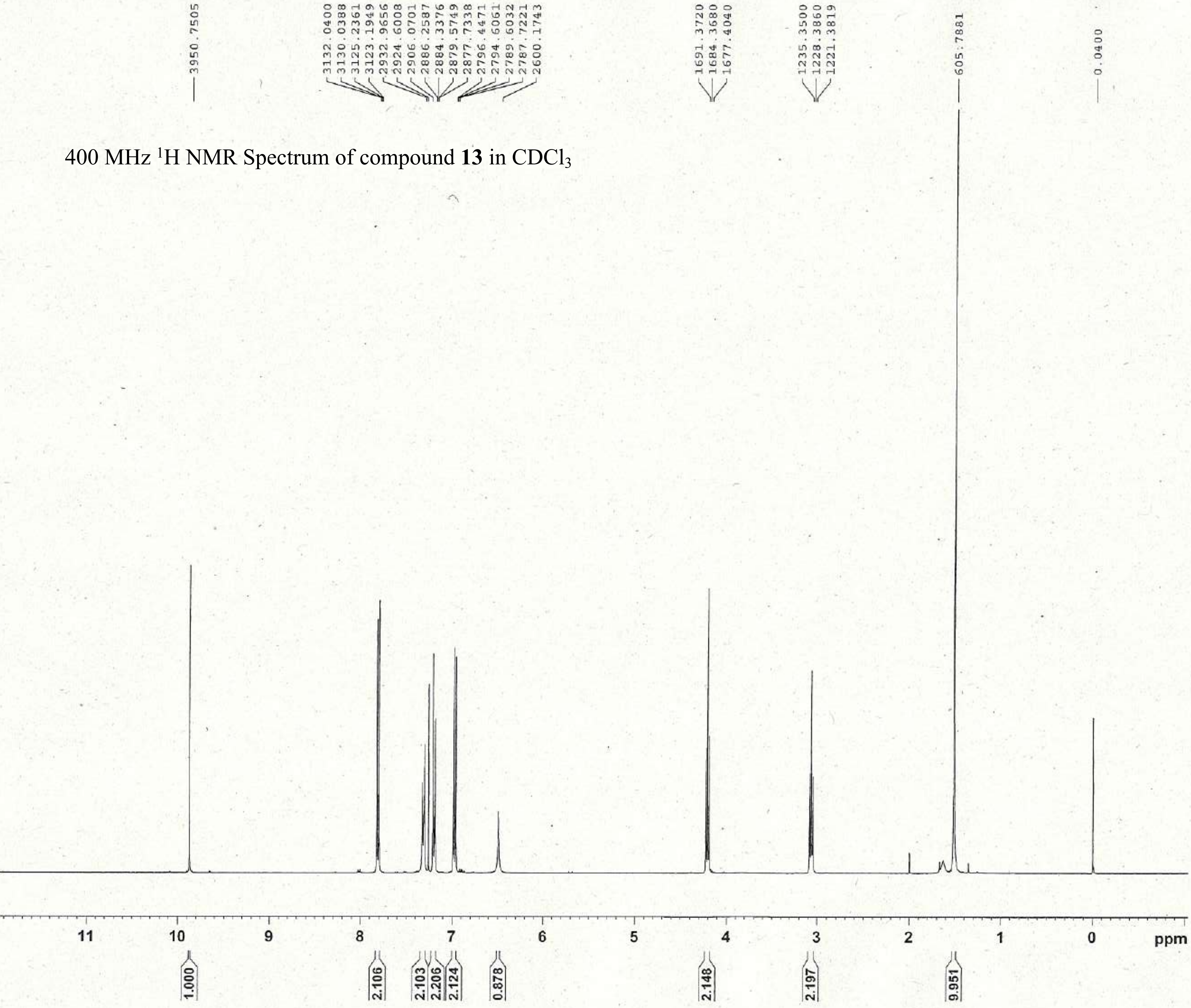

100 MHz ^{13}C NMR Spectrum of compound **13** in $CDCl_3$

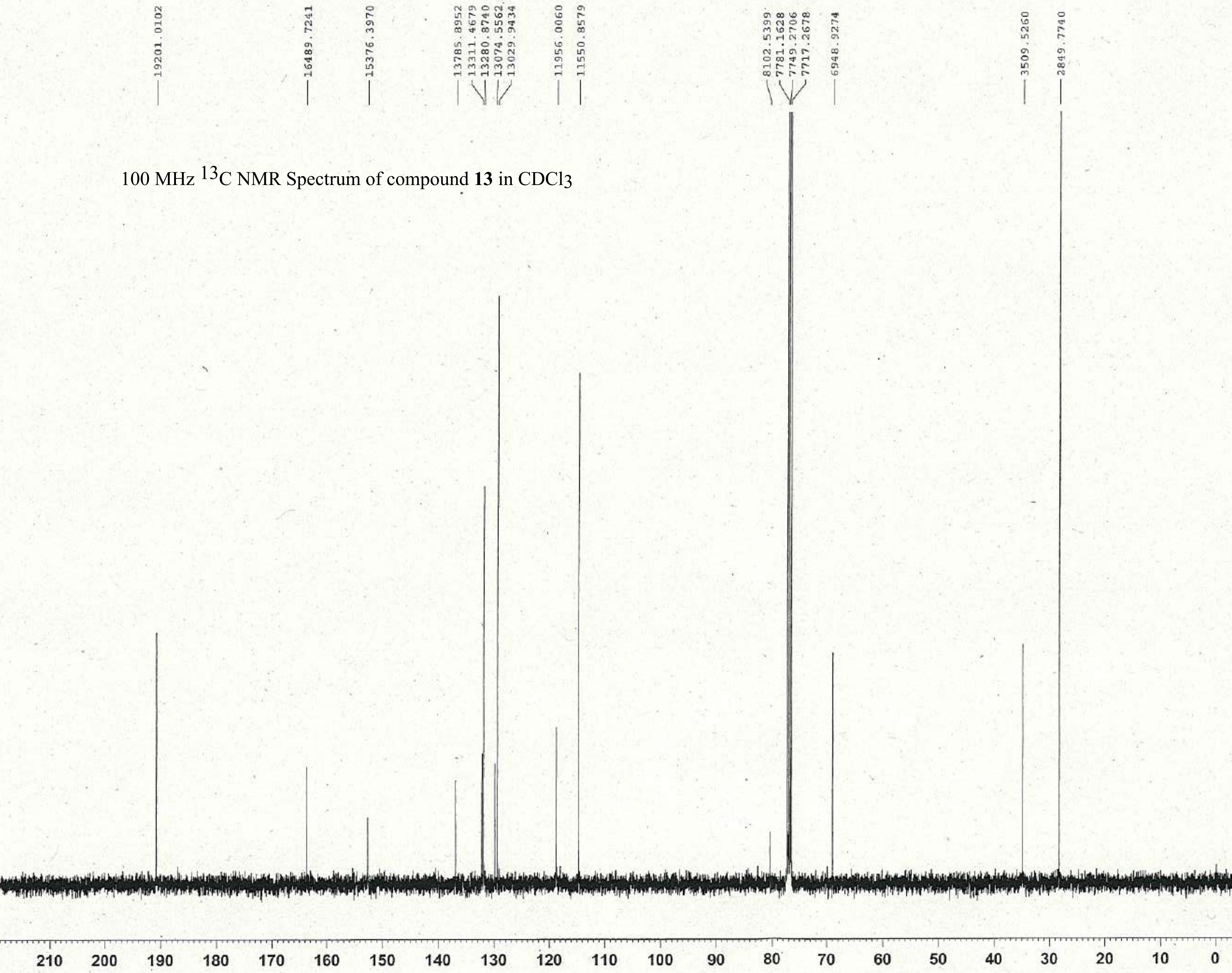

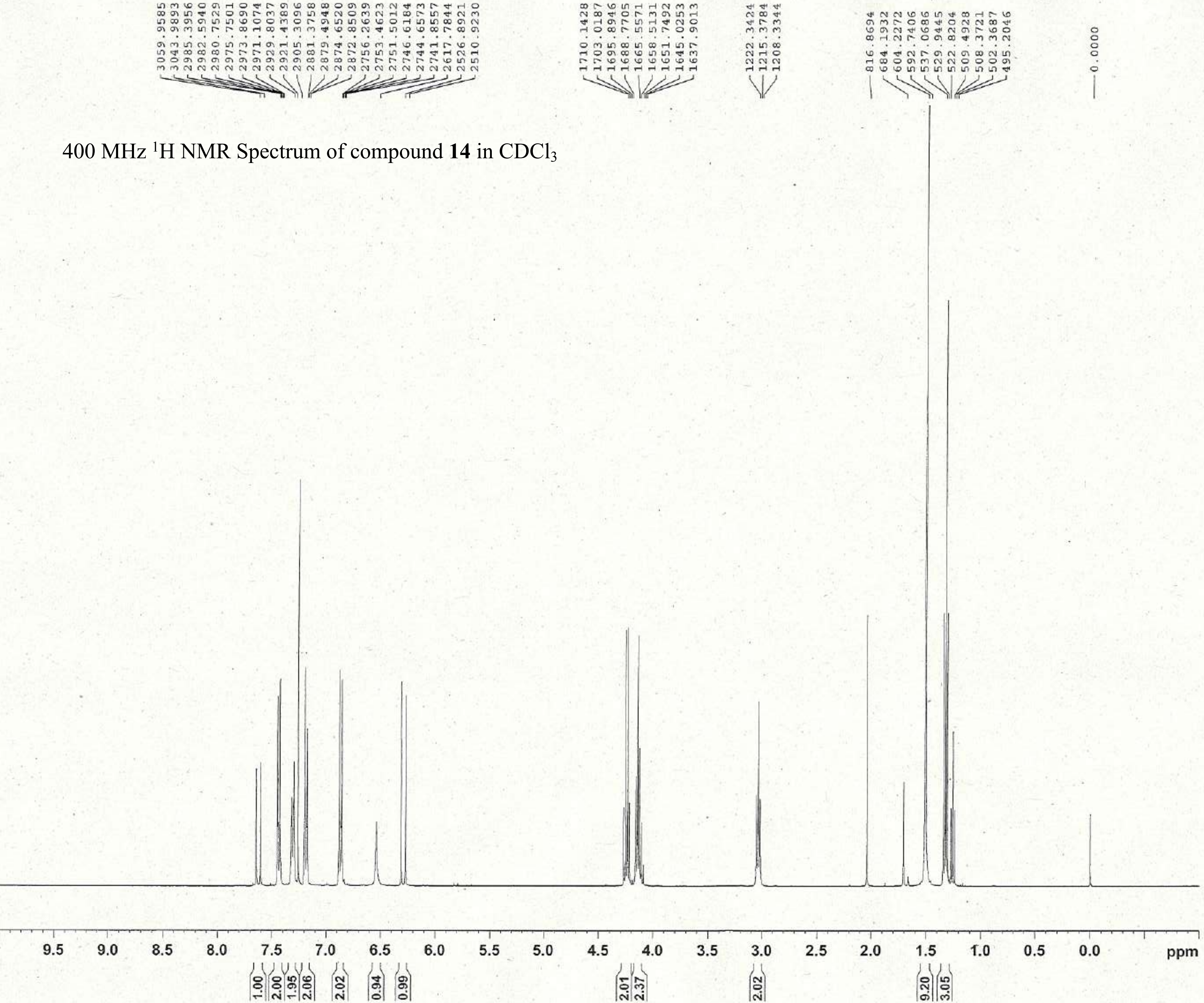

400 MHz ^{1}H NMR Spectrum of compound **14** in $CDCl_3$

100 MHz ^{13}C NMR Spectrum of compound **14** in $CDCl_3$

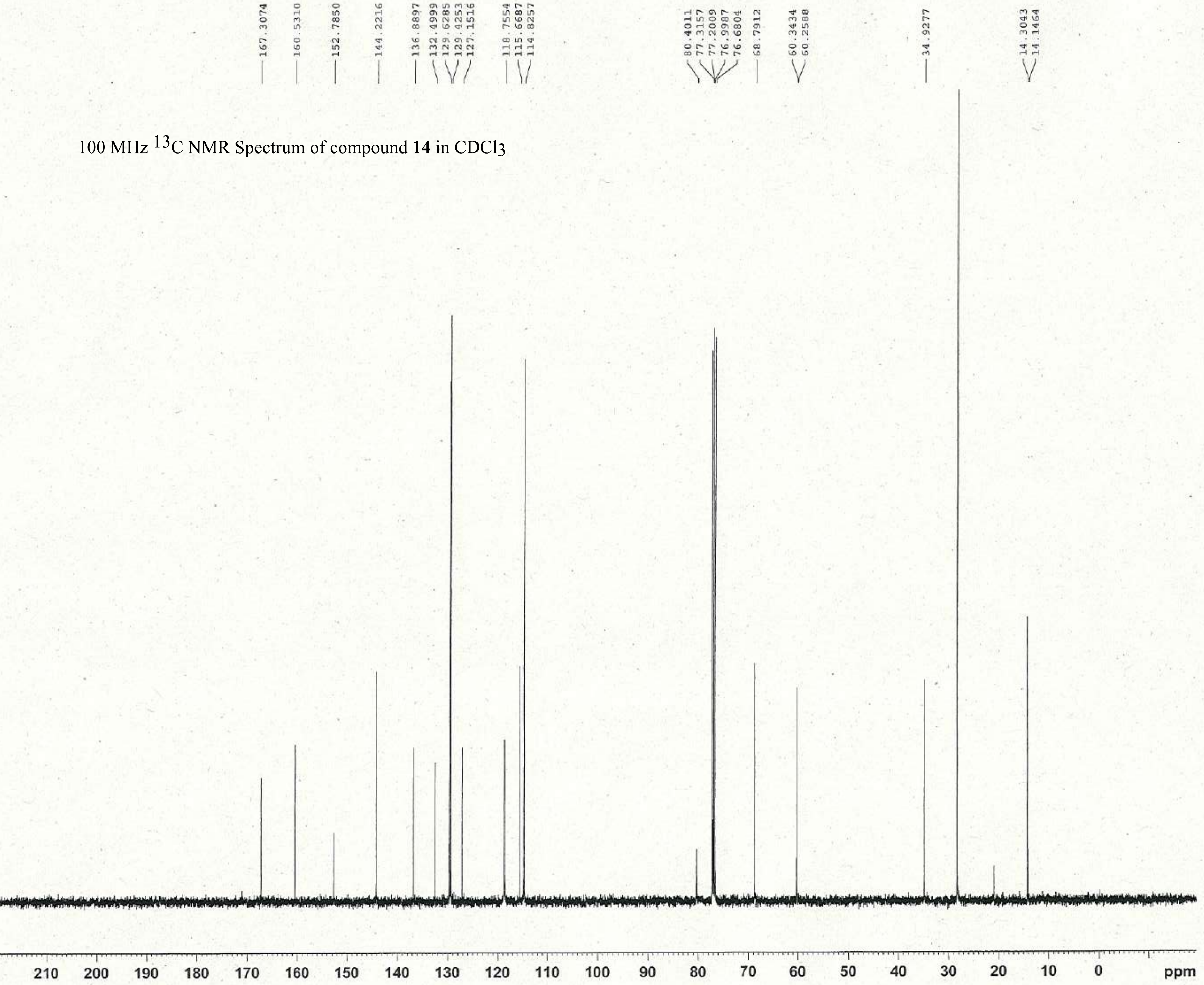

400 MHz ^{1}H NMR Spectrum of compound **15** in $CDCl_3$

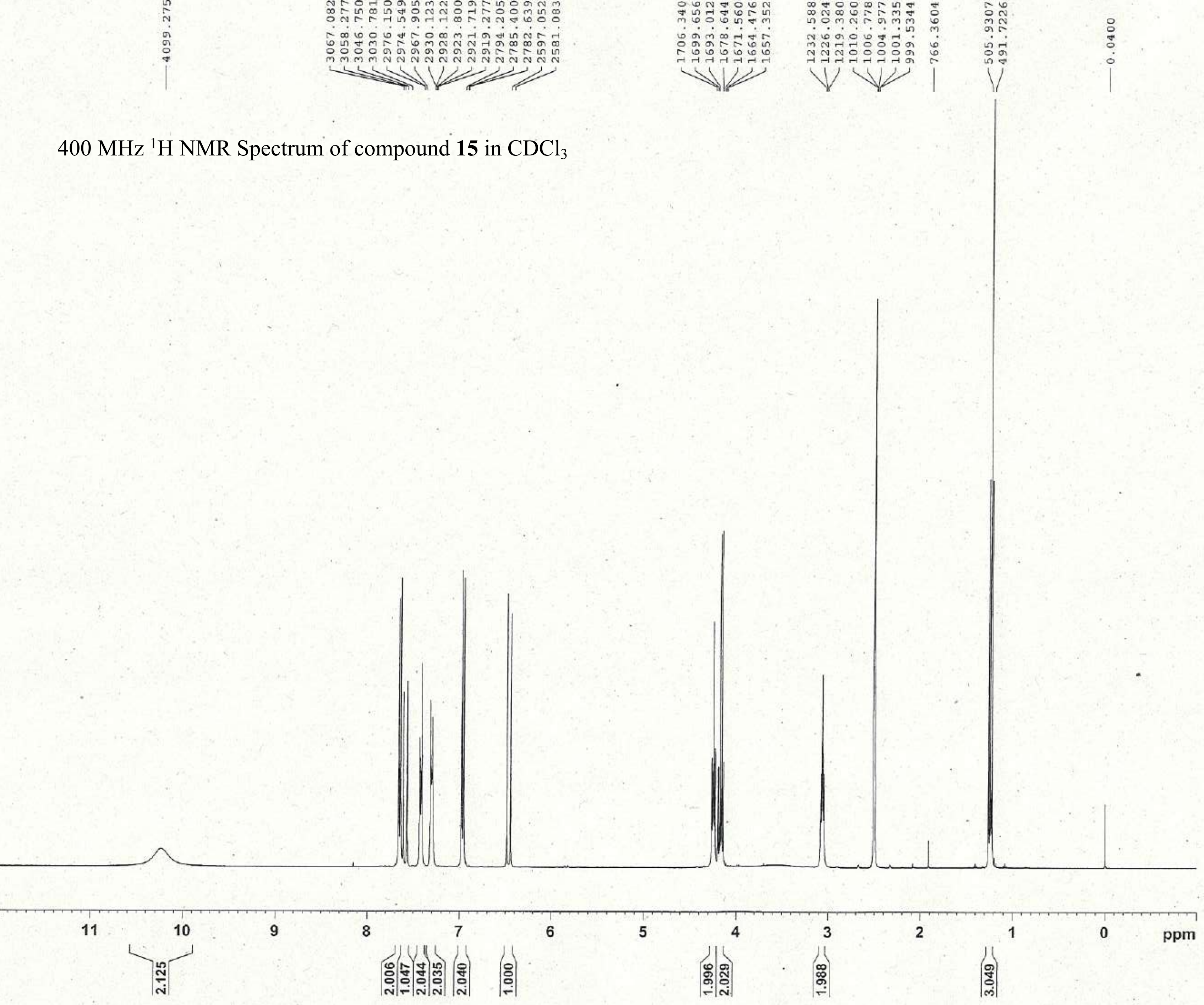

100 MHz ^{13}C NMR Spectrum of compound **15** in $CDCl_3$

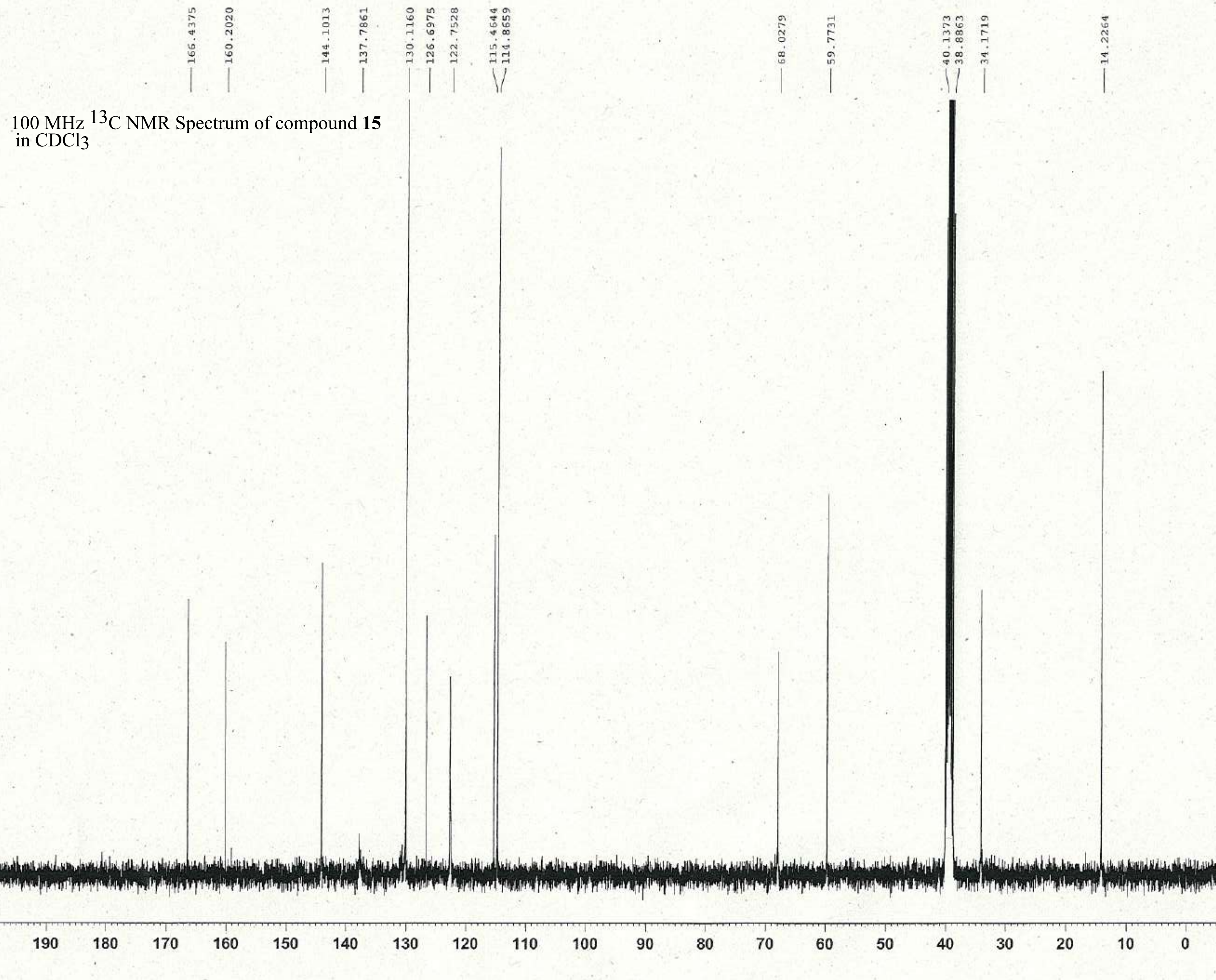

400 MHz ^{1}H NMR spectrum of compound **16** in $CDCl_3$

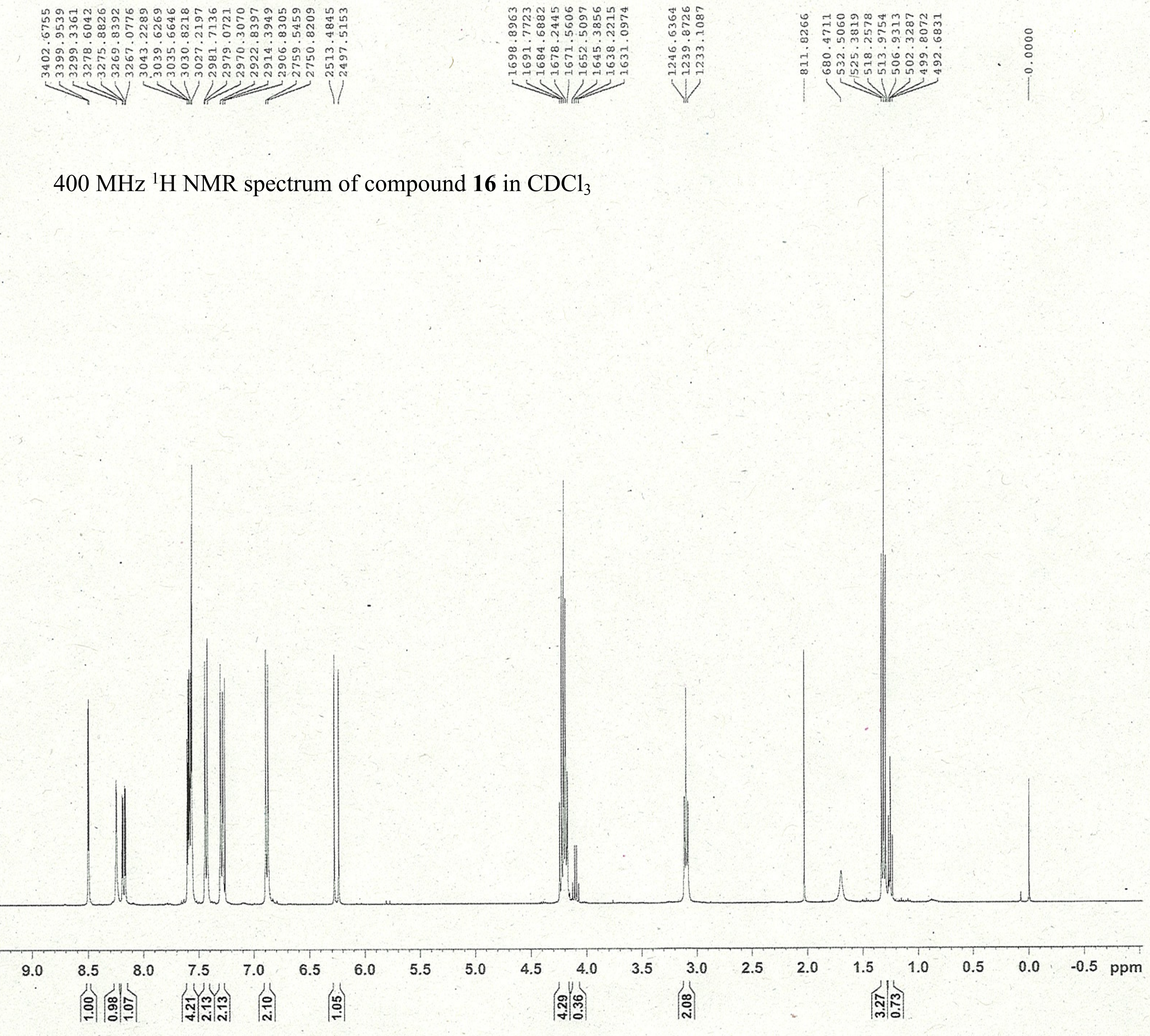

100 MHz ^{13}C NMR spectrum of compound **16** in $CDCl_3$

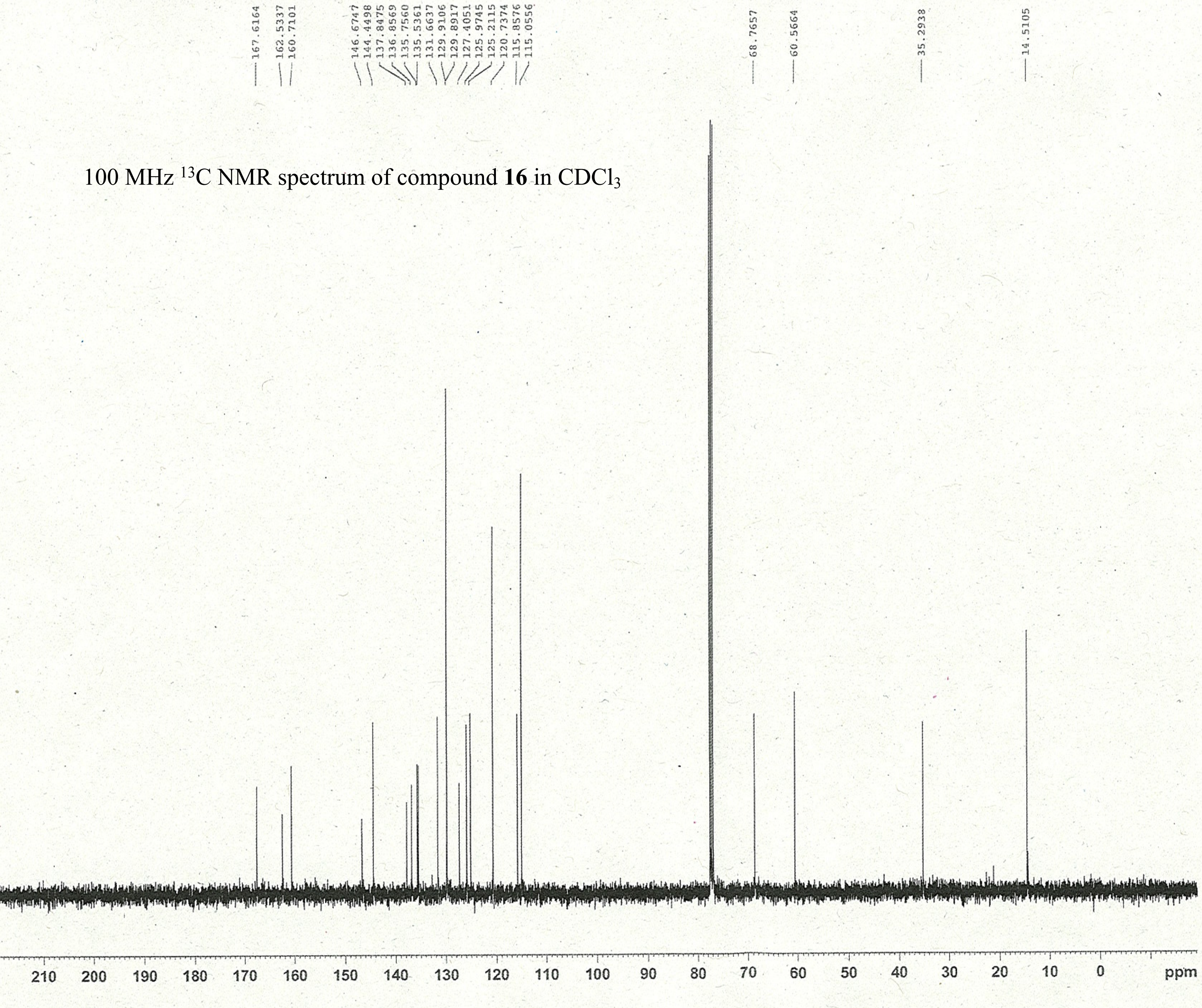

Supplement: Supplementary file 1 [file molecules-24-02019-s001.pdf]
